# Supplementary material for: X‐linked inhibitor of apoptosis protein represents a promising therapeutic target for relapsed/refractory ALL
Source: EMBO Mol Med. 2022 Nov 23;15(1):e14557. doi: 10.15252/emmm.202114557 (PMC9832863; doi:10.15252/emmm.202114557)
Supplement: Supplementary file 1 — Appendix [file EMMM-15-e14557-s003.pdf]

# Appendix

# X-linked inhibitor of apoptosis protein represents a promising therapeutic target for relapsed/refractory ALL

Running Title: Targeting XIAP in r/r ALL

Michela Carlet[1,2*], Karin Schmelz[3,4*], Jenny Vergalli[1*], Tobias Herold[1,5,6], Daniela Senft [1], Vindi Jurinovic[1,5,,7], Thomas Hoffmann[8], Jutta Proba[3], Nina Weichert[3], Christian Junghanß[9], Mareike Roth[8], Georg Eschenburg[10], Malwine Barz[11], Günter Henze[3], Cornelia Eckert[3], Angelika Eggert[3], Johannes Zuber[9], Patrick Hundsdoerfer[3,12,13#] and Irmela Jeremias[1,6,7#☆]

<u>This file contains</u>:

Appendix Tables (3)

Appendix Figures and Legends (3)

**Appendix Table S1:** Combination indices (CI) values of LBW242 at concentrations applied in combination with vincristine, doxorubicin, L-Asparaginase and dexamethasone in LOUCY cells.

| LBW (µM) | VCR (ng/ml) | | | Doxo (µM) | | | L-Asp (IE/ml) | | | Dexa (µM) |
|---|---|---|---|---|---|---|---|---|---|---|
| | CI ($EC_{60}$) | CI ($EC_{50}$) | CI ($EC_{25}$) | CI ($EC_{50}$) | CI ($EC_{25}$) | CI ($EC_{10}$) | CI ($EC_{60}$) | CI ($EC_{50}$) | CI ($EC_{25}$) | CI ($EC_{20}$) |
| 5 | 0,76 | 0,81 | 0,81 | n.d. | n.d. | 2,67 | 0,34 | 0,96 | 0,97 | 0,45 |
| 10 | 0,73 | 0,78 | 0,84 | n.d. | n.d. | 4,72 | 0,39 | 0,97 | 1,04 | 0,55 |
| 20 | 0,74 | 0,78 | 0,85 | n.d. | n.d. | 8,51 | 0,47 | 0,91 | 0,98 | 0,53 |

Combination indices (CI) with synergism (CI<1), additivity (CI=1) or antagonism (CI>1) were calculated as described in material and methods using the Chou-Talalay method. n.d.: not determinded.

**Appendix Table S2:** Combination indices (CI) values of LBW242 at concentrations applied in combination with vincristine, doxorubicin, L-Asparaginase and prednisone in NALM-6 cells.

| LBW (µM) | VCR (ng/ml) | | | Doxo (µM) | | | L-Asp (IE/ml) | | | Predni (µM) |
|---|---|---|---|---|---|---|---|---|---|---|
| | CI ($EC_{60}$) | CI ($EC_{50}$) | CI ($EC_{25}$) | CI ($EC_{50}$) | CI ($EC_{25}$) | CI ($EC_{10}$) | CI ($EC_{60}$) | CI ($EC_{50}$) | CI ($EC_{25}$) | CI ($EC_{10}$) |
| 5 | 0,71 | 0,57 | 0,71 | 0,99 | 0,73 | n.d. | 0,45 | 0,23 | 0,87 | 0,62 |
| 10 | 0,77 | 0,63 | 0,74 | 0,97 | 0,72 | n.d. | 0,25 | 0,31 | 0,82 | 0,65 |
| 20 | 0,74 | 0,66 | 0,78 | 0,92 | 0,29 | n.d. | 0,34 | 0,51 | 1,01 | 0,95 |

Combination indices (CI) with synergism (CI<1), additivity (CI=1) or antagonism (CI>1) were calculated as described in material and methods using the Chou-Talalay method. n.d.: not determinded.

**Appendix Table S3**: Exact p-values of the data displayed in the main figures and expanded views .

| | | | | | | | | |
|---|---|---|---|---|---|---|---|---|
| Figure 1A | Student's t-test | | | | | | | |
| CTRL vs treated | 0 | 0,5ng/ml VCR | 1ng/ml VCR | | | | | |
| 2,5 µM BV6 | 0,084963905 | **0,000582774** | **0,001029077** | | | | | |
| 20 µM LCL | 0,281727762 | **5,35344E-06** | **0,000140644** | | | | | |
| 20 µM LBW | **0,000308131** | **1,19497E-05** | **2,96861E-05** | | | | | |
| Figure 1C | Student's t-test | | | | | | | |
| CTRL vs treated | 0 | 1ng/ml VCR | 2,5ng/ml VCR | 0.25IE/ml L-Asp | 10 µM Dexa | | | |
| 20 µM LBW | **0,0130** | **0,0009** | **0,0003** | **0,0082** | **0,0026** | | | |
| Figure 1D | Student's t-test | | | | | | | |
| CTRL vs treated | 0 | 1ng/ml VCR | 2,5ng/ml VCR | 0.25IE/ml L-Asp | 10 µM Dexa | | | |
| 20 µM LBW | **0,000369316** | **4,1109E-05** | **1,13E-05** | **0,000733103** | **0,000353214** | | | |
| Figure 1E | Student's t-test | | | | | | | |
| CTRL vs treated | 0 | 1ng/ml VCR | 2,5ng/ml VCR | 0.25IE/ml L-Asp | 10 µM Dexa | | | |
| 20 µM LBW | **0,0012** | **0,0000** | **0,0001** | **0,0001** | **0,0034** | | | |
| Figure 2A | Student's t-test | | | | | | | |
| CTRL vs treated | 0 | 1ng/ml VCR | 20µM LBW | VCR1+20LBW | 10ng/mlTNF | 20LBW+10TNF | | |
| 100 µM Nec | 0,0580 | **0,0097** | 0,0698 | 0,1011 | 0,1878 | **0,0170** | | |
| Figure 2B | Student's t-test | | | | | | | |
| CTRL vs KD | 0 | 1ng/ml VCR | 20µM LBW | VCR1+20LBW | 10ng/mlTNF | 20LBW+10TNF | | |
| negsiRNA/RIP1KsiRNA | 0,3913 | 0,9423 | 0,1745 | 0,0964 | 0,2336 | **0,0004** | | |
| Figure 2D | Student's t-test | | | | | | | |
| CTRL vs KD | 0 | 2,5ng/ml VCR | 20µM LBW | VCR2,5+20LBW | | | | |
| negsiRNA/NEMOsiRNA | **0,04811472** | 0,367441258 | 0,059994962 | 0,681266411 | | | | |
| Figure 3A | Games-Howell | | | | | | | |
| ctrl vs. AML | **0.00964** | | | | | | | |
| ctrl vs. ALL | **1,96E-05** | | | | | | | |
| AML vs. ALL | **4,24E-09** | | | | | | | |
| Figure 4H | Student's t-test | | | | | | | |
| shCTRL vs shXIAP.1 | **0,00873** | | | | | | | |
| Figure EV1A | Student's t-test | | | | | | | |
| CTRL vs treated | 0 | 1 ng/ml VCR | 2,5ng/ml VCR | 20ng/ml TNF | 20µM LBW | 1 VCR+20LBW | 2,5VCR+20LBW | 20TNF+20LBW |
| 10µg/ml ADM | 0,292812641 | 0,777831278 | 0,870322417 | 0,870407399 | 0,717239546 | 0,491204268 | 0,414450383 | **5,63691E-05** |
| Figure EV1D | Student's t-test | | | | | | | |
| CTRL vs treated | 0 | 20ng/ml TNF | 20µM LBW | 20TNF+20LBW | | | | |
| 10µg/ml ADM | 0,969276919 | 0,526891977 | 0,93525466 | **0,000294946** | | | | |
| Figure EV1F | Student's t-test | | | | | | | |
| CTRL vs treated | 0 | 2,5ng/ml VCR | 20µM LBW | 2,5 VCR+ 20LBW | | | | |
| 30µM Nec | 0,85439 | **0,00536** | 0,23620 | 0,13289 | | | | |
| Figure EV1G | Student's t-test | | | | | | | |
| CTRL vs treated | 0 | 20ng/ml TNF | 20µM LBW | 20TNF+20LBW | | | | |
| 30µM Nec | 0,052883274 | 0,101156039 | 0,292082893 | **0,017500033** | | | | |

**Appendix Table S3**: Exact p-values of the data displayed in the appendix figures.

| Figure S1A | Student's t-test | | | | | | | |
|---|---|---|---|---|---|---|---|---|
| CTRL vs treated | 10µM LBW | | | | | | | |
| Molt-4 | 1 | | | | | | | |
| Molt-3 | 1 | | | | | | | |
| Nalm-6 | **0,00363241** | | | | | | | |
| CEM-C1 | **0,0494052** | | | | | | | |
| Loucy | **0,00351995** | | | | | | | |
| CTV-1 | **0,00480327** | | | | | | | |
| Reh | **2,49647E-06** | | | | | | | |
| Molt-16 | **0,000612923** | | | | | | | |
| UoC-B6 | **0,000115696** | | | | | | | |
| Figure S1D | Student's t-test | | | | | | | |
| CTRL vs treated | 0 | 0,5ng/ml VCR | 1ng/ml VCR | | | | | |
| 2,5 µM BV6 | **0,011012234** | **0,000442628** | **3,52E-05** | | | | | |
| 20 µM LCL | **0,011156842** | **0,001224023** | **0,000581156** | | | | | |
| Figure S1E | Student's t-test | | | | | | | |
| CTRL vs treated | 0 | 0,5ng/ml VCR | 1ng/ml VCR | | | | | |
| 10 µM LBW | **0,000349609** | **0,004646706** | **0,007630418** | | | | | |
| 20 µM LBW | **0,008804797** | **0,015317905** | **0,015304673** | | | | | |
| CTRL vs treated | 0 | 0,016 | 0,03125 | 0,0625 | µM Doxo | | | |
| 10 µM LBW | **0,033481909** | **0,000143641** | **0,027568743** | 0,805117584 | | | | |
| 20 µM LBW | **0,010891842** | **0,009356303** | **0,004489487** | 0,134202301 | | | | |
| CTRL vs treated | 0 | 0,1 | 0,25 | 0,5 | IE/ml Asp | | | |
| 10 µM LBW | **0,033481909** | **0,000378345** | **0,008107675** | **0,003487407** | | | | |
| 20 µM LBW | **0,010891842** | **3,51491E-05** | **0,000845131** | **0,002932937** | | | | |
| CTRL vs treated | 0 | 0,016 | 0,03125 | 0,0625 | µM Predni | | | |
| 10 µM LBW | **0,020100403** | **0,025778705** | 0,05801541 | 0,242626027 | | | | |
| 20 µM LBW | **0,033109433** | **0,002592965** | **0,026476042** | **0,000228633** | | | | |
| Figure S1F | Student's t-test | | | | | | | |
| CTRL vs treated | 0 | 0,5ng/ml VCR | 1ng/ml VCR | 2,5 ng/ml VCR | | | | |
| 10 µM LBW | **0,003512038** | **0,001635095** | **0,000752515** | **0,0045211** | | | | |
| 20 µM LBW | **0,00593424** | **0,000753875** | **0,0083219** | **0,000485581** | | | | |
| CTRL vs treated | 0 | 0,016 | 0,03125 | 0,0625 | µM Doxo | | | |
| 10 µM LBW | **0,009080952** | **0,010852514** | 0,281395048 | 0,460737129 | | | | |
| 20 µM LBW | **0,018791506** | 0,125144237 | **0,001543763** | **0,001103613** | | | | |
| CTRL vs treated | 0 | 0,1 | 0,25 | 0,5 | IE/ml Asp | | | |
| 10 µM LBW | **0,009080952** | **0,018587429** | **0,009592439** | **0,002883264** | | | | |
| 20 µM LBW | **0,018791506** | **0,00022726** | **0,000737238** | **0,003574289** | | | | |
| CTRL vs treated | 0 | 1 | 5 | 10 | µM Dexa | | | |
| 10 µM LBW | **0,003512038** | **0,006437393** | **0,003387514** | 0,051182878 | | | | |
| 20 µM LBW | **0,00593424** | **0,001626988** | **0,006609678** | **0,01059855** | | | | |

**Appendix Table S3**: Exact p-values of the data displayed in the appendix figures

| Figure S1G | Student's t-test | | | | | | | |
|---|---|---|---|---|---|---|---|---|
| CTRL vs treated | 0 | 1 ng/ml VCR | 2,5 ng/ml VCR | 0.25 L-Asp (IE/ml) | 10 Dexa (µM) | | | |
| 20µM LBW | **0,000163436** | **0,020126905** | **0,041600748** | **0,000229908** | **9,77228E-05** | | | |
| Figure S1I | Student's t-test | | | | | | | |
| CTRL vs treated | 0 | 1 VCR | 2,5 VCR | 0.25 L-Asp | 5 µM Predni | | | |
| 20µM LBW | 0,0802 | **0,0000** | **0,0004** | **0,0003** | **0,0005** | | | |
| Figure S1J | Student's t-test | | | | | | | |
| CTRL vs treated | 0 | 1 VCR | 2,5 VCR | 0.25 L-Asp | 5 µM Predni | | | |
| 20µM LBW | **0,0340** | **0,0000** | **0,0003** | **0,0050** | **0,0006** | | | |
| Figure S1K | Student's t-test | | | | | | | |
| CTRL vs treated | 0 | 1 VCR | 2,5 VCR | 0.25 L-Asp | 5 µM Predni | | | |
| 20µM LBW | **0,0085** | **0,0042** | **0,0184** | **0,0024** | **0,0024** | | | |
| Figure S2D | Student's t-test | | | | | | | |
| CTRL vs treated | 0 | 0.5ng/ml VCR | 1ng/ml VCR | 10µM Dexa | | | | |
| 10 µM A4 | **0,0192** | **0,0000** | **0,0007** | **0,0218** | | | | |
| CTRL vs treated | 0 | 0.5ng/ml VCR | 1ng/ml VCR | 10µM Dexa | | | | |
| 10 µM A4 | **0,0130** | **0,0006** | **0,0072** | **0,0004** | | | | |
| Figure S3I | Student's t-test | | | | | | | |
| shCTRL vs shXIAP.1 | **0,009** | | | | | | | |
| shXIAP.1 vs shXIAP/OE | **0,0017** | | | | | | | |
| shCTRL vs shXIAP/OE | **0,0025** | | | | | | | |

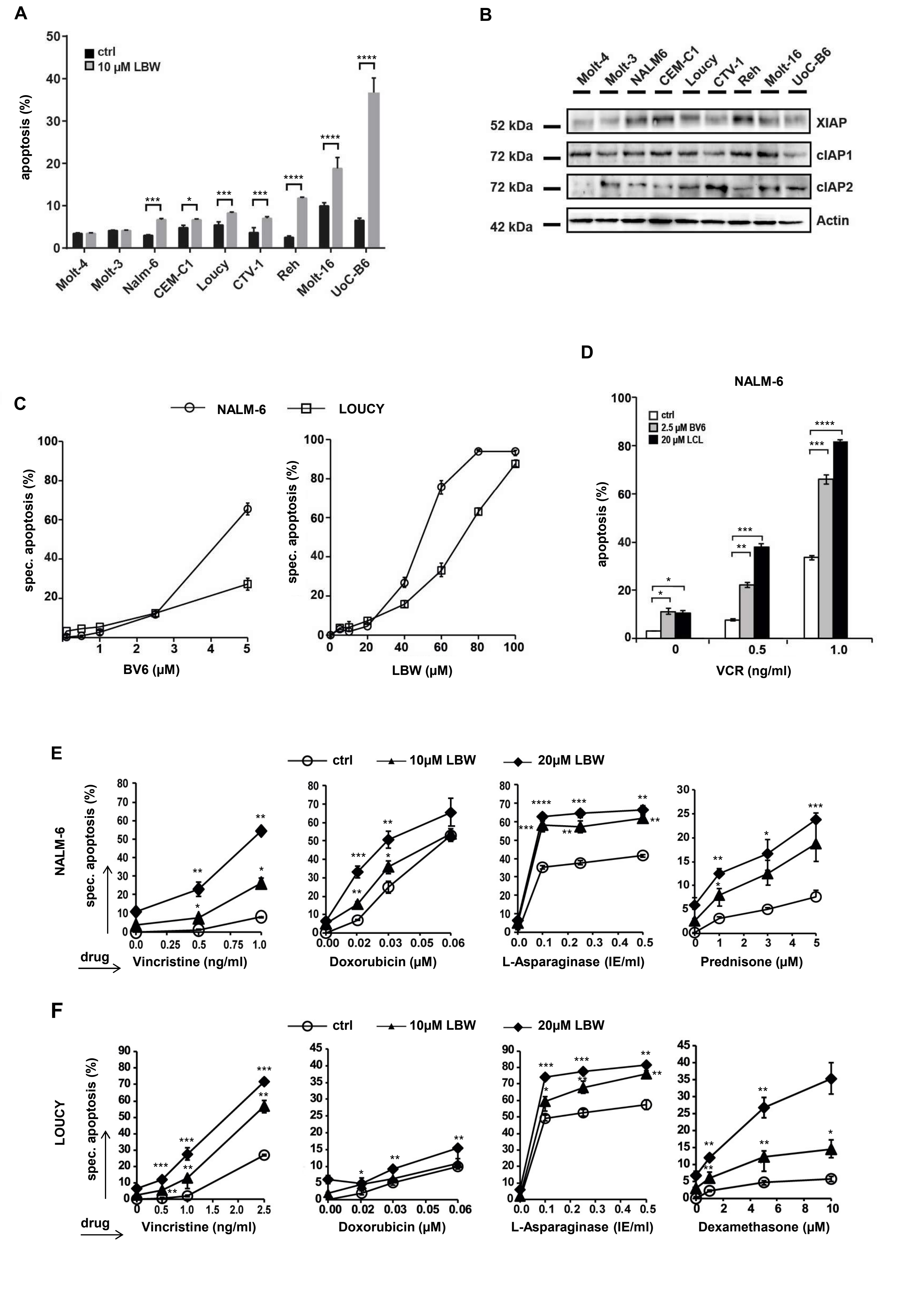


**Appendix Figure S1**

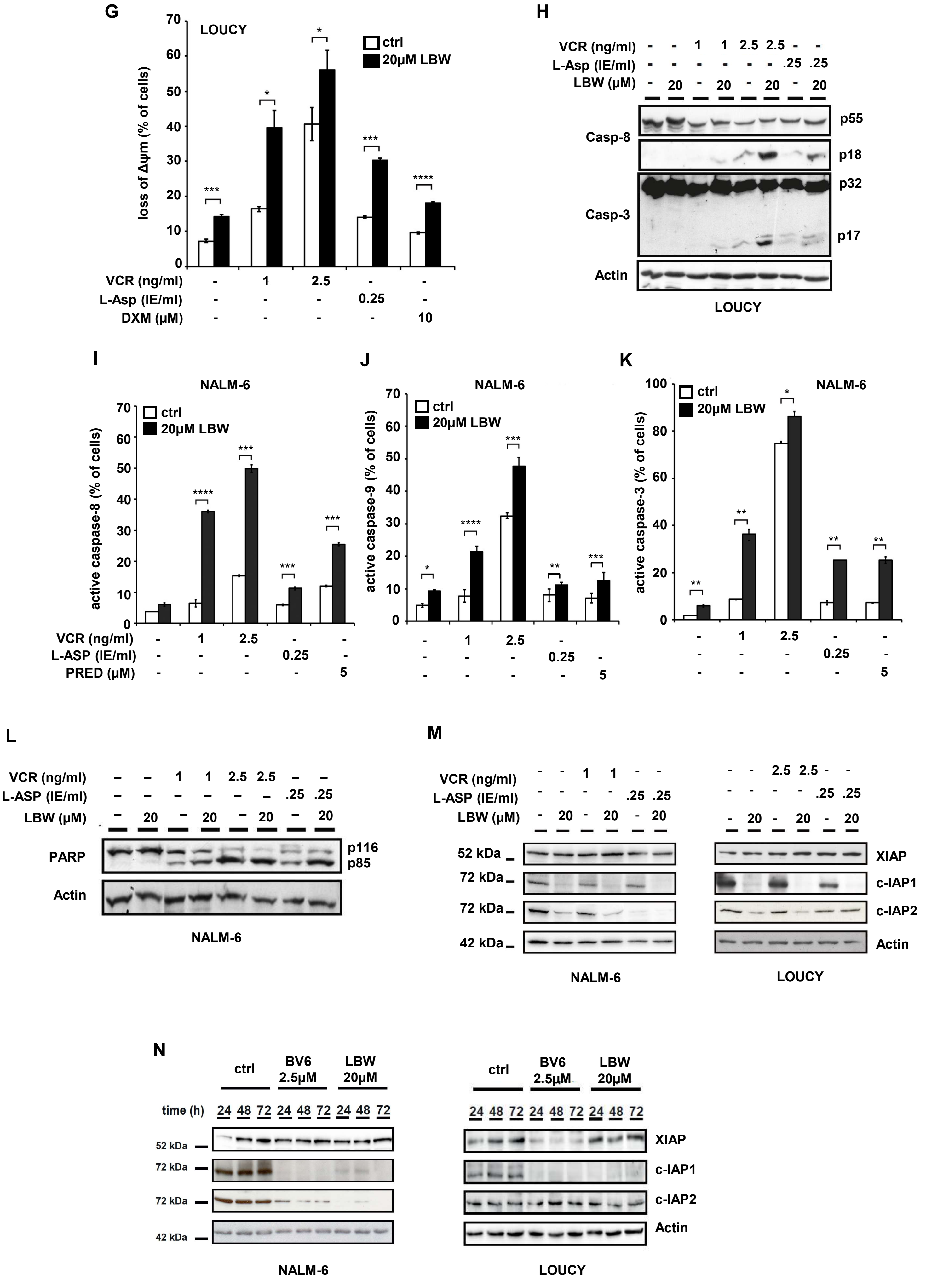


Appendix Figure S1

**Appendix Figure S1: SM sensitize r/r ALL to cytotoxic drugs**

**A** Apoptosis induction by SM (LBW242,10µM) in the indicated ALL cell lines determined 48h after treatment by flow cytometry. Mean ± SEM of 3 independent experiments with * p <0.05 , *** p <0.001, **** p <0.0001 unpaired Student's t-test is shown.
**B** Abundance of IAP in different ALL cell lines. Protein levels of XIAP and c-IAP1/2 were analyzed by Western blot. Actin served as loading control. One representative immunoblot out of 3 independent experiments is shown.
**C** Limited sensitivity towards SM alone. NALM-6 and LOUCY cells were treated with the indicated SM for 48h. Apoptosis was measured following staining with Annexin-V/propidium iodide. Mean ± SEM of 3 independent experiments is shown.
**D** Synergistic cell death of SM in combination with cytotoxic drugs. Apoptosis induction by different SMs in combination with vincristine (VCR) in NALM-6 cells. Flow cytometry analysis (Annexin-V/propidium iodide) after 48h of treatment is depicted. Mean ± SEM of 3 independent experiments with * p <0.05, ** p <0.01, *** p <0.001, **** p <0.0001 by unpaired Student's t-test is shown.
**E-F** NALM-6 (**E**) and Loucy (**F**) cells were treated for 48h with the indicated concentrations of LBW242, VCR, doxorubicin, L-asparaginase (L-ASP) , prednisone or dexamethasone. Apoptosis was determined by flow cytometry after Annexin V and PI staining. Background apoptosis of untreated control cells was subtracted. Mean ± SEM of 3 independent experiments with * p <0.05, ** p <0.01, *** p <0.001, ****p <0.0001 by unpaired Student's t-test is shown.
**G** Mitochondrial depolarization was analyzed using flow cytometry by detection of the loss of mitochondrial membrane potential ($\Delta\psi_m$) in LOUCY ALL cells. Mean ± SEM of 3 independent experiments with * p <0.05, *** p <0.001, **** p <0.0001 by unpaired Student's t-test is shown.
**H** Caspase-8 and Caspase-3 activation in LOUCY cells was determined using Western blotting of the active (p18) caspase-8 subunit and the active p17 caspase-3 subunit 48h after treatment with the indicated drugs. One representative immunoblot out of 3 independent experiments is shown.
**I-K** Caspase activation. Activation of (**I**) Caspase 8, (**J**) Caspase-9, (**K**) Caspase-3 was analyzed by flow cytometry 48h after treatment of NALM-6 cells with the indicated drugs as described in Figure 1. Mean ± SEM of 3 independent experiments with * p <0.05, ** p <0.01, *** p<0.001, **** p <0.0001 by unpaired Student's t-test is shown.
**L** Cleavage of PARP was measured by Western Blot in NALM-6 cells 48h after the indicated treatment. Actin was used as loading control. One representative immunoblot out of 3 independent experiments is shown.
**M** SM and cytotoxic drugs degrade cIAP-1/2, but not XIAP. XIAP, cIAP-1 and cIAP-2 protein levels were determined after treatment of NALM-6 and LOUCY cells with VCR, L-ASP and LBW242 (LBW) for 24h by Western blot. Actin served as loading control. One representative immunoblot out of 3 independent experiments is shown.
**N** Protein abundance of XIAP, cIAP-1 and cIAP-2 after treatment of NALM-6 and LOUCY cells with SM at the indicated concentrations was detected by Western blot analysis. Actin served as loading control. One representative immunoblot out of 3 independent experiments is shown.
Source data for Appendix and Expanded View are available online.

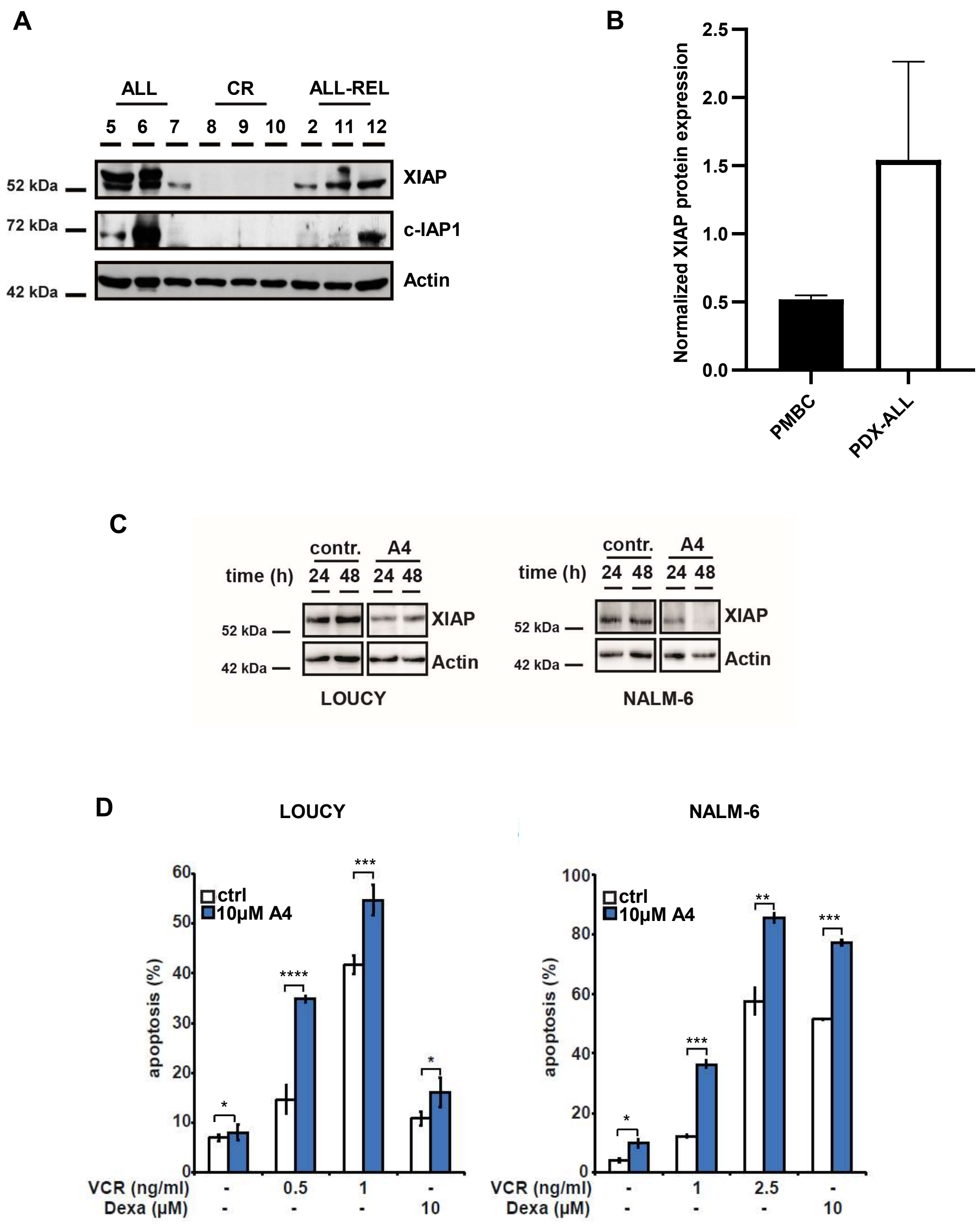


## Appendix Figure S2: XIAP expression in acute leukemias and pharmacological inhibition

**A** Western blot analysis of XIAP and cIAP-1 expression levels in primary tumor cells from nine individual ALL patients (see Table EV1 for patient characteristics) at diagnosis (ALL), complete remission (CR) and relapse (ALL-REL).

**B** XIAP protein expression in all ALL PDX samples (n=10) analysed in Figure 3B compared to PBMCs (n=2).

**C** The ARTS mimetic A4 decreases XIAP protein levels. LOUCY and NALM-6 cells were treated with 10µM A4 for the indicated time and XIAP protein levels were analyzed by western blot. Actin served as loading control. One representative immunoblot out of 3 independent experiments is shown.

**D** A4 synergistically induces apoptosis in combination with cytotoxic drugs. LOUCY and NALM-6 cells were treated with the indicated drugs or A4 alone or with a combination of both drugs for 48h. Apoptosis was quantified in AnnexinV / PI stained cells by flow cytometry. Mean ±SEM of 3 independent experiments with * $p < 0.05$, ** $p < 0.01$, *** $p < 0.001$, ****$p < 0.0001$ by unpaired Students t-test with Welch´s correction is shown.

Source data for Appendix and Expanded View are available online.

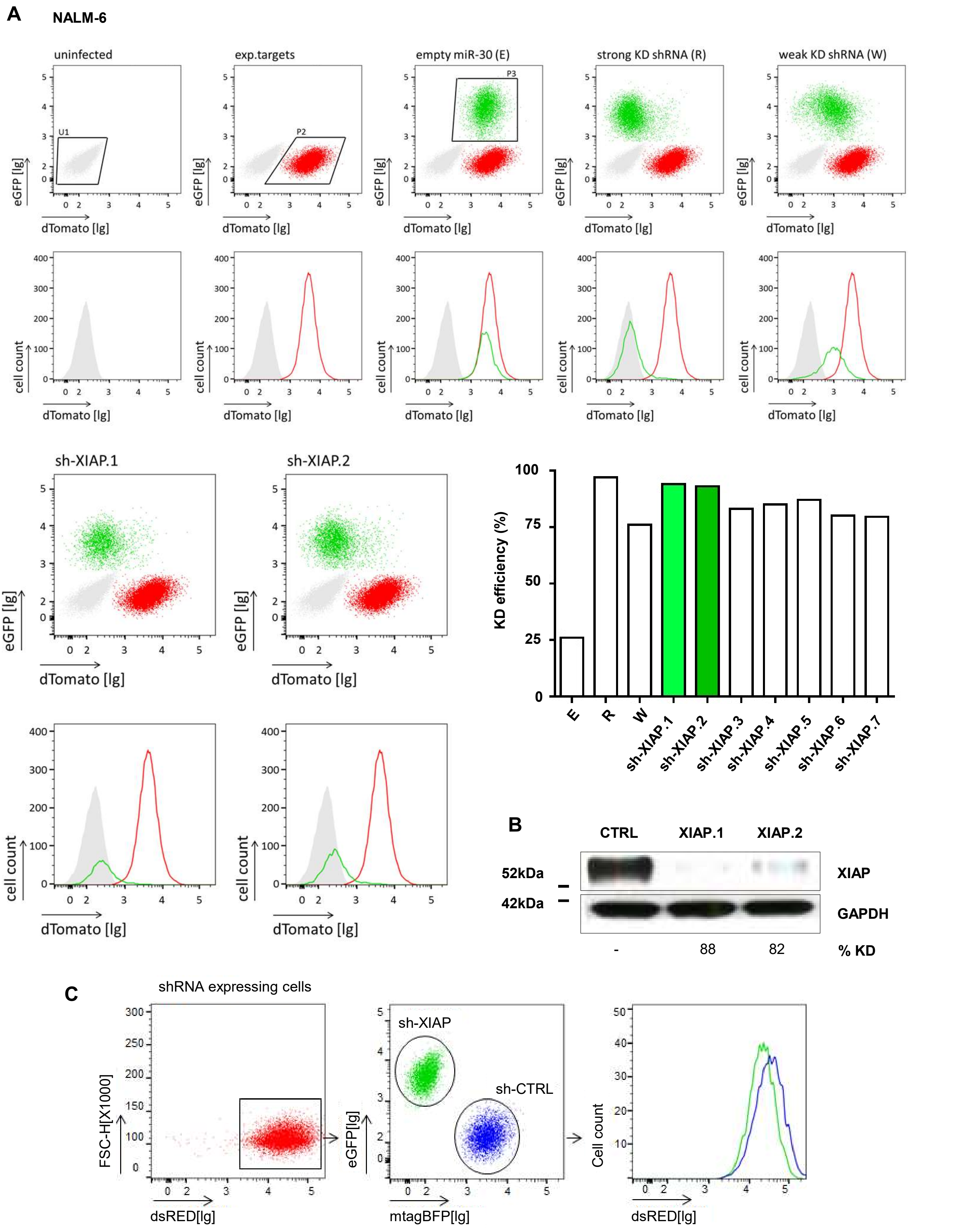


Appendix Figure S3

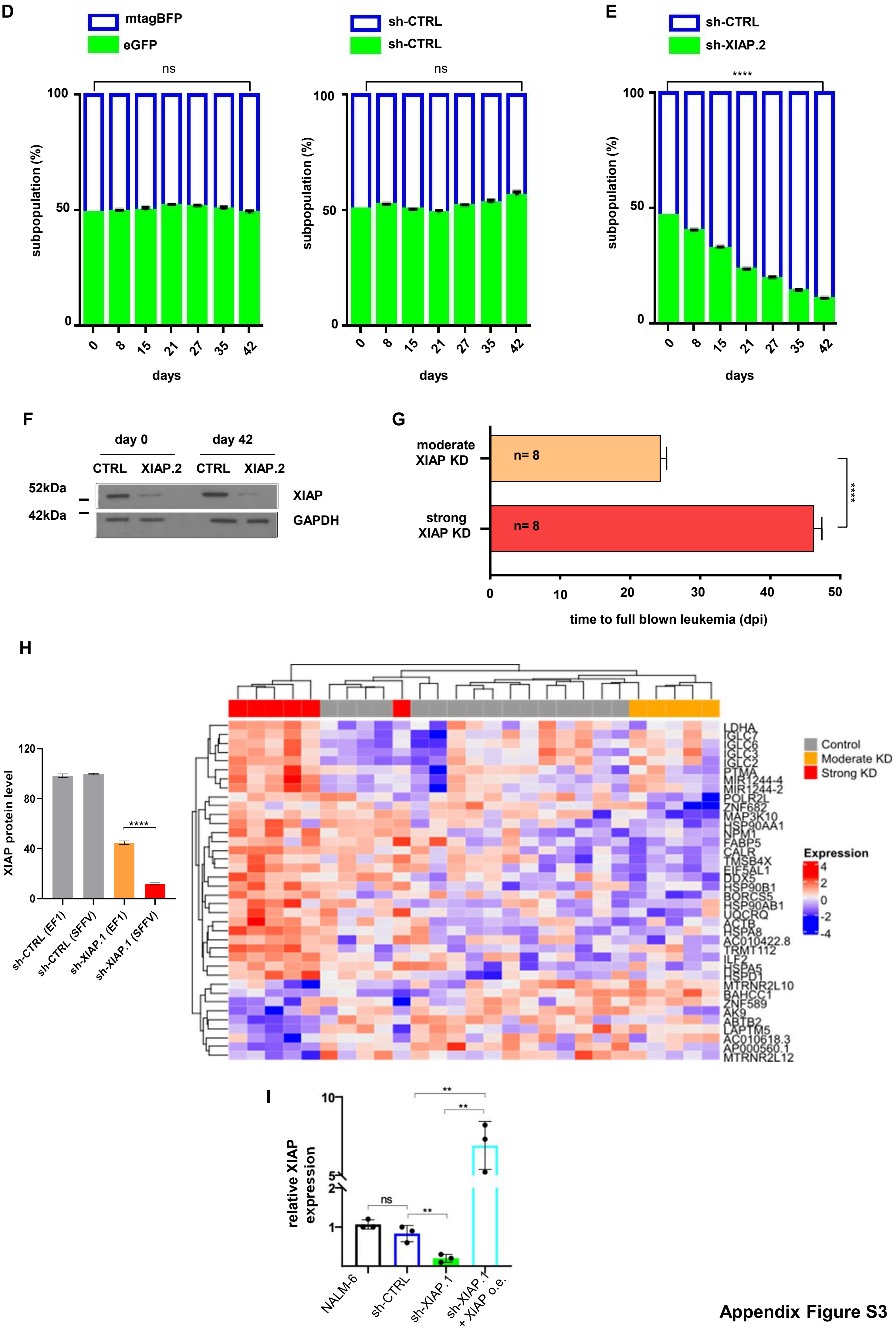


**Appendix Figure S3**

**Appendix Figure 3: Silencing XIAP sensitizes NALM-6 cells towards VCR *in vivo*.**
**A-B** Selection of efficient shRNA sequences targeting XIAP:
**A** The shRNA reporter system (Fellmann *et al,* 2013) enables to determine the efficiency of several shRNA sequences. Multiple genomic shRNA target sequences were cloned into the 3'UTR of a dTomato marker gene and constitutively expressed in NALM-6 cells (red). dTomato-positive NALM-6 cells were subsequently transduced with a second lentiviral vector expressing a specific mir30-shRNA sequence and eGFP as marker for transduction. Reduced expression of dTomato intensity in the GFP/dTomate double-positive population takes place only if the shRNA sequence/eGFP recognizes the specific target sequence located in the 3'UTR of dTomato. Note that the grey and red populations are repeated in all panels as references to compare the shift of the dTomato/eGFP double positive population. Representative flow cytometry plots of NALM-6 (untransduced, transduced with experimental targets, empty mir30, strong or weak shRNA) are shown. Quantification of KD efficiency is summarized and plotted as % of KD efficiency using shRenilla as CTRL. shXIAP.1 and shXIAP.2 sequences have been selected for further studies. E = empty control; R and W are positive controls for strong and weak KD, respectively.
**B** KD efficiency of selected XIAP KD sequences. sh-XIAP.1 and sh-XIAP.2 were selected for further studies. Western blot analysis in NALM-6 cells confirm efficient XIAP KD on protein level. GAPDH was used as loading control. One representative immunoblot out of 3 independent experiments is shown. **C**
**C** Gating strategy. To study the subpopulations' distribution of sh-CTRL/mTagBFP versus sh-XIAP/eGFP ALL cells, ds-RED cells were analyzed for expression of mTagBFP and eGFP. Back-gating allowed to compare dsRED expression between both subpopulations.
**D** Expression of transgenes and control shRNA sequences does not affect proliferation of NALM-6 *in vitro*. NALM-6 cells expressing either mTagBFP or eGFP alone (left panel) or in combination with sh-CTRL (right panel) were mixed at a 1:1 ratio and monitored by flow cytometry for up to 6 weeks. Relative proportion of each subpopulation is depicted. Mean ±SEM of 3 independent experiments is depicted; ns (not significant) by unpaired Students t-test.
**E** Strong KD of XIAP impairs NALM-6 *in vitro*. Relative proportion of NALM-6 cells expressing sh-CTRL/mTagBFP versus sh-XIAP.2/eGFP *in vitro* was measured by flow cytometry at the indicated time points. Mean ± SEM of 3 independent experiments, **** p <0.0001 by unpaired Students t-test.
**F** XIAP KD remained stable over time. Representative immunoblot indicating XIAP KD NALM-6 cell lines at the beginning (day 0) and at the end (day 42) of the *in vitro* experiments depicted in E. GAPDH was used as a loading control. One representative immunoblot out of 3 independent experiments is shown.
**G** Strong XIAP KD prolongs life span of leukemic mice. Summary of competitive *in vivo* assays performed with a 1:1 mixture of sh-CTRL/mTagBFP and sh-XIAP/eGFP KD NALM-6 cells with moderate (orange, n=8) or strong (red, n=8) XIAP KD. Mice of each group were sacrificed at first sign of leukemia. Shown is mean ± SEM, **** p <0.0001 by unpaired Students t-test.
**H** Gene expression analysis. NALM-6 control cells and NALM-6 cells with moderate and strong XIAP KD were subjected to RNA SCRB-seq analysis. XIAP KD was confirmed by protein immunoassay (Simple Western). Quantification of XIAP protein expression of 3 independent experiments is depicted; mean ± SEM, **** p <0.0001 by unpaired t-test. Heatmap of 38 genes differentially expressed between any two groups is shown (adjusted p-value ≤ 0.25). All gene expressions have been scaled to a mean value of 0 and a variance of 1.
**I** Quantification of XIAP protein expression in the different cell populations via Simple Western (Immunoassay). Data are expressed as mean ± SD (n=3). ns, not significant, ** p ≤0.01 by unpaired Student's t-test.

Source data for Appendix and Expanded View are available online.
