# Supplementary material for: X‐linked inhibitor of apoptosis protein represents a promising therapeutic target for relapsed/refractory ALL
Source: EMBO Mol Med. 2022 Nov 23;15(1):e14557. doi: 10.15252/emmm.202114557 (PMC9832863; doi:10.15252/emmm.202114557)
Supplement: Supplementary file 2 — Expanded View Figures PDF [file EMMM-15-e14557-s005.pdf]

Expanded View Figures

Figure EV1. Apoptosis induction by the SM-drug combination is independent from TNFα and RIPK1.

A–D LOUCY (A, B) or NALM-6 cells (C, D) cells were treated for 48 h with SM and/or VCR and/or TNFα in the presence of the TNFα-neutralizing antibody adalimumab (ADM). Apoptosis induction was determined by flow cytometry upon staining with Annexin-V/PI (A, C, D) and by analyzing active caspase-8 (B). Data are expressed as mean ± SD of three independent experiments; ***$P < 0.001$ by unpaired Student's *t*-test.

E Samples from two pediatric patients (ALL-3 and ALL-4; see Table EV1 for details) were treated for 24 h with DXR or VCR and/or LBW242 in combination with adalimumab. Apoptosis induction was determined by flow cytometry upon staining with Annexin-V/PI.

F, G Ripoptosome inhibition does not inhibit apoptosis by the SM-drug combination. NALM-6 cells were treated for 48 h with SM and VCR (E) or SM and TNFα (F) in the presence of the RIPK1-inhibitor Necrostatin-1 (Nec). Apoptosis induction was determined by flow cytometry upon staining with Annexin-V/PI. Data are expressed as mean ± SD of three independent experiments with *$P < 0.05$, **$P < 0.01$ by unpaired Student's *t*-test.

 © 2022 The Authors

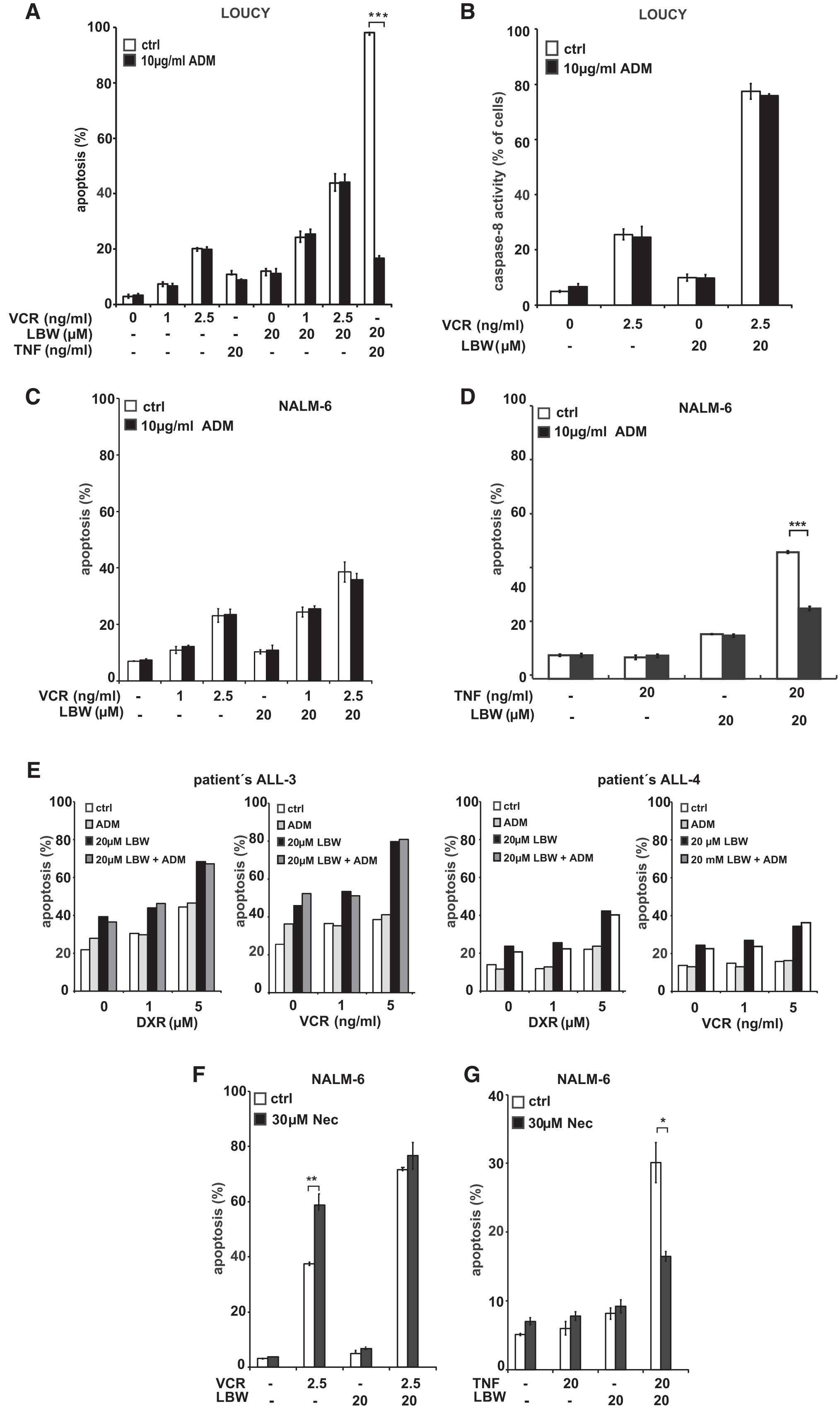
A
LOUCY
ctrl
10µg/ml ADM
***
apoptosis (%)
VCR (ng/ml) 0 1 2.5 - 0 1 2.5 -
LBW (µM) - - - - 20 20 20 20
TNF (ng/ml) - - - 20 - - - 20
B
LOUCY
ctrl
10µg/ml ADM
caspase-8 activity (% of cells)
VCR (ng/ml) 0 2.5 0 2.5
LBW (µM) - - 20 20
C
NALM-6
ctrl
10µg/ml ADM
apoptosis (%)
VCR (ng/ml) - 1 2.5 - 1 2.5
LBW (µM) - - - 20 20 20
D
NALM-6
ctrl
10µg/ml ADM
***
apoptosis (%)
TNF (ng/ml) - 20 - 20
LBW (µM) - - 20 20
E
patient´s ALL-3
patient´s ALL-4
ctrl
ADM
20µM LBW
20µM LBW + ADM
20 µM LBW
20 mM LBW + ADM
apoptosis (%)
DXR (µM)
VCR (ng/ml)
F
NALM-6
ctrl
30µM Nec
**
apoptosis (%)
VCR - 2.5 - 2.5
LBW - - 20 20
G
NALM-6
ctrl
30µM Nec
*
apoptosis (%)
TNF - 20 - 20
LBW - - 20 20

Figure EV1.

Figure EV2. IAP expression in acute leukemias.

A–E mRNA expression of XIAP (A) in different ALL subgroups and mRNA expression of cIAP-1 (B, C) and cIAP-2 (D, E) in samples shown in Fig 3A (B, D) and in distinct ALL subgroups (C, E). Subgroups of ALL significantly differed in their expression levels of all three genes with (A) XIAP: ANOVA $P = 3.74*10^{-6}$; (C) cIAP-1: ANOVA $P = 4.45*10^{-13}$ and (E) cIAP-2: ANOVA $P = 6.89*10^{-14}$. Comparing pairs of two subgroups, statistically significant differences were detected by *post hoc* analysis for a *P*-value < 0.05 (A) for XIAP in B-other compared to ETV6-t and Ph-positive as well as Burkitt compared to ETV6-t, Hyperdiploidy, Ph-like, Ph-positive and T-ALL, (C) for cIAP-1 in T-ALL compared to B-other, KMT2A-t, Ph-like and Ph-positive (E) for cIAP-2 in Burkitt compared to B-other, KMT2A-t, Ph-positive and TCF3-t as well as for T-ALL compared to B-other, KMT2A-t and Ph-positive and Ph-positive compared to KMT2A-t. Each dot represents one patient sample (biological replicates). Data are expressed as median, 25th and 75th percentile, whiskers indicate min/max; ns (not significant) by Games-Howell *post hoc* test.

© 2022 The Authors

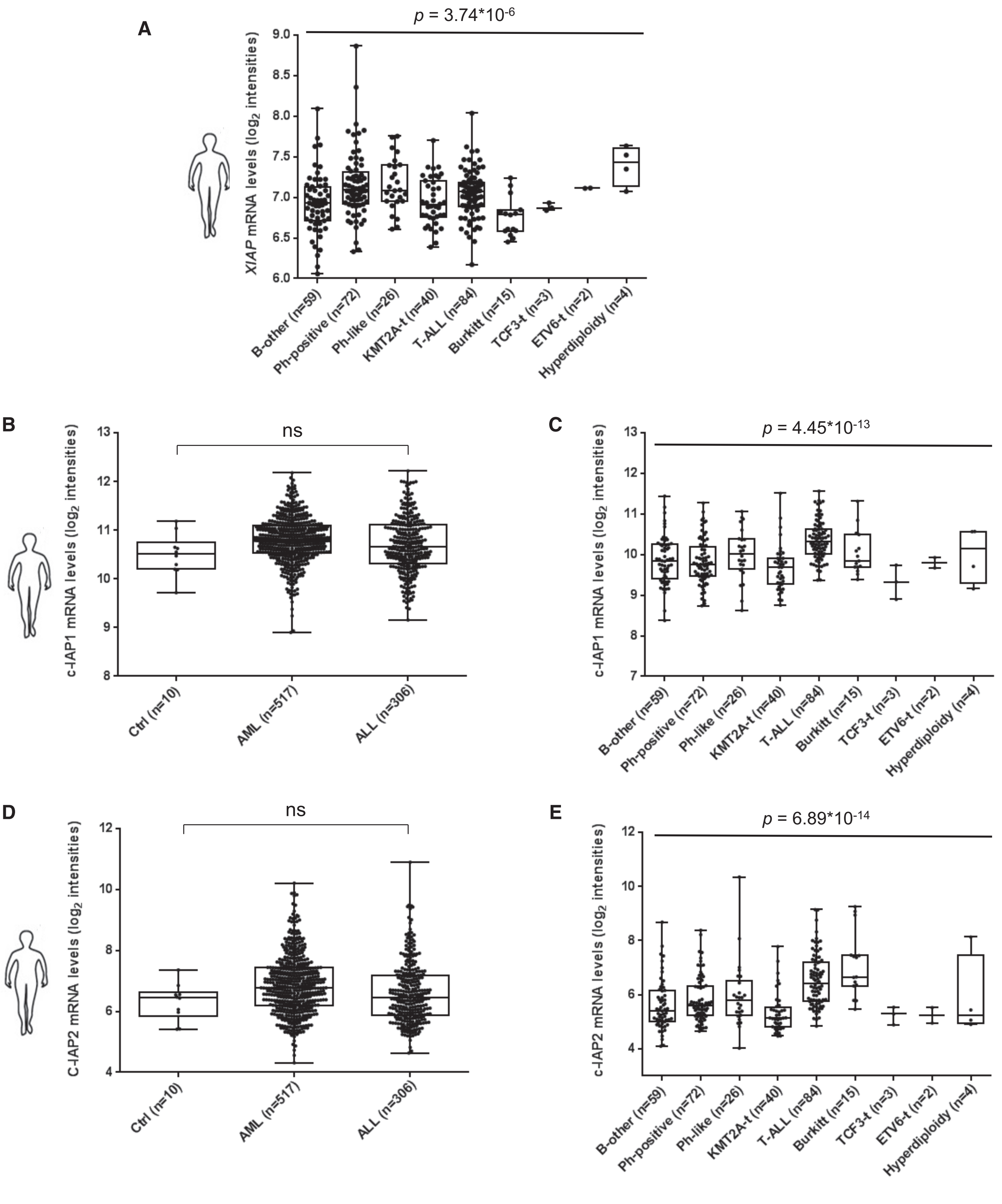
A
p = 3.74*10-6
XIAP mRNA levels (log2 intensities)
9.0
8.5
8.0
7.5
7.0
6.5
6.0
B-other (n=59)
Ph-positive (n=72)
Ph-like (n=26)
KMT2A-t (n=40)
T-ALL (n=84)
Burkitt (n=15)
TCF3-t (n=3)
ETV6-t (n=2)
Hyperdiploidy (n=4)
B
ns
c-IAP1 mRNA levels (log2 intensities)
13
12
11
10
9
8
Ctrl (n=10)
AML (n=517)
ALL (n=306)
C
p = 4.45*10-13
c-IAP1 mRNA levels (log2 intensities)
13
12
11
10
9
8
7
B-other (n=59)
Ph-positive (n=72)
Ph-like (n=26)
KMT2A-t (n=40)
T-ALL (n=84)
Burkitt (n=15)
TCF3-t (n=3)
ETV6-t (n=2)
Hyperdiploidy (n=4)
D
ns
C-IAP2 mRNA levels (log2 intensities)
12
10
8
6
4
Ctrl (n=10)
AML (n=517)
ALL (n=306)
E
p = 6.89*10-14
c-IAP2 mRNA levels (log2 intensities)
12
10
8
6
4
B-other (n=59)
Ph-positive (n=72)
Ph-like (n=26)
KMT2A-t (n=40)
T-ALL (n=84)
Burkitt (n=15)
TCF3-t (n=3)
ETV6-t (n=2)
Hyperdiploidy (n=4)

Figure EV2.

Figure EV3. XIAP plays an essential role for patients' r/r ALL cells growing in mice.

A Control and XIAP-targeting shRNAs are expressed in similar amounts in PDX cells. Experiments were performed as described in Fig 5A, and expression levels of dsRED in the sh-CTRL/mTagBFP and sh-XIAP/eGFP subpopulation were analyzed by FACS.

B Expression of transgenes and control shRNA sequences does not affect proliferation of PDX ALL cells *in vivo*. Competitive *in vivo* assays with different combinations of colors and control shRNA sequences for up to four passages in ALL-265 *in vivo*. Each mouse is considered as one independent experiment (P0, $n = 16$; P1, $n = 16$; P2, $n = 16$; P3, $n = 14$). Data are expressed as mean ± SEM, ns, not significant by unpaired Student's *t*-test.

C, D Experiments were performed and are depicted exactly as described in Fig 5C and D, except that PDX ALL-199 was studied. In (C) each mouse analyzed ($n = 8$) is considered as one independent experiment. Data are expressed as mean ± SEM of all mice analyzed; ****$P < 0.0001$ by unpaired Student's *t*-test. (D) ALL-199 PDX cells of a representative mouse were analyzed separately for dsRED/shRNA high- and low-expressing cells.

Source data are available online for this figure.

 © 2022 The Authors

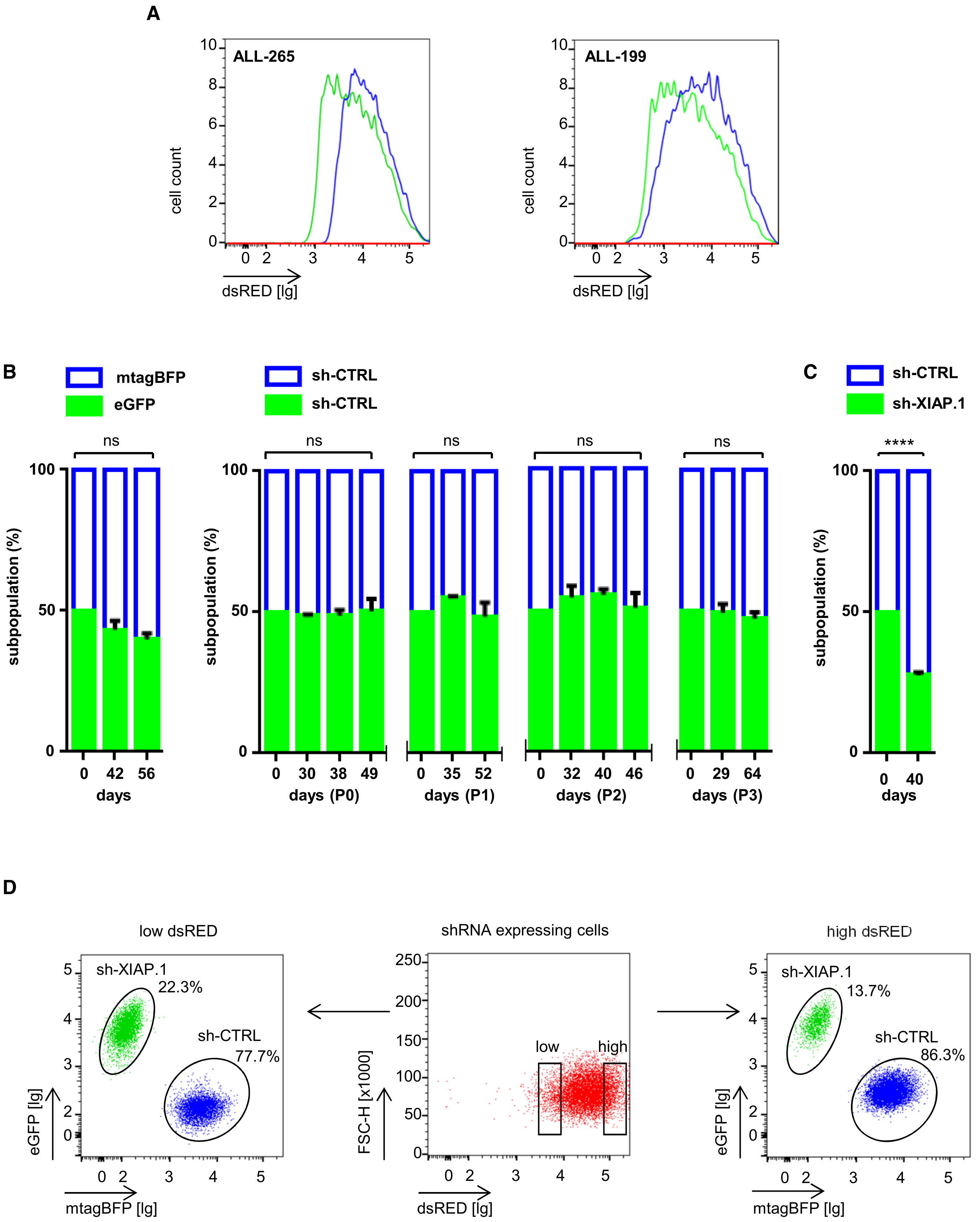
A
ALL-265
ALL-199
cell count
dsRED [lg]
B
mtagBFP
eGFP
sh-CTRL
sh-CTRL
ns
subpopulation (%)
days
days (P0)
days (P1)
days (P2)
days (P3)
C
sh-CTRL
sh-XIAP.1
****
D
low dsRED
shRNA expressing cells
high dsRED
sh-XIAP.1
22.3%
sh-CTRL
77.7%
low
high
13.7%
86.3%
eGFP [lg]
mtagBFP [lg]
FSC-H [x1000]
dsRED [lg]

Figure EV3.
