# Supplementary material for: X‐linked inhibitor of apoptosis protein represents a promising therapeutic target for relapsed/refractory ALL
Source: EMBO Mol Med. 2022 Nov 23;15(1):e14557. doi: 10.15252/emmm.202114557 (PMC9832863; doi:10.15252/emmm.202114557)
Supplement: Supplementary file 3 — Table EV1 [file EMMM-15-e14557-s002.pdf]

Table EV1. Clinical characteristics of ALL patient samples

sample	type	disease stage*	age [years]	sex	cytogenetics and gene fusions	mutations∞	Reference
**ALL-1**	ETP	ID	13	M	no BCR/ABL, no MLL/AF4, no TEL/AML1	N.D.	-
**ALL-2**	BCP	R	14	F	no BCR/ABL, no MLL/AF4, no TEL/AML1 t(9;14)	N.D.	-
**ALL-3**	ETP	ID	9	M	no BCR/ABL, no MLL/AF4, no TEL/AML1	N.D.	-
**ALL-4**	BCP	ID	1	F	TEL/AML1	N.D.	-
**ALL-5**	BCP	ID	2	M	TEL/AML1	N.D.	-
**ALL-6**	BCP	ID	1	F	no BCR/ABL, no MLL/AF4, no TEL/AML1	N.D.	-
**ALL-7**	BC-L	ID	20	M	N.D.	N.D.	-
**ALL-8**	DLBCL	CR	4	M	N.D.	N.D.	-
**ALL-9**	BCP	CR	3	M	no BCR/ABL, no MLL/AF4, no TEL/AML1	N.D.	-
**ALL-10**	BCP	CR	1	M	MLL del, AML1 gain, ABL gain	N.D.	-
**ALL-11**	BCP	R	8	M	no BCR/ABL, no MLL/AF4, no TEL/AML1	N.D.	-
**ALL-12**	BCP	R	2	F	no BCR/ABL, no MLL/AF4, no TEL/AML1	N.D.	-
**ALL-50**	BCP-ALL	ID	7	F	TCF3-PBX1; t(1;19)	N.D.	[1]

**ALL-177**	BCP-ALL	ID	8	F	ETV6-RUNX1; deletion 12p	N.D.	[1,2]
**ALL-199**	BCP-ALL	R2	8	F	somatic trisomy21; leukemic homozygous 9p deletion; P2RY8-CRLF2	N.D.	[1,2]
**ALL-230**	T-ALL	ID	4	M	t(11;14)(p32;q11); rearrangement of TAL1-gene with the T-cell receptor locus	NOTCH1	[2]
**ALL-233**	BCP-ALL	ID	<1	M	t(2;15)(p13;q15)	AKAP6, CSMD1, FAT1	[2]
**ALL-265**	BCP-ALL	R1	5	F	hyperdipoidy with additional 6,13,14,17,18,21,X chromosome	KMT2D, HERC1	[1,2]
**ALL-363**	BCP-ALL	ID	65	M	BCR-ABL p190; t(9;22)(q34,q11)	BCL11B, GRM3, IKZF1	[2]
**ALL-435**	BCP-ALL	ID	<1	M	MLL-ENL, t(11;19)	N.D.	[2]
**ALL-817**	BCP-ALL	ID	74	F	MLL-AF4; t(4;11)(q21;q23)	N.D.	-
**ALL-818**	BCP-ALL	ID	47	M	MLL-AF4; t(4;11)(q21;q23)	N.D.	-

*when the primary sample was obtained; ∞ mutations determined by panel sequencing; BCP-ALL = B-cell precursor ALL; T-ALL = T-cell ALL; ETP = early T-cell precursor; DLBCL= diffuse large B-cell lymphoma; BC-L = B-cell leukemia; ID = initial diagnosis; R1 = 1st relapse; R2 = 2nd relapse; F = female; M = male; N.D. not determined.

[1] Heckl et al., Leuk Lymphoma 2019
[2.] Ebinger et al., Cancer Cell 2016
