# Supplementary material for: X‐linked inhibitor of apoptosis protein represents a promising therapeutic target for relapsed/refractory ALL
Source: EMBO Mol Med. 2022 Nov 23;15(1):e14557. doi: 10.15252/emmm.202114557 (PMC9832863; doi:10.15252/emmm.202114557)
Supplement: Supplementary file 4 — Table EV2 [file EMMM-15-e14557-s012.pdf]

Table EV2. Gene set enrichment analysis

Genes enriched in control compared to high XIAP KD			
**Pathway name**	**Size**	**NES**	**p-value**
HALLMARK_TNFA_SIGNALING_VIA_NFKB	134	-1.26	0.066
GOBP_REGULATION_OF_T_CELL_APOPTOTIC_PROCESS	19	-1.60	0.018
GOBP_THYMOCYTE_APOPTOTIC_PROCESS	15	-1.57	0.018
GOBP_NEGATIVE_REGULATION_OF_LEUKOCYTE_APOPTOTIC_PROCESS	29	-1.54	0.015
GOBP_NEGATIVE_REGULATION_OF_INTRINSIC_APOPTOTIC_SIGNALING_PATHWAY_IN_RESPONSE_TO_DNA_DAMAGE	24	-1.4	0.075
GOBP_REGULATION_OF_INTRINSIC_APOPTOTIC_SIGNALING_PATHWAY_BY_P53_CLASS_MEDIATOR	20	-1.39	0.067
GOBP_REGULATION_OF_LEUKOCYTE_APOPTOTIC_PROCESS	49	-1.33	0.071

Genes enriched in low XIAP KD compared to high XIAP KD			
**Pathway name**	**Size**	**NES**	**p-value**
HALLMARK_TNFA_SIGNALING_VIA_NFKB	134	-1.26	0.066
GOBP_REGULATION_OF_T_CELL_APOPTOTIC_PROCESS	19	-1.60	0.018
GOBP_THYMOCYTE_APOPTOTIC_PROCESS	15	-1.57	0.018
GOBP_NEGATIVE_REGULATION_OF_LEUKOCYTE_APOPTOTIC_PROCESS	29	-1.54	0.015
GOBP_NEGATIVE_REGULATION_OF_INTRINSIC_APOPTOTIC_SIGNALING_PATHWAY_IN_RESPONSE_TO_DNA_DAMAGE	24	-1.4	0.075
GOBP_REGULATION_OF_INTRINSIC_APOPTOTIC_SIGNALING_PATHWAY_BY_P53_CLASS_MEDIATOR	20	-1.39	0.067
GOBP_REGULATION_OF_LEUKOCYTE_APOPTOTIC_PROCESS	49	-1.33	0.071

NES=normalized enrichment score
