# Supplementary material for: X‐linked inhibitor of apoptosis protein represents a promising therapeutic target for relapsed/refractory ALL
Source: EMBO Mol Med. 2022 Nov 23;15(1):e14557. doi: 10.15252/emmm.202114557 (PMC9832863; doi:10.15252/emmm.202114557)
Supplement: Supplementary file 6 — PDF+ [file EMMM-15-e14557-s008.pdf]

Article

SOURCE DATA | TRANSPARENT PROCESS | OPEN ACCESS

EMBO Molecular Medicine

X-linked inhibitor of apoptosis protein represents a promising therapeutic target for relapsed/refractory ALL

Michela Carlet[1,2,†], Karin Schmelz[3,4,†], Jenny Vergalli[1,†], Tobias Herold[1,5,6], Daniela Senft[1], Vindi Jurinovic[1,5,7], Thomas Hoffmann[8], Jutta Proba[3], Nina Weichert[3], Christian Junghanß[9], Mareike Roth[8], Georg Eschenburg[10], Malwine Barz[11], Günter Henze[3], Cornelia Eckert[3], Angelika Eggert[3], Johannes Zuber[8], Patrick Hundsdoerfer[3,12,13,‡] & Irmela Jeremias[1,6,7,*,‡]

Abstract

Acute lymphoblastic leukemia (ALL) represents the most frequent malignancy in children, and relapse/refractory (r/r) disease is difficult to treat, both in children and adults. In search for novel treatment options against r/r ALL, we studied inhibitor of apoptosis proteins (IAP) and Smac mimetics (SM). SM-sensitized r/r ALL cells towards conventional chemotherapy, even upon resistance against SM alone. The combination of SM and chemotherapy-induced cell death via caspases and PARP, but independent from cIAP-1/2, RIPK1, TNFα or NF-κB. Instead, XIAP was identified to mediate SM effects. Molecular manipulation of XIAP *in vivo* using microRNA-30 flanked shRNA expression in cell lines and patient-derived xenograft (PDX) models of r/r ALL mimicked SM effects and intermediate XIAP knockdown-sensitized r/r ALL cells towards chemotherapy-induced apoptosis. Interestingly, upon strong XIAP knockdown, PDX r/r ALL cells were outcompeted *in vivo*, even in the absence of chemotherapy. Our results indicate a yet unknown essential function of XIAP in r/r ALL and reveal XIAP as a promising therapeutic target for r/r ALL.

Keywords PDX; relapsed/refractory acute lymphoblastic leukemia; smac mimetics; therapeutic target; XIAP

Subject Categories Cancer; Haematology

DOI 10.15252/emmm.202114557 | Received 14 May 2021 | Revised 23 October 2022 | Accepted 28 October 2022 | Published online 23 November 2022

EMBO Mol Med (2023) 15: e14557

Introduction

Acute lymphoblastic leukemia (ALL) represents the single most frequent tumor in children and is difficult to treat, especially upon chemotherapy resistance and relapse, in both children and adults (Malard & Mohty, 2020). Novel treatment options are intensively needed to treat relapsed/refractory (r/r) ALL. Targeting inhibitor of apoptosis proteins (IAPs) in r/r ALL might be an attractive therapeutic concept as IAPs are often overexpressed and affect treatment outcome in multiple cancers, including ALL (LaCasse, 2013; Fulda, 2017).

The IAP family contains several members with strong anti-apoptotic activity, namely cellular IAP 1 and 2 (cIAP-1/2) and X-linked IAP (XIAP). Mechanistically, cIAP-1/2 mainly inhibits apoptosis downstream of tumor necrosis factor (TNF) by preventing the formation of pro-apoptotic signaling complexes. In contrast, XIAP functions downstream of both, intrinsic and extrinsic apoptosis pathways, by direct inhibition of caspases, downstream executers of apoptosis (Fulda, 2017). Given the prominent role of XIAP in cell death induction, extensive efforts led to the development of Smac

1 Research Unit Apoptosis in Hematopoietic Stem Cells, Helmholtz Zentrum München, German Center for Environmental Health (HMGU), Munich, Germany
2 Department of Biotechnology and Food Engineering, MCI, The Entrepreneur School, Innsbruck, Austria
3 Department of Pediatric Oncology/Hematology, Charité-Universitätsmedizin, Berlin, Germany
4 German Cancer Consortium (DKTK), Berlin, Germany
5 Laboratory for Leukemia Diagnostics, Department of Medicine III, University Hospital, LMU Munich, Munich, Germany
6 German Cancer Consortium (DKTK), Partnering Site Munich, Munich, Germany
7 Department of Pediatrics, Dr. von Hauner Children's Hospital, University Hospital, LMU, Munich, Germany
8 Research Institute of Molecular Pathology (IMP), Vienna, Austria
9 Department of Medicine, Clinic III – Hematology, Oncology, Palliative Medicine, Rostock University Medical Center, Rostock, Germany
10 Department of Pediatric Surgery, University Medical Center Hamburg-Eppendorf, Hamburg, Germany
11 University Children's Hospital Zurich, Zurich, Switzerland
12 Berlin Institute of Health, Berlin, Germany
13 Department of Pediatrics, Helios Klinikum Berlin-Buch, Berlin, Germany
*Corresponding author. Tel: +49 89 3187 1424; Fax: +49 89 3187 4225; E-mail: irmela.jeremias@helmholtz-muenchen.de
†These authors contributed equally to this work as first authors
‡These authors contributed equally to this work as senior authors

© 2022 The Authors. Published under the terms of the CC BY 4.0 license.

mimetics (SM), small molecules designed to inhibit the XIAP-caspase interaction. Comprehensive studies on the antitumor activity of these SM (Fulda & Vucic, 2012; Fulda, 2017; Morrish *et al*, 2020) demonstrated that, despite being developed as XIAP inhibitors, SM exert their antitumor activity by targeting cIAP-1/2 for ubiquitination and proteasomal degradation and subsequent NF-κB inactivation and TNFα-dependent apoptosis (Silke & Vince, 2017). Although solid tumors were the focus of numerous studies, acute leukemias were always included in trials, and several SM were effective against acute myeloid leukemia (AML) in preclinical studies, alone or in combination with other drugs (Brumatti *et al*, 2016; Lalaoui *et al*, 2016; Safferthal *et al*, 2017).

In ALL, studies on SM are restricted to preclinical studies, while clinical data are missing.

Distinct mono- and bivalent SM, such as Birinapant (TL32711; Condon *et al*, 2014) and BV6 (Varfolomeev *et al*, 2007), have been shown to induce cell death in B-ALL primary patient samples and patient-derived xenograft (PDX) cells in a RIPK1, TNFR1- and/or TNFR2-dependent manner (Fakler *et al*, 2009; Belz *et al*, 2014; McComb *et al*, 2016; Richmond *et al*, 2016; Schirmer *et al*, 2016; Aguadé-Gorgorió *et al*, 2020; Zinngrebe *et al*, 2020). While aggressive Ph-like ALL subtypes appeared to be enriched in responding samples, identification of biomarkers predicting SM response was challenging and expression levels of neither cIAP1/2, XIAP nor RIPK1 were shown to be predictive (McComb *et al*, 2016; Richmond *et al*, 2016; Aguadé-Gorgorió *et al*, 2020; Zinngrebe *et al*, 2020).

Combination therapies were mainly studied in SM-responsive samples where SM treatment synergized with conventional chemotherapy, which again depended on RIPK1 or TNFR1 signaling, at least in part (Servida *et al*, 2011; Loder *et al*, 2012; Richmond *et al*, 2016; Schirmer *et al*, 2016). SM enhanced caspase-dependent cell death in response to death receptor agonists (Fakler *et al*, 2009), increased response to glucocorticoids in a RIPK1-dependent manner (Belz *et al*, 2014) and synergized with hypomethylating agents to induce TNFα-dependent cell death (Gerges *et al*, 2016). However, the approach to combine SM with conventional chemotherapy in r/r ALL has not yet been studied.

Here, we evaluated the effect of targeting IAPs in r/r ALL using ALL cell lines as well as genetically engineered PDX ALL models *in vivo*. We identified XIAP, but not cIAP-1/2 as target for SM to augment the anti-tumor effect of chemotherapy. We detected a novel essential function of XIAP, indicating that XIAP represents an attractive therapeutic target for ALL, especially in the situation of treatment resistance or relapse.

Results

Smac mimetics sensitize r/r ALL towards cytotoxic drugs

In a first step, we assessed the sensitivity of B- and T-ALL cell lines to non-selective IAP inhibitors, including the multivalent SM LBW242 (or its orthologue LCL161; Sharma *et al*, 2006) and BV6 (Varfolomeev *et al*, 2007), which target several IAP family members, including cIAP-1/2 and XIAP. The majority of ALL cell lines displayed moderate response or resistance to SM-induced cell death, for which no apparent correlation with expression of either cIAP-1/2 nor XIAP was observed (Appendix Fig S1A–C). The two ALL cell lines (Molt-3 and Molt-4) were completely resistant towards LBW242 (Appendix Fig S1A) and were excluded from further studies. Four cell lines demonstrated a moderate resistance, and NALM-6 (B-ALL) and LOUCY (ETP-ALL) cells displayed strongly decreased sensitivity towards multiple SM (Appendix Fig S1A and C). We selected LOUCY cells for further analysis as they belong to the early immature ETP T-ALL subtype, known for its resistance against chemotherapeutic agents (Anderson *et al*, 2014); NALM-6 cells which showed similar response to SM than LOUCY were selected as a model for B-ALL disease. NALM-6 and LOUCY cells were additionally resistant against certain conventional cytotoxic drugs (Fig 1A and Appendix Fig S1D–F), serving as cell line models for r/r ALL.

Although various SM failed to induce strong cell death when applied as single agents (Appendix Fig S1A and C), apoptosis of ALL cell lines was significantly increased when SM were combined with vincristine (VCR; Fig 1A and Appendix Fig S1D–F), and a similar trend was observed in primary patient cells (Fig 1B and Table EV1 for clinical characteristics of patients). This effect was independent of the type of SM used and, within the range of concentration tested, the strongest effect was achieved with LCL161 and its orthologue LBW242 (Fig 1A and Appendix Fig S1D). In line with previous results (Loder *et al*, 2012; Belz *et al*, 2014; Richmond *et al*, 2016; Schirmer *et al*, 2016), SM also sensitized ALL cell lines to additional cytotoxic drugs of routine anti-leukemia treatment, including L-Asparaginase (L-ASP), Doxorubicin (DXR) and Glucocorticoids Prednisone (PRED) and Dexamethasone (DXM), with the exception that SM could not overcome resistance of LOUCY cells towards DXR (Appendix Fig S1E and F). Resistance of LOUCY cells to DXR might eventually be attributed to loss of function of p53, a known mediator of DXR-induced apoptosis (Prokocimer *et al*, 1998; Salih *et al*, 2020). Cell death by the combination of LBW242 and cytotoxic drugs (SM-drug combination) was mostly synergistic according to combination indices (Appendix Tables S1 and S2). Thus, in ALL cells resistant against apoptosis induction by SM alone, SM induced synergistic apoptosis when combined with cytotoxic drugs.

Smac mimetics enhance chemotherapy-induced apoptosis independently from TNFα, RIPK1 and NF-κB

Regarding the apoptosis signaling pathway activated by SM-drug combinations, we found downstream loss of mitochondrial membrane potential, activation of caspases-8, -9 and -3 and cleavage of PARP, indicating activation of the apoptotic pathway in both ALL cell lines analyzed (Fig 1C–F and Appendix Fig S1G–L).

In line with the findings that SM mainly targeted cIAP-1/2 and induced apoptosis dependent on TNFα signaling and/or formation of the ripoptosome (Petersen *et al*, 2007; Varfolomeev *et al*, 2007; Vince *et al*, 2007; Tenev *et al*, 2011; Loder *et al*, 2012; Belz *et al*, 2014), we have previously shown that cIAP-1/2 are important when SM-sensitized SM-sensitive REH ALL cells towards cytotoxic drugs (Loder *et al*, 2012). Here, in SM-resistant ALL cells, cIAP-1 was consistently degraded by the SM-drug combination (Appendix Fig S1M) but also by treatment with SM alone (Appendix Fig S1N), indicating that cIAP-1 degradation is not sufficient to induce cell death. While degradation of cIAP-2 was less consistent, no correlation of cIAP-2 degradation with cell death could be observed, further supporting that cIAP-1/2 degradation is not sufficient for cell death induction but may be a contributing event

 © 2022 The Authors

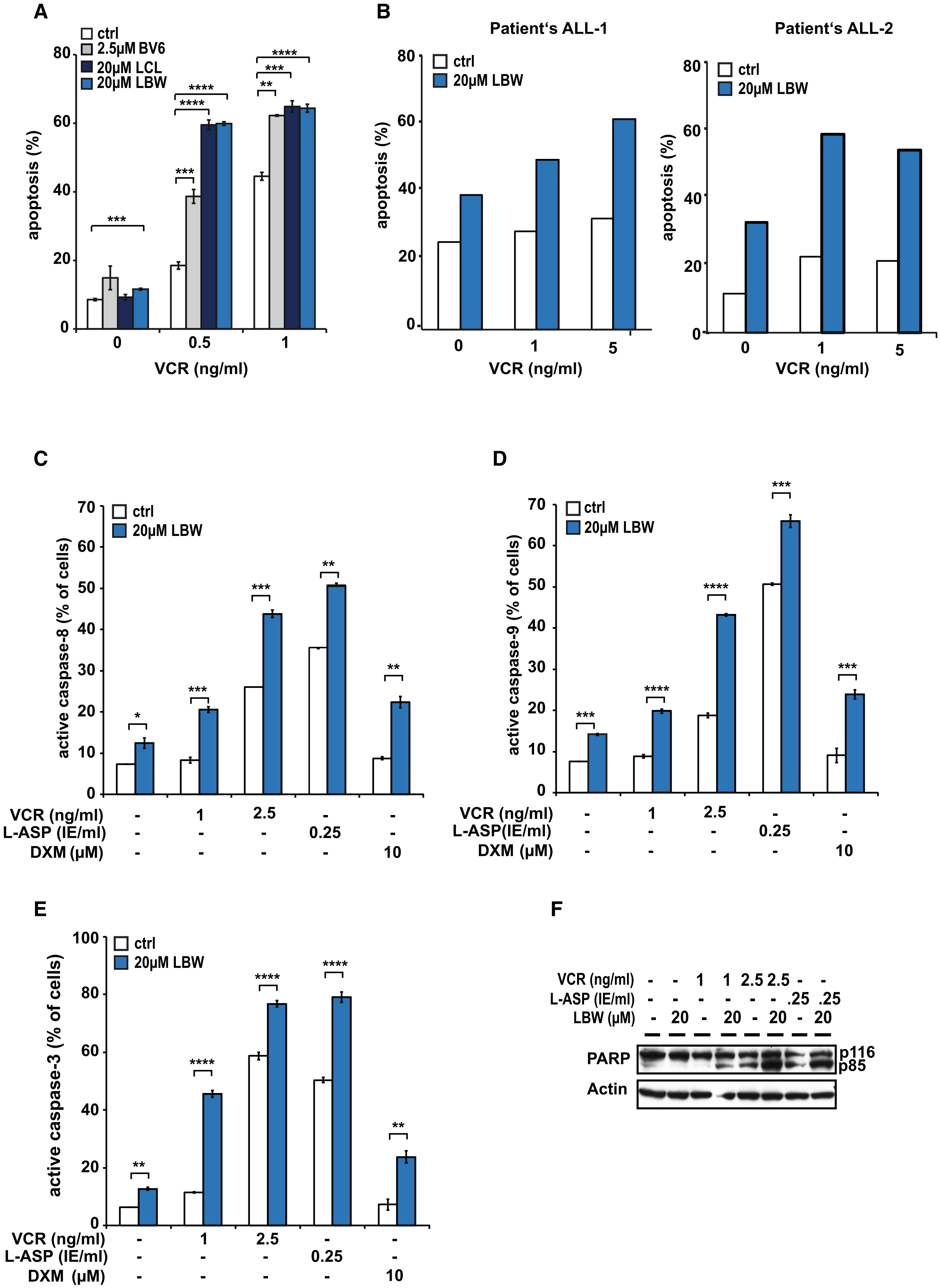
A
ctrl
2.5μM BV6
20μM LCL
20μM LBW
apoptosis (%)
VCR (ng/ml)
B
Patient's ALL-1
Patient's ALL-2
ctrl
20μM LBW
apoptosis (%)
VCR (ng/ml)
C
ctrl
20μM LBW
active caspase-8 (% of cells)
VCR (ng/ml)
L-ASP (IE/ml)
DXM (μM)
D
ctrl
20μM LBW
active caspase-9 (% of cells)
VCR (ng/ml)
L-ASP(IE/ml)
DXM (μM)
E
ctrl
20μM LBW
active caspase-3 (% of cells)
VCR (ng/ml)
L-ASP (IE/ml)
DXM (μM)
F
VCR (ng/ml)
L-ASP (IE/ml)
LBW (μM)
PARP
Actin
p116
p85

Figure 1.

◀ **Figure 1. Smac mimetics sensitize r/r ALL towards cytotoxic drugs.**

A LOUCY cells were treated *in vitro* with the indicated SMs with or without vincristine (VCR) for 48 h. Apoptosis was determined by flow cytometry upon staining with Annexin-V and Propidium Iodide (PI). Mean ± SD of three independent experiments is shown; $^{**}P < 0.01$, $^{***}P < 0.001$ and $^{****}P < 0.0001$ by unpaired Student's *t*-test.

B Fresh primary leukemic cells from two pediatric patients, one with common B-ALL at relapse (left) and one with T-ALL at diagnosis (right), both with poor response to second-line chemotherapy, were treated with LBW242 and VCR at the indicated concentrations. Apoptosis was measured after 24 h as described in (A).

C–F The SM-drug combination activates caspases and induces PARP cleavage. LOUCY cells were incubated with the indicated chemotherapeutic drugs with or without LBW242 for 48 h. Cells were stained with respective fluorescent substrates for caspase-8 (C) and -9 (D) or an antibody detecting the active caspase-3 subunit (E) and were subsequently analyzed by flow cytometry. Mean ± SD of three independent experiments is shown; $^{*}P < 0.05$, $^{**}P < 0.01$, $^{***}P < 0.001$ and $^{****}P < 0.0001$ by unpaired Student's *t*-test. Cleavage of PARP was measured by Western blot using Actin as loading control (F). One representative immunoblot out three independent experiments is shown.

Source data are available online for this figure.

(Fig 1A and Appendix Fig S1A, C, M and N). To further investigate signaling events initiated by SM treatment, we analyzed the role of TNFα in SM-induced cell death. To our surprise, inhibition of TNFα using the monoclonal antibody Adalimumab (Humira) did not reduce apoptosis or caspase-8 activation by the SM-drug combination in LOUCY (Fig EV1A and B), or apoptosis in NALM-6 (Fig EV1C and D) or primary ALL samples (Fig EV1E), while it efficiently blocked apoptosis induced by the TNFα plus LBW242 combination (Fig EV1A and D). These data suggest that in contrast to SM-sensitive cells (Loder *et al*, 2012; Richmond *et al*, 2016; Schirmer *et al*, 2016), apoptosis induction occurred independently from TNFα, similarly as we had described in neuroblastoma (Eschenburg *et al*, 2012).

SM-induced cIAP-1/2 degradation might induce apoptosis mediated by ripoptosome formation, a complex consisting of receptor-interacting protein kinase 1 (RIPK1), Fas-associated protein with death domain (FADD) and caspase-8 (Tenev *et al*, 2011; Belz *et al*, 2014). In the resistant ALL cell lines NALM-6 and LOUCY, the RIPK1-inhibitor Necrostatin-1 (Nec-1) was unable to inhibit apoptosis induction by SM in combination with VCR, while as a positive control, Nec-1 significantly reduced apoptosis initiated by TNFα plus LBW242 (Figs 2A and EV1E and F). Accordingly, apoptosis induction by the SM-VCR combination was not altered upon RIPK1 knockdown (KD), while the RIPK1 KD was effective in inhibiting apoptosis initiated by TNFα and LBW242 (Fig 2B). Thus, in contrast to SM-sensitive cells (Loder *et al*, 2012; Schirmer *et al*, 2016), the synergism between SM and chemotherapy is independent of RIPK1 in SM-resistant ALL cells. In SM-sensitive cells, SM-induced apoptosis is mediated by reducing pro-survival canonical, but increasing non-canonical NF-κB signaling (Bai *et al*, 2009; Gyrd-Hansen & Meier, 2010; Berger *et al*, 2011). Activation of the canonical NF-κB pathway requires signaling by NEMO (IKKγ) and is associated with phosphorylation and degradation of IκBα. Here, the SM-drug combination did not induce degradation of IκBα (Fig 2C) and apoptosis was not affected by KD of NEMO to levels that were shown to block NF-κB activation (Fig 2D; Konig *et al*, 2012; Elsarraj *et al*, 2017). Activation of non-canonical NF-κB pathway is known to be mediated by processing of the NF-κB2 subunit p100 to p52, which together with the NF-κB subunit RelB forms a transcriptionally active complex. Although SM alone or in combination with chemotherapy-induced p100 processing (Fig 2E), RelB KD had no effect on apoptosis induced by SM alone or in combination with chemotherapy (Fig 2F), indicating that non-canonical NF-κB signaling is dispensable for cell death induction.

In summary, our data indicate that SM in combination with chemotherapy-induced cell death in r/r ALL independently of cIAP-1/2 degradation as well as RIPK1, NF-κB and TNFα signaling, indicating that other mechanisms are at play as compared to SM-sensitive ALL cells (Loder *et al*, 2012).

XIAP is upregulated in primary tumor cells from ALL patients and targeting XIAP affects cell death by SM or VCR

We hypothesized that in r/r ALL, XIAP may serve as the major mediator of SM-induced effects. To evaluate a putative role of XIAP in r/r ALL, we first performed expression analysis of major IAPs in ALL. We had previously shown that XIAP protein is upregulated in primary tumor cells from children with T-ALL associated with poor prognosis (Hundsdoerfer *et al*, 2010) and now studied the expression of XIAP on mRNA and protein level in both, children and adults with ALL. In primary cells of a cohort of 833 adults, array data showed that *XIAP* mRNA was upregulated in ALL compared to healthy controls and expressed to a higher level than in AML (Figs 3A and EV2A), while *c-IAP1/2* mRNAs were not significantly increased in ALL (Fig EV2B–E).

XIAP protein was expressed in primary leukemic cells of ALL patients, where high XIAP protein levels were found in diagnosis and relapse samples, but not in remission controls (Appendix Fig S2A). Significant upregulation of XIAP protein was further detected in 7/7 PDX samples derived from children with ALL as well as in 3/3 PDX adult samples compared to controls (Fig 3B and Appendix Fig S2B; patient characteristics are summarized in Table EV1). Taken together, upregulation of XIAP protein represents a common feature for childhood and adult ALL.

To support our hypothesis of a putative functional relevance of XIAP in r/r ALL, we also tested the ARTS mimetic A4, which was reported to have a high specificity towards XIAP but not c-IAP1 (Mamriev *et al*, 2020). Treatment with A4 decreased XIAP levels in NALM-6 and LOUCY cells (Appendix Fig S2C) and equally sensitized cells to cytotoxic drugs (Appendix Fig S2D), again supporting the notion that XIAP might mediate SM effects. A role for XIAP is further supported by overexpression of XIAP in SM-resistant (NALM-6) and also in SM-sensitive (REH) B-ALL cell lines. Cell death induction by VCR or SM was reduced or nearly abrogated upon overexpression of XIAP, indicating that XIAP inhibited drug- as well as SM-induced cell death signaling (Fig 3C and D). XIAP overexpression showed similar effects on SM-sensitive and SM-resistant ALL cell lines; thus, increased XIAP levels abrogate cell death induced by the SM-drug combination in ALL cells, independently from their sensitivity towards SM. Taken together, XIAP

 © 2022 The Authors

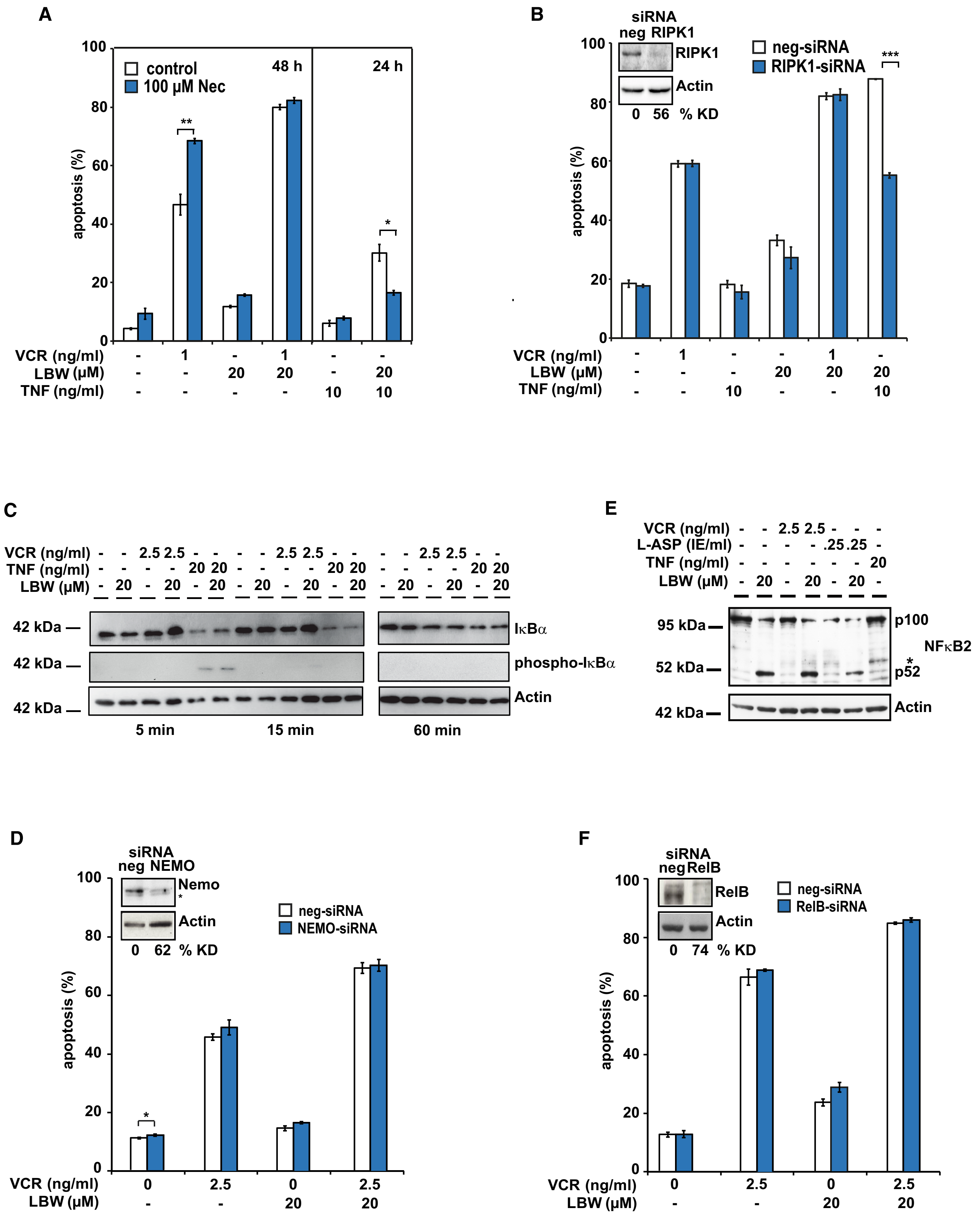
A
control
100 µM Nec
48 h
24 h
apoptosis (%)
VCR (ng/ml)
LBW (µM)
TNF (ng/ml)
B
siRNA
neg RIPK1
RIPK1
Actin
0 56 % KD
neg-siRNA
RIPK1-siRNA
apoptosis (%)
VCR (ng/ml)
LBW (µM)
TNF (ng/ml)
C
VCR (ng/ml)
TNF (ng/ml)
LBW (µM)
42 kDa
IκBα
phospho-IκBα
Actin
5 min
15 min
60 min
E
VCR (ng/ml)
L-ASP (IE/ml)
TNF (ng/ml)
LBW (µM)
95 kDa
52 kDa
42 kDa
p100
NFκB2
p52
Actin
D
siRNA
neg NEMO
Nemo
Actin
0 62 % KD
neg-siRNA
NEMO-siRNA
apoptosis (%)
VCR (ng/ml)
LBW (µM)
F
siRNA
neg RelB
RelB
Actin
0 74 % KD
neg-siRNA
RelB-siRNA
apoptosis (%)
VCR (ng/ml)
LBW (µM)

Figure 2.

◀

Figure 2. Apoptosis induction is independent from TNFα, RIPK1 and NF-κB.

A Necrostatin-1 (Nec) does not affect LBW-mediated sensitization to VCR-induced apoptosis. Apoptosis induction in LOUCY cells was determined by flow cytometry 48 h after treatment with SM and/or VCR (left) and/or TNFα (right) in the presence or absence of the RIPK1-inhibitor Nec. Mean ± SD of three independent experiments is shown; *$P < 0.05$ and **$P < 0.01$ by unpaired Student's *t*-test.

B RIPK1 is dispensable for apoptosis induction by LBW242 and VCR. LOUCY cells were transfected with a non-targeting siRNA (neg-siRNA) or a RIPK1-specific siRNA. Six hours post-transfection, cells were treated with the indicated drugs and apoptosis was measured 40 h thereafter. Mean ± SD of three independent experiments is shown; ***$P < 0.001$ by unpaired Student's *t*-test. Knockdown (KD) was verified by Western blot at the time of apoptosis measurement and KD efficiency was calculated compared to the control. One representative immunoblot out of three biological replicates is shown.

C The SM-drug combination does not activate the canonical NF-κB pathway. Phosphorylation and degradation of IκBα upon treatment with VCR or TNFα and/or LBW242 was determined in LOUCY cells by Western blot following treatment with the indicated drugs for 5, 15 or 60 min using Actin as loading control. One representative immunoblot out of three independent biological replicates is shown.

D KD of NEMO does not affect the SM-mediated sensitization to chemotherapy-induced apoptosis. Experiment was performed as described in (B), except that an siRNA targeting NEMO was used. Mean ± SD of three independent experiments is shown; *$P < 0.05$ by unpaired Student's *t*-test.

E The SM-drug combination activates the non-canonical NF-κB pathway. Cleavage of p100 NF-κB2 to the p52 fragment was determined by Western blot 24 h after treatment of LOUCY cells with chemotherapy or TNFα in combination with SM. Asterisk indicates an unspecific signal. One representative immunoblot out of three biological replicates is shown.

F KD of RelB does not inhibit apoptosis. Experiment was performed as described in (B), except that an siRNA targeting RelB was used. Mean ± SD of three independent experiments is shown; differences are not significant, by unpaired Student's *t*-test.

Source data are available online for this figure.

might be a critical regulator of apoptosis mediated by SM in combination with cytostatic drugs in ALL.

Silencing XIAP sensitizes ALL cells towards vincristine *in vivo*

To validate that SM-mediated inhibition of XIAP-sensitized-resistant ALL cells towards cytotoxic drugs, we mimicked XIAP inhibition on a molecular level by performing KD experiments.

Towards this aim, a short hairpin RNA (shRNA) cassette was expressed in the background of microRNA 30 (miR30; Stegmeier *et al*, 2005; Fig 4A); the miR30 approach enabled expressing the shRNA directly coupled to expression of a fluorochrome marker so that both, marker and shRNA, are expressed in equal molar amounts under control of the same Pol II promoter. This allows dose–response analysis within the same population by comparing cells with high marker/shRNA expression to those with low marker/shRNA expression. To perform competitive *in vivo* assays, control and XIAP KD populations were additionally molecularly marked using two different fluorochromes, mTagBFP and eGFP, respectively (Fig 4A). As important quality control, dsRED expression levels were highly similar between the two KD constructs, control shRNA and the shRNA targeting XIAP (Fig 4B), reassuring similar shRNA expression levels. The shRNA sequences used to target XIAP were selected using a reporter system (Fellmann *et al*, 2013; Appendix Fig S3A and B). The XIAP shRNAs were specific and induced stable KD of up to 98% when expressed under the strong viral SFFV promoter, while levels of cIAP-1 and cIAP-2 remained unchanged (Fig 4C and Appendix Fig S3B).

To mirror the effect of the SM-drug combination on a molecular level, we generated a derivative NALM-6 cell line with partial, but stable KD of XIAP, using the moderate eukaryotic elongation factor 1 alpha (EF1α) promoter (Fig 4D). Competitive *in vivo* growth assays were performed; a 1:1 mixture of CTRL and XIAP KD cells was injected into mice and their relative proportion was followed up over time, using flow cytometric measurement of the recombinant fluorochromes (Fig 4E and Appendix Fig S3C). In abundant control experiments, the system was proven to be devoid of bias regarding colors or control shRNA sequences, in cell lines *in vitro* (Appendix Fig S3D).

Using this approach, we found that partial inhibition of XIAP did not affect spontaneous growth of NALM-6 cells *in vivo* (Fig 4F). In contrast, when NALM-6 cells were xenografted into mice and mice systemically treated with VCR, the population with XIAP KD showed strongly reduced survival compared to controls, indicating that silencing XIAP-sensitized NALM-6 cells towards VCR-induced cell death (Fig 4F). Thus, partial KD of XIAP mimicked the effect of SM and sensitized SM-resistant ALL cells towards VCR *in vivo*.

To our surprise, we observed that NALM-6 cells with strong XIAP KD, using the strong SFFV promoter and two distinct shRNA sequences, spontaneously died over time *in vitro* and *in vivo*, indicating a yet unknown, genuine essential function of XIAP alone in r/r ALL (Fig 4G and Appendix Fig S3E and F). Survival time of mice injected with a 1:1 mixture of control and strong XIAP KD cells nearly doubled when compared with mice injected with a moderate XIAP KD population in their mixture; time to full-blown leukemia increased from 25 to 46 days (Appendix Fig S3G). We performed transcriptome analysis in which 38 genes were differentially expressed between cells with strong XIAP KD and sh-CTRL; regarding these genes, cells with moderate XIAP KD clustered together with sh-CTRL cells (Appendix Fig S3H). Cells with strong XIAP KD differed from cells with moderate XIAP KD and sh-CTRL regarding several GO-terms on apoptosis signaling, although at borderline significance ($P < 0.1$, Table EV2), pinpointing towards apoptosis as underlying mechanism for cell death by strong XIAP KD. In agreement, increased PARP cleavage was detected in NALM-6 cells with strong XIAP KD compared to sh-CTRL (Fig 4H). These effects were unequivocally dependent on XIAP, as XIAP re-constitution in shXIAP NALM-6 cells *in vitro* rescued the cells from cell death (Fig 4I and Appendix Fig S3I). As such a function might have an important impact for using XIAP as putative therapeutic target, we asked whether inhibiting XIAP alone might be effective in the absence of chemotherapy.

XIAP plays an essential role for patients' r/r ALL cells growing in mice

Aiming for a model which closely mimics the clinical situation, we worked with patients' ALL cells and in the complex environment of individual patients' tumors. All experiments were performed *in vivo*, using the individualized xenograft mouse model (Terziyska *et al*, 2012) and samples of two pediatric patients with r/r ALL.

 © 2022 The Authors

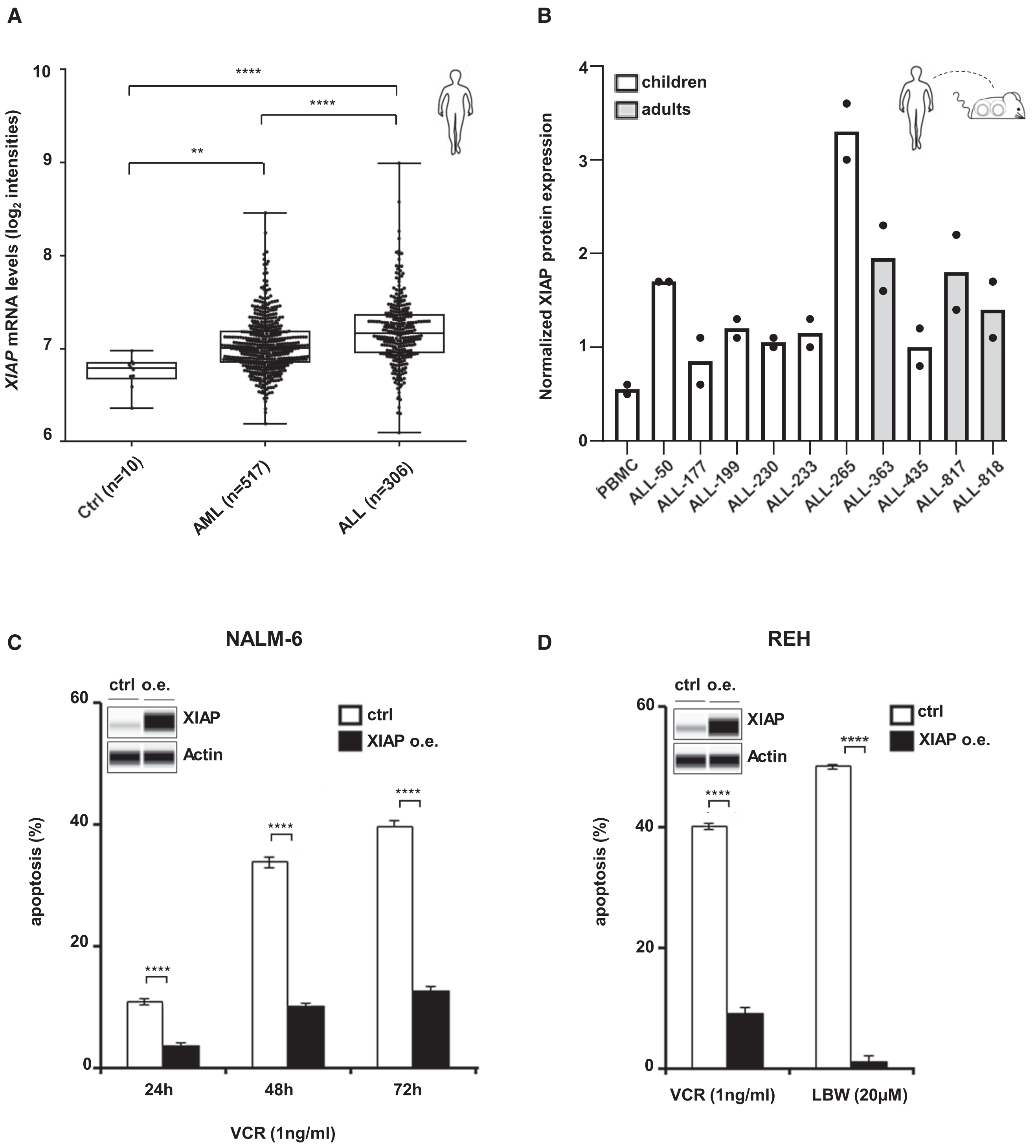

Figure 3. XIAP is upregulated in primary ALL patient samples and overexpression regulates cell death induction.

A Expression of XIAP mRNA in samples obtained from BM of healthy individuals (Ctrl, $n = 10$) or patients with AML ($n = 517$) or ALL ($n = 306$). Data are expressed as median, 25th and 75th percentile, whiskers indicate min/max; ** $P < 0.01$; **** $P < 0.0001$ by Games-Howell *post hoc* test.

B Protein levels of XIAP normalized to loading control in PDX ALL samples from pediatric and adult patients, analyzed by protein immunoassay (Simple Western) and depicted as mean ($n = 2$, biological replicates); PBMC, peripheral blood mononuclear cells (see Appendix Fig S2B).

C, D Parental and XIAP over-expressing NALM-6 cells (C) or REH cells (D) were treated with VCR or LBW242; apoptosis (Annexin-V/PI) was measured at the indicated time points (C) or after 72 h (D) by flow cytometry. Mean ± SEM of three independent experiments is shown; **** $P \leq 0.0001$ by unpaired Student's *t*-test. XIAP protein level was analyzed in parental and XIAP-overexpressing NALM-6 cells by protein immunoassay (Simple Western); Actin was used as loading control. One representative immunoassay out of three independent experiments is shown.

Source data are available online for this figure.

© 2022 The Authors

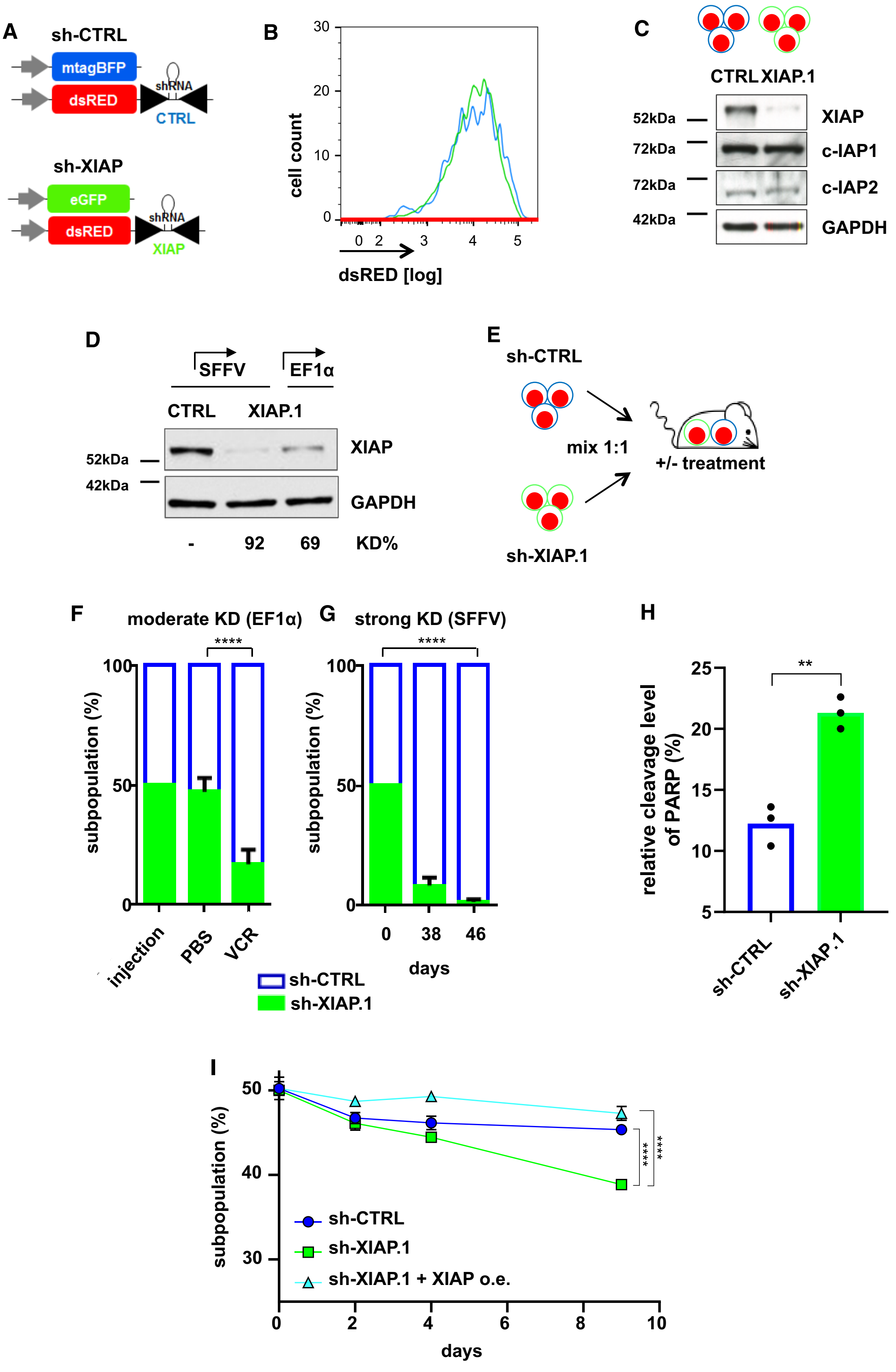

Figure 4.

© 2022 The Authors

◀ **Figure 4. Silencing XIAP sensitizes ALL cells towards Vincristine *in vivo*.**

A Schematic representation of lentiviral vectors. Control (CTRL) and XIAP-targeting shRNAs were expressed in the 3′untranslated region of dsRED in the background of a miR30 cassette under control of the Pol II promoter. mTagBFP and eGFP were used to mark each cell population; the control shRNA expressing plasmid was transduced into mTagBFP$^+$, the XIAP KD plasmid into eGFP$^+$ cells.

B shRNA expression control. Equal expression levels of ds-RED in the sh-CTRL/mTagBFP and sh-XIAP/eGFP subpopulation indicate identical expression levels of control and XIAP shRNA. One representative histogram out of three independent experiments is shown.

C Expression of IAP. Western blot analysis of XIAP in NALM-6 cells expressing sh-CTRL/mTagBFP or sh-XIAP/eGFP under the SFFV promoter; GAPDH was used as loading control. One representative immunoblot out of three independent experiments is shown.

D Western blot analysis of NALM-6 cells with moderate (69%, EF1α promoter) or strong (92%, SFFV promoter) KD of XIAP; GAPDH was used as loading control. The XIAP KD levels (% KD) were quantified relative to sh-CTRL. One representative immunoblot out of three independent experiments is shown.

E Schematic representation of competitive *in vivo* assays. Cells expressing sh-CTRL/mTagBFP or sh-XIAP/eGFP were mixed at a 1:1 ratio and injected into mice (±*in vivo* treatment); at indicated time points, mice were sacrificed, cells isolated from bone marrow and analyzed.

F Moderate knockdown of XIAP sensitizes towards chemotherapy. NALM-6 cells expressing sh-CTRL/mTagBFP or sh-XIAP/eGFP were mixed at a 1:1 ratio and injected into groups of mice. Four days after injection, mice were either treated with PBS ($n = 4$), or with Vincristine (VCR, $n = 8$), 0.3 mg/kg i.v. once a week for 3 consecutive weeks. Seven days after last treatment, mice were sacrificed, and relative proportion of sh-CTRL and sh-XIAP subpopulations re-isolated from the BM was quantified by flow cytometry. Data are depicted as mean ± SEM of all mice analyzed with ****$P \leq 0.0001$ by unpaired Student's *t*-test with Welch's correction.

G Strong knockdown of XIAP impairs NALM-6 growth *in vivo*. NALM-6 cells expressing sh-CTRL/mTagBFP or sh-XIAP/eGFP were mixed at a 1:1 ratio and injected into groups of mice ($n = 8$). Thirty-eight or 46 days after injection, mice were sacrificed, and relative proportion of sh-CTRL and sh-XIAP subpopulations re-isolated from the BM was quantified by flow cytometry. Data are depicted as mean ± SEM of all mice analyzed with ****$P \leq 0.0001$ by unpaired Student's *t*-test.

H PARP cleavage in NALM-6 cells with strong XIAP inhibition. NALM-6 cells were lentivirally transduced with sh-CTRL or sh-XIAP (SFFV). Four days later, cells were lysed and relative cleavage level of PARP (%) was analyzed by protein immunoassay (Simple Western). Mean ± SD of three independent experiments is shown; **$P < 0.01$ by unpaired Student's *t*-test.

I XIAP reconstitution in NALM-6 cells. NALM-6 cells were transduced with shCTRL, shXIAP.1 alone or shXIAP.1 together with the XIAP overexpression vector. At day 0, transduced cells were mixed in a 1:1 ratio with parental (untransduced) NALM-6 cells. Distribution of the fluorochrome marker was used as a readout to determine growth variations of the respective subpopulation over time. Mean ± SD of three independent experiments is shown; **$P < 0.01$ by unpaired Student's *t*-test.

Source data are available online for this figure.

For this analysis, we chose the PDX sample with highest XIAP expression, ALL-265 (Fig 5) and ALL-199 (Fig EV3) characterized by intermediate XIAP protein level (Fig 3B). Both children suffered from relapsed ALL (Table EV1), which was refractory to conventional treatment so that both succumbed to their disease early after diagnosis of their relapses.

Using lentiviruses (Terziyska *et al*, 2012), PDX ALL cells were molecularly modified identically as NALM-6 cells (Fig 5A), and dsRED expression was analyzed to verify similar shRNA expression in sh-CTRL and sh-XIAP cells (Fig EV3A); after each transduction, transgenic PDX r/r ALL cells were enriched by flow cytometry and re-amplified in mice (Fig 5A). As for NALM-6, these cells were used for competitive *in vivo* transplantation trials (Fig 5B).

Using the SFFV promoter, a strong KD of XIAP was achieved in PDX ALL cells, which remained stable for several passages (Fig 5B). While transgene and sh-CTRL expression had no impact on PDX proliferation *in vivo* (Fig EV3B), silencing XIAP induced a clear growth disadvantage in the absence of chemotherapy in both ALL-265 and ALL-199 (Figs 5C and EV3C). The XIAP KD population was severely diminished over time, while control cells increased.

To reassure that the effect was due to XIAP KD, a dose–response analysis was performed within the same animal by separately analyzing PDX ALL cells with high dsRED expression indicating strong XIAP KD and PDX ALL cells with low dsRED expression indicating moderate XIAP KD. PDX ALL cells with high dsRED expression showed the strongest effect and almost completely vanished (Figs 5D and EV3D), strengthening that XIAP KD was the underlying cause for subpopulation-specific disadvantage. Our data show that XIAP is required for growth of PDX r/r ALL samples in mice as silencing of XIAP decreased tumor load in a dose-dependent manner.

Taken together, our data reveal a yet unknown essential function for XIAP in patients ALL cells growing *in vivo*. While partial inhibition of XIAP by moderate KD mimicked the effect of SM and sensitized ALL cells towards chemotherapy (Figs 1–4), strong inhibition of XIAP reduced survival and proliferation of r/r ALL *in vivo* (Figs 4G and 5).

Discussion

In search for novel treatment options against r/r ALL, we discovered a yet unrecognized essential function of XIAP in r/r PDX ALL cells *in vivo*. Strong inhibition of XIAP induced major growth disadvantage and cell death, and moderate XIAP KD or SM-sensitized r/r ALL cells towards conventional chemotherapy. Our data show that XIAP represents an attractive therapeutic target, which might enable eliminating r/r ALL cells that are otherwise resistant against treatment.

To study the role of XIAP, we advanced preclinical PDX models and used genetic engineering to generate what we propose calling genetically-engineered PDX (GEPDX) models in parallel to genetically engineered mouse models (GEMM; Heckl *et al*, 2019). GEPDX models with the novel miR30-based KD approach combine the advantages of (i) use of patients' individual tumor cells in (ii) orthotopic mouse models *in vivo* with (iii) strong stable KD (iv) dose–response analyses within single animals, (v) convenient assay readout by flow cytometry and (vi) competitive *in vivo* assays bypassing inter-mouse deviations. Competitive *in vivo* approaches were frequently used in mouse studies on normal hematopoiesis and were adapted to leukemia and PDX models to spare resources and benefit from high sensitivity and reliability (Eaves, 2015; Maetzig *et al*, 2017; Liu *et al*, 2020; Carlet *et al*, 2021). While the high clinical relevance of the PDX model is widely accepted (Gao *et al*, 2015; Guo *et al*, 2016; Townsend *et al*, 2016; Izumchenko *et al*, 2017; Evrard, Srivastava *et al*, 2020), additional studies, including non-competitive *in vivo* experiments and survival analyses will be suitable to confirm the translational

© 2022 The Authors

relevance of the effects described here. In addition, the absence of a functional immune system remains a potential limitation (Byrne *et al*, 2017). Therefore, a possible influence of the immune system on the effects of XIAP KD in ALL PDX remains to be determined; as apoptotic cells express "eat me" signals capable to trigger an immune response (Hochreiter-Hufford & Ravichandran, 2013), effects of XIAP KD may even be increased.

XIAP is known to act as a direct caspase inhibitor, inhibiting downstream apoptosis signaling. Although cIAP-1/2 were shown to be targets of SM in multiple tumor settings, affecting NF-κB signaling and SM-mediated apoptosis (Bai *et al*, 2009; Gyrd-Hansen & Meier, 2010; Berger *et al*, 2011; Richmond *et al*, 2016; Silke & Vince, 2017) we show here that SM-mediated sensitization of r/r-ALL for cytostatic drugs was independent of NF-κB, RIPK1 and

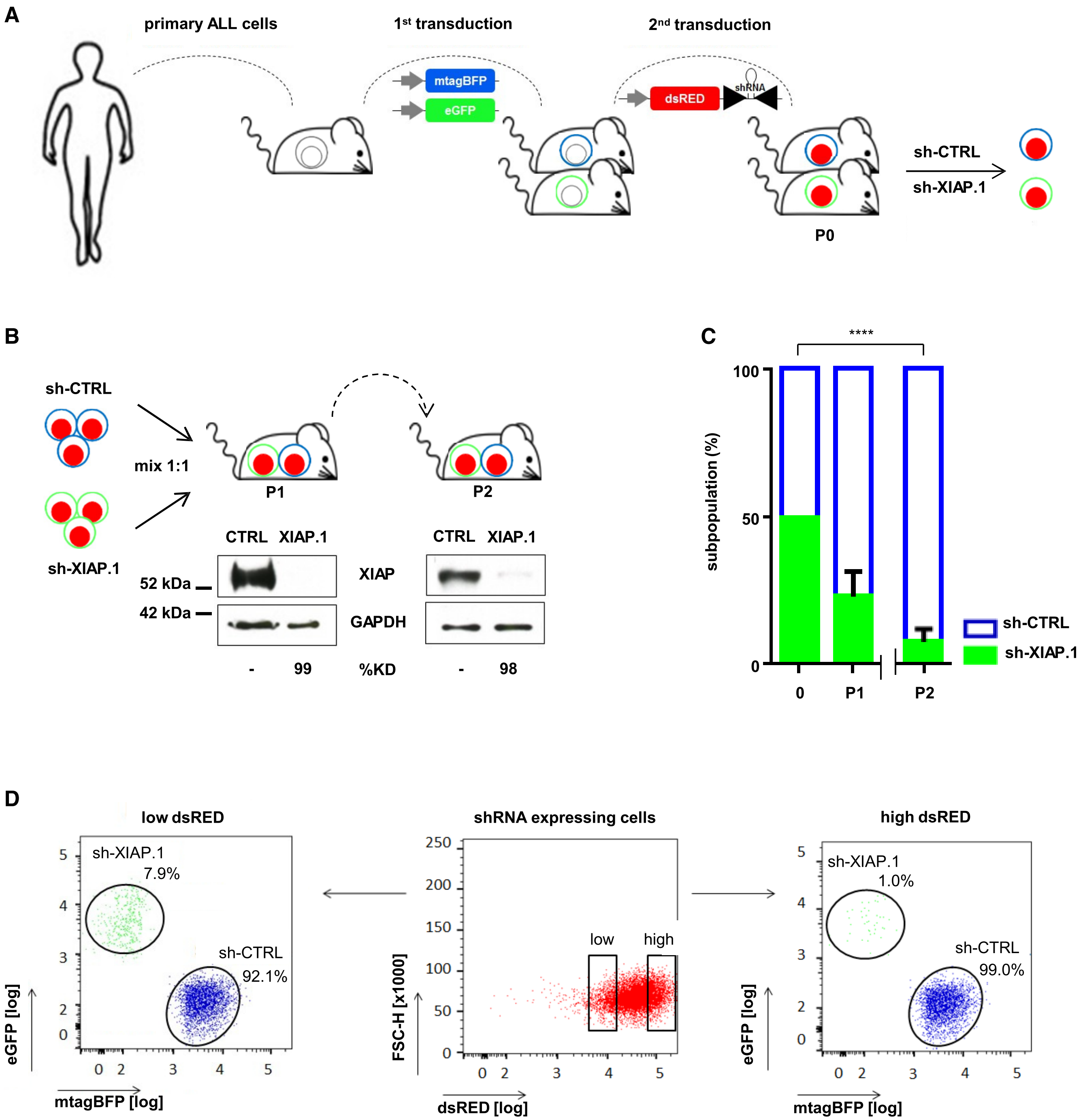

Figure 5.

 © 2022 The Authors

Figure 5. XIAP plays an essential role for patients' r/r ALL cells growing in mice.

A Generation of ALL genetically-engineered PDX (GEPDX) models. Primary patients'ALL-265 cells were transplanted into mice to generate serially transplantable PDX cells. PDX cells were lentivirally transduced in two rounds with the constructs depicted in Fig 4A, and fluorochrome$^+$ PDX cells were enriched by flow cytometric cell sorting; resulting cells expressing either sh-CTRL/mTagBFP or sh-XIAP/eGFP were amplified and applied for repetitive *in vivo* transplantation assays.

B, C XIAP plays an essential role for ALL-265 growing in mice. Experimental scheme and stability of XIAP knockdown over passaging. ALL-265 PDX cells produced as described in A were mixed at a 1:1 ratio and injected into mice (P1, $n = 4$). At clinical signs of illness, cells were isolated and re-injected into next recipient mice (P2, $n = 4$). XIAP expression levels of P1 and P2 PDX cells expressing sh-CTRL/mTagBFP or sh-XIAP/eGFP were analyzed by Western blot at the end of each passage; GAPDH was used as loading control (B). Relative proportion of ALL-265 GEPDX cells expressing sh-CTRL/mTagBFP or sh-XIAP/eGFP re-isolated from the BM at the end of each passage was quantified by flow cytometry (C). Data are depicted as mean ± SEM of all mice analyzed ($n = 4$, each passage) with ****$P < 0.0001$ by unpaired Student's t-test.

D Dose–response relationship between strength of XIAP knockdown and growth disadvantage; ALL-265 PDX cells of a representative mouse were analyzed separately for dsRED/shRNA high- and low-expressing cells.

Source data are available online for this figure.

TNFα. In contrast, KD of XIAP phenocopies the effect of SM treatment in sensitizing r/r ALL cells towards chemotherapy, suggesting that the caspase-inhibitor XIAP might be the direct target of SM in r/r ALL. Albeit XIAP degradation was observed in SM-sensitive but not resistant cells upon SM treatment (Richmond *et al*, 2016; Mamriev *et al*, 2020; Hashimoto *et al*, 2021), we did not observe XIAP degradation to be associated with cell death, suggesting that pharmacological XIAP inhibition by SM is sufficient to block its anti-apoptotic function. Identifying an essential role for XIAP, even in the absence of direct apoptotic stimuli, was unexpected, but in line with recent observations that loss of function mutations of XIAP in patients are associated with spontaneous apoptosis of human T-cells (Paracková *et al*, 2020). As putative explanation, XIAP might constantly inhibit constitutive background apoptosis signaling from inside or outside the tumor cell, preventing, for example, "death by default" (LeBrasseur, 2007; Willis *et al*, 2007).

Determining the patient sub-groups that might benefit most from XIAP-directed therapies either as single agents or in combination treatments remain elusive. Our previous studies indicated that high XIAP protein, but not mRNA, level correlated with unresponsiveness to glucocorticoids and an adverse outcome of T-ALL patients (Hundsdoerfer *et al*, 2010). Post-transcriptional upregulation of XIAP by various mechanisms in conjunction with limited therapy response has been reported for multiple cancer entities (Tamm *et al*, 2000; Gu *et al*, 2009; Nishioka *et al*, 2016; Yang *et al*, 2017). However, increased expression of XIAP alone can likely not serve as predictive biomarker (Seeger *et al*, 2010). As gene expression signatures were suggested to predict response to SM in aggressive BCP-ALL subtypes such as Ph-like ALL (Richmond *et al*, 2016; Zinngrebe *et al*, 2020), it will be interesting to determine whether chemo-resistant ETP-ALL can be sensitized to treatment and whether gene expression signatures can predict response in this aggressive subtype (Zhang *et al*, 2012; Anderson *et al*, 2014). Additionally, targeting XIAP together with BCL-2 was recently suggested as promising treatment for aggressive AML subtypes, where certain mutations, karyotypes and TP53 transcriptional activity correlated with response (Hashimoto *et al*, 2021).

It will be interesting to determine whether the caspase-inhibiting function of XIAP is sufficient to block apoptosis or whether, in analogy to cIAP-1/2, XIAP E3-ligase function is necessary to regulate cell survival (Schile *et al*, 2008; Edison *et al*, 2017; Silke & Vince, 2017; Ellwanger *et al*, 2019).

Treatment targeting XIAP might putatively induce adverse effects. Nevertheless and despite broad expression of XIAP in most organ compartments under physiologic conditions (Duckett *et al*, 1996; Marsh *et al*, 2009), the XIAP knockout mouse shows an overall moderate phenotype with normal blood cell counts (Harlin *et al*, 2001), allowing hope for limited adverse effects of therapies targeting XIAP. ALL cells harbor a higher sensitivity towards loss of XIAP compared to their normal counterparts, lymphocytes and their progenitors (Harlin *et al*, 2001; Filipovich *et al*, 2010). In humans, patients with X-linked lymphoproliferative disease type II (XLP-2) lack functional XIAP but show normal blood cell counts under conditions of health (Rigaud *et al*, 2006) and even lymphoproliferation upon infection (Filipovich *et al*, 2010; Pachlopnik Schmid *et al*, 2011). These data support that therapies which strongly target XIAP might still be tolerated in humans.

Our data favor developing selective SM, additional compounds or approaches which strongly antagonize XIAP for use in patients with r/r ALL. The antisense oligonucleotide AEG35156 specifically targets XIAP and was clinically tested in more than 100 patients, leukemia and lymphoma patients among them (Carter *et al*, 2011; LaCasse, 2013). While a phase I/II trial showed encouraging responses to AEG35156 (Schimmer *et al*, 2009), the approach was not pursued further when a phase II trial failed to show major efficacy (Schimmer *et al*, 2011). Our *in vivo* data show that strong XIAP inhibition is required to unmask XIAPs' essential function, while intermediate inhibition is not sufficient. The strength of XIAP inhibition appears crucial for its anti-tumor effect, but was not documented in the phase II trial, which may eventually explain lack of effectivity.

Newly designed potent and selective IAP inhibitors, currently under development (Baggio *et al*, 2018, 2019; Sheng *et al*, 2019; Shu *et al*, 2019; Mamriev *et al*, 2020), hunt for specific residues in the BIR3 domain of XIAP in order to enhance the affinity of these compounds to XIAP, aiming for a potent and selective inhibition. In this context, proteolysis-targeting chimeras (PROTAC) may be of particular interest given their ability to induce target protein degradation coupled to beneficial therapeutic effects (Lai & Crews, 2017; Kostic & Jones, 2020). Our data support developing such compounds and approaches and test them, for example, in r/r ALL and other resistant tumors.

Materials and Methods

Materials

LBW242 and its orthologue LCL161 were kindly provided by Novartis Pharma (Cambridge, USA), bivalent BV6 by Genentech (San Francisco, USA). The small molecule ARTS mimetic A4 was

© 2022 The Authors

purchased from InterBioScreen. Necrostatin-1 was from StressMarq Bioscience. The antagonistic TNFα antibody adalimumab (Humira®) was purchased from Abbott Laboratories.

Ethical statement

Written informed consent was obtained from all patients and from parents/carers in the cases where patients were minors. The study was performed in accordance with the ethical standards of the responsible committee on human experimentation (written approval by Ethikkommission des Klinikums der Ludwig-Maximilians-Universität München, ethikkommission@med.unimuenchen.de, April 15/2008, number 068-08) and with the Helsinki Declaration of 1975, as revised in 2013.

All animal trials were performed in accordance with the current ethical standards of the official committee on animal experimentation (written approval by Regierung von Oberbayern, poststelle@reg-ob.bayern.de, 05/2007, number 55.2-1-54-2531-95-10 and 01/2016, number ROB-55.2Vet-2532.Vet_02–15-193). The experiments conformed to the principles set out in the WMA Declaration of Helsinki and the Department of Health and Human Services Belmont Report.

Mice were kept in animal rooms of the Laboratory Animal Breeding and Husbandry Unit of Helmholtz Zentrum München under specified pathogen-free (SPF) conditions with a 12/12 h light cycle, fully air-conditioned with a temperature of 20–24°C and 45–65% humidity according to Annex A of the European Convention 2007/526 EC. The maximum stocking density of the cages corresponds to Annex III of the 2010/63 EU. The cages were constantly filled with structural enrichment and the animals had unlimited access to food and water. During the experiment, mice were kept in individually ventilated cages (IVCs). Hygiene monitoring was carried out at least quarterly in accordance with the current FELASA recommendation. For animal studies, sample size was estimated based on preliminary experiments. Blinding was not performed.

Cell lines and patient samples

Cell lines, including LOUCY (human T-ALL, resembling the early T-cell precursor ETP-ALL subtype; Ben-Bassat *et al*, 1990, Zhang *et al*, 2012) and NALM-6 (human pre-B-ALL, resembling DUX4-rearranged B-ALL; Hurwitz *et al*, 1979, Yasuda *et al*, 2016), were purchased from DSMZ (German collection of microorganisms and cells, Braunschweig, Germany) and were maintained in RPMI (Life Technologies GmbH, Darmstadt, Germany), supplemented with 2 mM L-glutamine, penicillin/streptomycin and with 20% (LOUCY) and 10% FCS (NALM-6; Invitrogen). All cell lines used in this study were regularly tested to exclude mycoplasma contamination.

Primary bone marrow cells from ALL patients at diagnosis or at relapse with poor *in vivo* response to second-line chemotherapy were isolated by Ficoll gradient centrifugation (Biochrom) of freshly aspirated bone marrow before the onset of chemotherapy. Primary cells (1×10^6/ml) were cultured for no longer than 24 h in RPMI-1640 supplemented with antibiotics, 2 mM L-glutamine and 20% FCS.

Peripheral blood mononuclear cells (PBMCs) used as normal control to compare protein expression in leukemia (Jimenez-Sanchez *et al*, 2020; Nelson *et al*, 2021) were isolated on Ficoll–Hypaque density gradients (Biochrom AG) from 5 ml of heparinized venous blood drawn from healthy volunteer donors.

For ALL-199, primary ALL blasts of second relapse were obtained from a child treated at the Ludwig Maximilians University Children's Hospital. ALL-265 was obtained from a child treated for relapsed ALL in Zurich according to the I-BFM study protocol and first cell engraftment in mice was performed in Zurich. Engraftment and passaging of PDX cells in NSG mice were performed as described (Terziyska *et al*, 2012).

Detection of apoptosis

Apoptosis was detected by flow cytometry after staining the cells with Annexin-V/propidium iodide (PI; Becton Dickinson). Apoptosis was quantified using CellQuest or FlowJo software with Annexin-positive and Annexin- and PI-double-positive cells regarded as "apoptotic." Apoptosis rate of untreated control cells was subtracted to determine "specific apoptosis."

Determination of mitochondrial membrane depolarization ($\Delta\psi_m$)

To detect the mitochondrial membrane depolarization, the JC-1 Assay Reagent (#10009908, Biozol) was used. Briefly, 1×10^5 cells in 200 µl culture medium were stained with 2 µl of the membrane potential-sensitive JC-1 reagent for 30 min at 37°C. Cells with decreased red fluorescence were detected by FACS. Cells with low $\Delta\psi_m$ were quantified as the percentage of the total cell population.

Detection of caspase activity by flow cytometry

Detection of active-caspase-3 was conducted using FITC-Active-Caspase-3 Apoptosis kit (BD Pharmingen) according to the manufacturer's protocol. Active caspase-8 and active caspase-9 were detected using Green Caspase-8 (or -9) Staining Kit (PromoKine) following instructions of the manufacturer.

Gene silencing by siRNA transfection

For knock-down of RIPK1, NEMO and RelB, cells were transiently transfected with a set of 4 siRNAs (on-target plus smart pool, Dharmacon) using the amaxa nucleofection kit T (amaxa biosystems). Briefly, 2×10^6 cells were transfected with 200 nM target-specific or non-targeting (scrambled) siRNAs and seeded in 12-well plates. After 6 h, cells were treated as indicated to assess apoptosis 48 h post-transfection.

Analysis of protein expression

Cell lysates were prepared using RIPA-buffer supplemented with complete protease inhibitor cocktail (Roche) and phosphatase inhibitor mix II (Serva). Protein concentration was measured by BCA assay (New England Biolabs).

Two different techniques were used to detect protein expression.

Western blot

Equal amounts of protein were separated on gels and transferred by semi-dry blotting (Bio-Rad) to a nitrocellulose membrane (M&N), incubated with antibodies and visualized with chemiluminescent

© 2022 The Authors

substrate (Thermo scientific). The following antibodies were used: XIAP (BD Transduction, 1:5,000), cIAP-1 (R&D, 1:1,000), cIAP-2 (R&D, 1:1,000), actin (Sigma-Aldrich, 1:5,000), GAPDH (CB1001, 1:5,000), caspase-8 (H-134, Santa Cruz, 1:1,000) and RelB (C-19, Santa Cruz, 1:1,000). Antibodies detecting caspase-3, PARP, IKBα, phospho-IKBα (Ser32, 14D4), IKKɣ (NEMO) were from Cell signaling (1:1,000). Secondary antibodies were purchased from GE Health Care. Anti-actin or anti-GAPDH were used to control for equal protein loading.

Simple Western protein immunoassay (WES, ProteinSimple, San Jose, USA)

The technology enables a Western blot-like analysis with the advantage of requiring only low cell numbers, typically 10,000 cells per lane. It was used when only minor cell numbers were available for analysis. In this assay, equal amounts of protein were loaded on an assay plate and samples were processed using a Protein Simple instrument (capillary tube-based electrophoresis immunoassay—San Jose, CA, USA). Capillaries were processed to attach all proteins to the capillary wall and incubated with a single antibody; results were measured as emission curves from each capillary and "Western blot-like presentations" calculated thereof using the Compass software (ProteinSimple), including quantification. Final "Western blot-like presentations" appear clearly different from conventional Western blots (Liu *et al*, 2020); due to very high sensitivity, equal loading is hard to achieve, but also not required, as protein amounts are calculated with high sensitivity and reliability. Primary antibodies used were: XIAP, PARP (both from Cell Signaling Technologies, diluted 1:5 and 1:50, respectively) and ß-actin (AC-15, Sigma Aldrich, 1:25).

Gene expression analysis

Gene expression analysis used the pooled HGU-133 A, B or Affymetrix HGU-133 Plus 2.0 bulk chip data of adult T- and B-ALL (n = 306) and AML (n = 517) patients and healthy controls (n = 10) studied between 1999 and 2005. The routine diagnostic work-up included standard morphology, immunophenotyping, fluorescent in situ hybridization (FISH), cytogenetics and molecular analyses of frequent translocations as published previously (Fransecky *et al*, 2016; Herold *et al*, 2017, 2018). Details of sample preparation, hybridization and image acquisition have been described previously (Herold *et al*, 2018). The data sets are publicly available through the Gene Expression Omnibus (GEO) website (GSE66006, GSE78132, GSE37642).

Results obtained by the HG-U133 A, B chips and HG-U133 Plus 2.0 chips were normalized separately by the robust multichip average method as described by Irizarry *et al* (Hobbs *et al*, 2003), and only the 44,754 probe sets present on all chips were included in the analysis. To correct the batch effect resulting from the use of different chip designs, we applied an empirical Bayesian method as described elsewhere (Herold *et al*, 2014).

For gene expression analysis on NALM-6 cells following moderate or strong XIAP inhibition, NALM-6 cells transduced with different lentiviral vectors (EF1α/sh-CTRL or sh-XIAP.1; SFFV/sh-CTRL or sh-XIAP.1) were sorted 10 and 13 days after transduction. Gene expression analysis was performed by applying a bulk-adjusted SCRB-Seq protocol on sorted subpopulations from NALM-6 cells as described previously (preprint: Soumillon *et al*, 2014; Ebinger *et al*, 2020). Five samples identified as outliers by PCA were excluded from the analysis, as well as genes with only 0 or 1 count per million in more than 2/3 of the samples. Raw count values were preprocessed with the R package *edgeR* (version 3.30.3), and the genes were tested for differential expression with the R package *limma* (version 3.44.3). Genes with an adjusted P-value of ≤ 0.25 were considered significant.

Statistics

Combination indices (CI) were calculated using the Chou-Talay method to determine synergism (CI < 1), additivity (CI = 1) or antagonism (CI > 1), if combining LBW242 with cytotoxic drugs (Chou, 2006). The CI was calculated according to the classic isobologram equation: $CI = (d_1/D_1) + (d_2/D_2)$, where D1 and D2 represent the required doses of drug 1 and drug 2 to produce x% effect and d1 and d2 the required doses of drug 1 and 2 to produce the same effect if used in combination. Statistical significance was determined using Student's t-test (two-tailed) or ANOVA. The gene expression group tests were analyzed by Games–Howell *post hoc* test. A P-value ≤ 0.05 was considered significant if not otherwise stated.

Cloning

XIAP over-expression: For over-expression of XIAP, the full-length CDS of XIAP (gBlock, IDT) was cloned into the pCDH-vector backbone (System Biosciences) using EcoRI and BamHI and linked by a T2A fragment to mTagBFP, used as marker.

shRNA expression system: The construct encoding for dsRED and the miR30 KD cassette was generated by cloning the PCR-amplified dsRED-miR30 fragment (TRMPVIR vector; Addgene #27994) into the pCDH-EF1α-MCS-T2A-copGFP vector (SBI, CD521A-1) using primers carrying MfeI and SalI as 5′ and 3′ restriction sites. The target sequences for eGFP (CCAGCCACAACGTCTA TATCAT) and XIAP (sh-XIAP.1 AAAGCATCATACTATAACTGAA and sh-XIAP.2 CGAGGTTGGTTGTTGTGTTTTA) have been synthetized as part of 110 bp ss-DNA oligos (shRNAmiR30 backbone, Eurofins), annealed and cloned into the vector using XhoI and EcoRI. To generate the plasmids encoding the two different fluorescent markers (mTagBFP or eGFP), the coding sequences for mTagBFP (from plasmid pmTagBFP-C1) and eGFP (from DNA fragment synthetized by IDT) were PCR-amplified and cloned into the same vector as above using BamHI and SalI.

Lentiviral transduction and flow cytometry

ALL cell lines and PDX ALL cells were lentivirally transduced using third-generation packaging plasmids pMDLg/pRRE, pRSV-Rev and pMD2-G as described. Transduction efficiency was optimized to be below 30% in all transduced cell lines and PDX samples to aim for single lentiviral integrations. Freshly transduced PDX ALL cells were kept in culture for 4 days on MS-5 feeder cells to allow transgene expression and enrichment by flow cytometry before injection into mice, to save one round of passaging through mice. Cells were analyzed at the LSRII or sorted using a FACSAriaIII sorter (both Becton Dickinson).

© 2022 The Authors

The paper explained

Problem
Acute lymphoblastic leukemia (ALL) represents the single most frequent cancer in childhood and relapsed/refractory (r/r) disease remains challenging to treat. Novel treatment options are intensively needed to improve prognosis and survival rates. Targeting inhibitor of apoptosis proteins (IAPs) using small molecule SMAC mimetics (SM) is a promising therapeutic approach for r/r ALL as IAPs are often overexpressed, affecting treatment outcome.

Results
When r/r ALL cells revealed complete resistance to SM, SM nevertheless sensitized r/r ALL towards conventional chemotherapy. Mechanistically, SM augmented therapy response independent of TNFα, RIPK1, NFκB and cIAP1/2. Instead, addressing X-linked IAP (XIAP) mimicked the effect of SM and moderate shRNA-mediated knockdown of XIAP increased the anti-tumor effect of chemotherapy. Even beyond, strong XIAP inhibition revealed an essential function of XIAP in patient-derived xenograft models of r/r ALL *in vivo*, even in the absence of chemotherapy.

Impact
These results suggest that targeting XIAP represents an attractive therapeutic approach for ALL upon treatment resistance or relapse.

Competitive *in vivo* transplantation assays

To study the effect of XIAP silencing in NALM-6 and PDX cells, a total amount of 1×10^5 NALM-6 cells, 3×10^5 ALL-199 or 3×10^4 ALL-265 PDX cells expressing either the sh-CTRL or sh-XIAP.1 were mixed 1:1 and injected into the tail vein of 6 to 16-week-old male or female NOD.Cg-Prkdc^{scid} IL2rg^{tm1Wjl}/SzJ mice (NSG, The Jackson Laboratory, Bar Harbour, ME, USA). Experimental end points were specific days after transplantation, or end-stage leukemia when mice developed clinical signs of illness (rough fur, hunchback). Mice were sacrificed by cervical dislocation, and NALM-6 or PDX cells were re-isolated from murine bone marrow and analyzed by flow cytometry or sorted to separate sh-CTRL and sh-XIAP populations according to specific color expression. In treatment trials, mice were randomized and received Vincristine 0.3 mg/kg i.v. once a week for three consecutive weeks or PBS as control. Mice were monitored daily to analyze treatment-related toxicity. Experiments were performed in a non-blinded way. If not indicated differently, three mice were used per group per time point.

Data availability

The gene expression data generated in this study have been deposited at the Gene Expression Omnibus under the following accession code GSE184011 (http://www.ncbi.nlm.nih.gov/geo/query/acc.cgi?acc=GSE184011), GSE66006 (http://www.ncbi.nlm.nih.gov/geo/query/acc.cgi?acc=GSE66006), GSE78132 (http://www.ncbi.nlm.nih.gov/geo/query/acc.cgi?acc=GSE78132), GSE37642 (http://www.ncbi.nlm.nih.gov/geo/query/acc.cgi?acc=GSE37642).

Expanded View for this article is available online.

Acknowledgements
The authors thank Liliana Mura, Fabian Klein, Maike Fritschle, Annette Frank and Miriam Krekel for excellent technical assistance; Markus Brielmeier and team (Research Unit Comparative Medicine) for animal care services, and Binje Vick for critically reading the manuscript. The low-molecular-weight Smac mimetics LBW242 and LCL161 were generously provided by Novartis Pharma (Cambridge, USA). The work was supported by the Humboldt Research Fellowship (to MC); the Physician Scientists Grant (G-509200-004) from the Helmholtz Zentrum München (to TH); by grants of the Berliner Krebsgesellschaft (HUFF200803), the Deutsche José Carreras Leukämie Stiftung (DJCLS R 09/27) and KINDerLEBEN (all to PH) and by grants from the European Research Council Consolidator Grant 681524; a Mildred Scheel Professorship by German Cancer Aid; German Research Foundation (DFG) Collaborative Research Center 1243 project A05; DFG proposal MA 1876/13-1; Bettina Bräu Stiftung and Dr. Helmut Legerlotz Stiftung (all to IJ). Open Access funding enabled and organized by Projekt DEAL.

Author contributions
Michela Carlet: Conceptualization; data curation; formal analysis; validation; investigation; visualization; methodology; writing – original draft; project administration; writing – review and editing. **Karin Schmelz:** Conceptualization; data curation; formal analysis; validation; investigation; visualization; methodology; writing – original draft; project administration; writing – review and editing. **Jenny Vergalli:** Data curation; formal analysis; validation; investigation; visualization; methodology. **Tobias Herold:** Formal analysis; visualization; methodology. **Daniela Senft:** Writing – review and editing. **Vindi Jurinovic:** Formal analysis; visualization. **Thomas Hoffmann:** Methodology. **Jutta Proba:** Methodology. **Nina Weichert:** Methodology. **Christian Junghanß:** Resources. **Mareike Roth:** Methodology. **Georg Eschenburg:** Resources. **Malwine Barz:** Data curation. **Günter Henze:** Resources. **Cornelia Eckert:** Methodology. **Angelika Eggert:** Methodology. **Johannes Zuber:** Methodology. **Patrick Hundsdoerfer:** Conceptualization; resources; data curation; supervision; funding acquisition; validation; investigation; visualization; writing – original draft. **Irmela Jeremias:** Conceptualization; resources; data curation; supervision; funding acquisition; validation; investigation; visualization; writing – original draft; project administration; writing – review and editing.

Disclosure and competing interests statement
The authors declare that they have no conflict of interest.

References

Aguadé-Gorgorió J, McComb S, Eckert C, Guinot A, Marovca B, Mezzatesta C, Jenni S, Abduli L, Schrappe M, Dobay MP *et al* (2020) TNFR2 is required for RIP1-dependent cell death in human leukemia. *Blood Adv* 4: 4823–4833

Anderson NM, Harrold I, Mansour MR, Sanda T, McKeown M, Nagykary N, Bradner JE, Lan Zhang G, Look AT, Feng H (2014) BCL2-specific inhibitor ABT-199 synergizes strongly with cytarabine against the early immature LOUCY cell line but not more-differentiated T-ALL cell lines. *Leukemia* 28: 1145–1148

Baggio C, Gambini L, Udompholkul P, Salem AF, Aronson A, Dona A, Troadec E, Pichiorri F, Pellecchia M (2018) Design of Potent pan-IAP and Lys-covalent XIAP selective inhibitors using a thermodynamics driven approach. *J Med Chem* 61: 6350–6363

Baggio C, Udompholkul P, Gambini L, Salem AF, Jossart J, Perry JJP, Pellecchia M (2019) Aryl-fluorosulfate-based lysine covalent pan-inhibitors of apoptosis protein (IAP) antagonists with cellular efficacy. *J Med Chem* 62: 9188–9200

© 2022 The Authors

Bai L, Chen W, Chen W, Wang X, Tang H, Lin Y (2009) IKKbeta-mediated nuclear factor-kappaB activation attenuates smac mimetic-induced apoptosis in cancer cells. *Mol Cancer Ther* 8: 1636 – 1645

Belz K, Schoeneberger H, Wehner S, Weigert A, Bonig H, Klingebiel T, Fichtner I, Fulda S (2014) Smac mimetic and glucocorticoids synergize to induce apoptosis in childhood ALL by promoting ripoptosome assembly. *Blood* 124: 240 – 250

Ben-Bassat H, Shlomai Z, Kohn G, Prokocimer M (1990) Establishment of a human T-acute lymphoblastic leukemia cell line with a (16;20) chromosome translocation. *Cancer Genet Cytogenet* 49: 241 – 248

Berger R, Jennewein C, Marschall V, Karl S, Cristofanon S, Wagner L, Vellanki SH, Hehlgans S, Rodel F, Debatin KM *et al* (2011) NF-kappaB is required for Smac mimetic-mediated sensitization of glioblastoma cells for gamma-irradiation-induced apoptosis. *Mol Cancer Ther* 10: 1867 – 1875

Brumatti G, Ma C, Lalaoui N, Nguyen NY, Navarro M, Tanzer MC, Richmond J, Ghisi M, Salmon JM, Silke N *et al* (2016) The caspase-8 inhibitor emricasan combines with the SMAC mimetic birinapant to induce necroptosis and treat acute myeloid leukemia. *Sci Transl Med* 8: 339ra69

Byrne AT, Alférez DG, Amant F, Annibali D, Arribas J, Biankin AV, Bruna A, Budinská E, Caldas C, Chang DK *et al* (2017) Interrogating open issues in cancer precision medicine with patient-derived xenografts. *Nat Rev Cancer* 17: 254 – 268

Carlet M, Völse K, Vergalli J, Becker M, Herold T, Arner A, Senft D, Jurinovic V, Liu WH, Gao Y *et al* (2021) In vivo inducible reverse genetics in patients' tumors to identify individual therapeutic targets. *Nat Commun* 12: 5655

Carter BZ, Mak DH, Morris SJ, Borthakur G, Estey E, Byrd AL, Konopleva M, Kantarjian H, Andreeff M (2011) XIAP antisense oligonucleotide (AEG35156) achieves target knockdown and induces apoptosis preferentially in $CD34^{+}38^{-}$ cells in a phase 1/2 study of patients with relapsed/refractory AML. *Apoptosis* 16: 67 – 74

Chou TC (2006) Theoretical basis, experimental design, and computerized simulation of synergism and antagonism in drug combination studies. *Pharmacol Rev* 58: 621 – 681

Condon SM, Mitsuuchi Y, Deng Y, LaPorte MG, Rippin SR, Haimowitz T, Alexander MD, Kumar PT, Hendi MS, Lee YH *et al* (2014) Birinapant, a smac-mimetic with improved tolerability for the treatment of solid tumors and hematological malignancies. *J Med Chem* 57: 3666 – 3677

Duckett CS, Nava VE, Gedrich RW, Clem RJ, Van Dongen JL, Gilfillan MC, Shiels H, Hardwick JM, Thompson CB (1996) A conserved family of cellular genes related to the baculovirus iap gene and encoding apoptosis inhibitors. *EMBO J* 15: 2685 – 2694

Eaves CJ (2015) Hematopoietic stem cells: concepts, definitions, and the new reality. *Blood* 125: 2605 – 2613

Ebinger S, Zeller C, Carlet M, Senft D, Bagnoli JW, Liu WH, Rothenberg-Thurley M, Enard W, Metzeler KH, Herold T *et al* (2020) Plasticity in growth behavior of patients' acute myeloid leukemia stem cells growing in mice. *Haematologica* 105: 2855 – 2860

Edison N, Curtz Y, Paland N, Mamriev D, Chorubczyk N, Haviv-Reingewertz T, Kfir N, Morgenstern D, Kupervaser M, Kagan J *et al* (2017) Degradation of Bcl-2 by XIAP and ARTS promotes apoptosis. *Cell Rep* 21: 442 – 454

Ellwanger K, Briese S, Arnold C, Kienes I, Heim V, Nachbur U, Kufer TA (2019) XIAP controls RIPK2 signaling by preventing its deposition in speck-like structures. *Life Sci Alliance* 2: e201900346

Elsarraj HS, Valdez KE, Hong Y, Grimm SL, Ricci LR, Fan F, Tawfik O, May L, Cusick T, Inciardi M *et al* (2017) NEMO, a transcriptional target of estrogen and progesterone, is linked to tumor suppressor PML in breast cancer. *Cancer Res* 77: 3802 – 3813

Eschenburg G, Eggert A, Schramm A, Lode HN, Hundsdoerfer P (2012) Smac mimetic LBW242 sensitizes XIAP-overexpressing neuroblastoma cells for TNF-alpha-independent apoptosis. *Cancer Res* 72: 2645 – 2656

Evrard YA, Srivastava A, Randjelovic J, Doroshow JH, Dean DA II, Morris JS, Chuang JH, Consortium NCIP (2020) Systematic establishment of robustness and standards in patient-derived xenograft experiments and analysis. *Cancer Res* 80: 2286 – 2297

Fakler M, Loeder S, Vogler M, Schneider K, Jeremias I, Debatin K-M, Fulda S (2009) Small molecule XIAP inhibitors cooperate with TRAIL to induce apoptosis in childhood acute leukemia cells and overcome Bcl-2–mediated resistance. *Blood* 113: 1710 – 1722

Fellmann C, Hoffmann T, Sridhar V, Hopfgartner B, Muhar M, Roth M, Lai DY, Barbosa IA, Kwon JS, Guan Y *et al* (2013) An optimized microRNA backbone for effective single-copy RNAi. *Cell Rep* 5: 1704 – 1713

Filipovich AH, Zhang K, Snow AL, Marsh RA (2010) X-linked lymphoproliferative syndromes: brothers or distant cousins? *Blood* 116: 3398 – 3408

Fransecky L, Neumann M, Heesch S, Schlee C, Ortiz-Tanchez J, Heller S, Mossner M, Schwartz S, Mochmann LH, Isaakidis K *et al* (2016) Silencing of GATA3 defines a novel stem cell-like subgroup of ETP-ALL. *J Hematol Oncol* 9: 95

Fulda S (2017) Smac Mimetics to therapeutically target IAP proteins in cancer. *Int Rev Cell Mol Biol* 330: 157 – 169

Fulda S, Vucic D (2012) Targeting IAP proteins for therapeutic intervention in cancer. *Nat Rev Drug Discov* 11: 109 – 124

Gao H, Korn JM, Ferretti S, Monahan JE, Wang Y, Singh M, Zhang C, Schnell C, Yang G, Zhang Y *et al* (2015) High-throughput screening using patient-derived tumor xenografts to predict clinical trial drug response. *Nat Med* 21: 1318 – 1325

Gerges S, Rohde K, Fulda S (2016) Cotreatment with Smac mimetics and demethylating agents induces both apoptotic and necroptotic cell death pathways in acute lymphoblastic leukemia cells. *Cancer Lett* 375: 127 – 132

Gu L, Zhu N, Zhang H, Durden DL, Feng Y, Zhou M (2009) Regulation of XIAP translation and induction by MDM2 following irradiation. *Cancer Cell* 15: 363 – 375

Guo S, Qian W, Cai J, Zhang L, Wery JP, Li QX (2016) Molecular pathology of patient tumors, patient-derived xenografts, and cancer cell lines. *Cancer Res* 76: 4619 – 4626

Gyrd-Hansen M, Meier P (2010) IAPs: from caspase inhibitors to modulators of NF-kappaB, inflammation and cancer. *Nat Rev Cancer* 10: 561 – 574

Harlin H, Reffey SB, Duckett CS, Lindsten T, Thompson CB (2001) Characterization of XIAP-deficient mice. *Mol Cell Biol* 21: 3604 – 3608

Hashimoto M, Saito Y, Nakagawa R, Ogahara I, Takagi S, Takata S, Amitani H, Endo M, Yuki H, Ramilowski JA *et al* (2021) Combined inhibition of XIAP and BCL2 drives maximal therapeutic efficacy in genetically diverse aggressive acute myeloid leukemia. *Nat Cancer* 2: 340 – 356

Heckl BC, Carlet M, Vick B, Roolf C, Alsadeq A, Grunert M, Liu W-H, Liebl A, Hiddemann W, Marschalek R *et al* (2019) Frequent and reliable engraftment of certain adult primary acute lymphoblastic leukemias in mice. *Leuk Lymphoma* 60: 848 – 851

Herold T, Metzeler KH, Vosberg S, Hartmann L, Röllig C, Stölzel F, Schneider S, Hubmann M, Zellmeier E, Ksienzyk B *et al* (2014) Isolated trisomy 13 defines a homogeneous AML subgroup with high frequency of mutations in spliceosome genes and poor prognosis. *Blood* 124: 1304 – 1311

Herold T, Schneider S, Metzeler KH, Neumann M, Hartmann L, Roberts KG, Konstandin NP, Greif PA, Braundl K, Ksienzyk B *et al* (2017) Adults with Philadelphia chromosome-like acute lymphoblastic leukemia frequently

© 2022 The Authors

have IGH-CRLF2 and JAK2 mutations, persistence of minimal residual disease and poor prognosis. *Haematologica* 102: 130 – 138

Herold T, Jurinovic V, Batcha AMN, Bamopoulos SA, Rothenberg-Thurley M, Ksienzyk B, Hartmann L, Greif PA, Phillippou-Massier J, Krebs S *et al* (2018) A 29-gene and cytogenetic score for the prediction of resistance to induction treatment in acute myeloid leukemia. *Haematologica* 103: 456 – 465

Hobbs B, Collin F, Antonellis KJ, Irizarry RA, Speed TP, Scherf U, Beazer-Barclay YD (2003) Exploration, normalization, and summaries of high density oligonucleotide array probe level data. *Biostatistics* 4: 249 – 264

Hochreiter-Hufford A, Ravichandran KS (2013) Clearing the dead: apoptotic cell sensing, recognition, engulfment, and digestion. *Cold Spring Harb Perspect Biol* 5: a008748

Hundsdoerfer P, Dietrich I, Schmelz K, Eckert C, Henze G (2010) XIAP expression is post-transcriptionally upregulated in childhood ALL and is associated with glucocorticoid response in T-cell ALL. *Pediatr Blood Cancer* 55: 260 – 266

Hurwitz R, Hozier J, LeBien T, Minowada J, Gajl-Peczalska K, Kubonishi I, Kersey J (1979) Characterization of a leukemic cell line of the pre-B phenotype. *Int J Cancer* 23: 174 – 180

Izumchenko E, Paz K, Ciznadija D, Sloma I, Katz A, Vasquez-Dunddel D, Ben-Zvi I, Stebbing J, McGuire W, Harris W *et al* (2017) Patient-derived xenografts effectively capture responses to oncology therapy in a heterogeneous cohort of patients with solid tumors. *Ann Oncol* 28: 2595 – 2605

Jimenez-Sanchez A, Cybulska P, Mager KL, Koplev S, Cast O, Couturier DL, Memon D, Selenica P, Nikolovski I, Mazaheri Y *et al* (2020) Unraveling tumor-immune heterogeneity in advanced ovarian cancer uncovers immunogenic effect of chemotherapy. *Nat Genet* 52: 582 – 593

Konig HG, Fenner BJ, Byrne JC, Schwamborn RF, Bernas T, Jefferies CA, Prehn JH (2012) Fibroblast growth factor homologous factor 1 interacts with NEMO to regulate NF-kappaB signaling in neurons. *J Cell Sci* 125: 6058 – 6070

Kostic M, Jones LH (2020) Critical assessment of targeted protein degradation as a research tool and pharmacological modality. *Trends Pharmacol Sci* 41: 305 – 317

LaCasse EC (2013) Pulling the plug on a cancer cell by eliminating XIAP with AEG35156. *Cancer Lett* 332: 215 – 224

Lai AC, Crews CM (2017) Induced protein degradation: an emerging drug discovery paradigm. *Nat Rev Drug Discov* 16: 101 – 114

Lalaoui N, Hanggi K, Brumatti G, Chau D, Nguyen NY, Vasilikos L, Spilgies LM, Heckmann DA, Ma C, Ghisi M *et al* (2016) Targeting p38 or MK2 enhances the anti-leukemic activity of Smac-Mimetics. *Cancer Cell* 29: 145 – 158

LeBrasseur N (2007) Death by default. *J Cell Biol* 176: 732

Liu W-H, Mrozek-Gorska P, Wirth A-K, Herold T, Schwarzkopf L, Pich D, Völse K, Melo-Narváez MC, Carlet M, Hammerschmidt W *et al* (2020) Inducible transgene expression in PDX models in vivo identifies KLF4 as a therapeutic target for B-ALL. *Biomark Res* 8: 46

Loder S, Fakler M, Schoeneberger H, Cristofanon S, Leibacher J, Vanlangenakker N, Bertrand MJ, Vandenabeele P, Jeremias I, Debatin KM *et al* (2012) RIP1 is required for IAP inhibitor-mediated sensitization of childhood acute leukemia cells to chemotherapy-induced apoptosis. *Leukemia* 26: 1020 – 1029

Maetzig T, Ruschmann J, Sanchez Milde L, Lai CK, von Krosigk N, Humphries RK (2017) Lentiviral fluorescent genetic barcoding for multiplex fate tracking of leukemic cells. *Mol Ther Methods Clin Dev* 6: 54 – 65

Malard F, Mohty M (2020) Acute lymphoblastic leukaemia. *Lancet* 395: 1146 – 1162

Mamriev D, Abbas R, Klingler FM, Kagan J, Kfir N, Donald A, Weidenfeld K, Sheppard DW, Barkan D, Larisch S (2020) A small-molecule ARTS mimetic promotes apoptosis through degradation of both XIAP and Bcl-2. *Cell Death Dis* 11: 483

Marsh RA, Villanueva J, Zhang K, Snow AL, Su HC, Madden L, Mody R, Kitchen B, Marmer D, Jordan MB *et al* (2009) A rapid flow cytometric screening test for X-linked lymphoproliferative disease due to XIAP deficiency. *Cytometry B Clin Cytom* 76: 334 – 344

McComb S, Aguade-Gorgorio J, Harder L, Marovca B, Cario G, Eckert C, Schrappe M, Stanulla M, von Stackelberg A, Bourquin JP *et al* (2016) Activation of concurrent apoptosis and necroptosis by SMAC mimetics for the treatment of refractory and relapsed ALL. *Sci Transl Med* 8: 339ra70

Morrish E, Brumatti G, Silke J (2020) Future therapeutic directions for Smac-Mimetics. *Cell* 9: 406

Nelson MA, McLaughlin KL, Hagen JT, Coalson HS, Schmidt C, Kassai M, Kew KA, McClung JM, Neufer PD, Brophy P *et al* (2021) Intrinsic OXPHOS limitations underlie cellular bioenergetics in leukemia. *Elife* 10: e63104

Nishioka C, Ikezoe T, Yang J, Yokoyama A (2016) BCR/ABL increases EZH2 levels which regulates XIAP expression via miRNA-219 in chronic myeloid leukemia cells. *Leuk Res* 45: 24 – 32

Pachlopnik Schmid J, Canioni D, Moshous D, Touzot F, Mahlaoui N, Hauck F, Kanegane H, Lopez-Granados E, Mejstrikova E, Pellier I *et al* (2011) Clinical similarities and differences of patients with X-linked lymphoproliferative syndrome type 1 (XLP-1/SAP deficiency) versus type 2 (XLP-2/XIAP deficiency). *Blood* 117: 1522 – 1529

Parackova Z, Milota T, Vrabcova P, Smetanova J, Svaton M, Freiberger T, Kanderova V, Sediva A (2020) Novel XIAP mutation causing enhanced spontaneous apoptosis and disturbed NOD2 signalling in a patient with atypical adult-onset Crohn's disease. *Cell Death Dis* 11: 430

Petersen SL, Wang L, Yalcin-Chin A, Li L, Peyton M, Minna J, Harran P, Wang X (2007) Autocrine TNFalpha signaling renders human cancer cells susceptible to Smac-mimetic-induced apoptosis. *Cancer Cell* 12: 445 – 456

Prokocimer M, Peller S, Ben-Bassat H, Goldfinger N, Rotter V (1998) P53 gene mutation in a T-acute lymphoblastic leukemia cell line (loucy) with t (16:20) and 5q- chromosomal aberrations. *Leuk Lymphoma* 29: 607 – 611

Richmond J, Robbins A, Evans K, Beck D, Kurmasheva RT, Billups CA, Carol H, Heatley S, Sutton R, Marshall GM *et al* (2016) Acute sensitivity of Ph-like acute lymphoblastic leukemia to the SMAC-mimetic Birinapant. *Cancer Res* 76: 4579 – 4591

Rigaud S, Fondaneche MC, Lambert N, Pasquier B, Mateo V, Soulas P, Galicier L, Le Deist F, Rieux-Laucat F, Revy P *et al* (2006) XIAP deficiency in humans causes an X-linked lymphoproliferative syndrome. *Nature* 444: 110 – 114

Safferthal C, Rohde K, Fulda S (2017) Therapeutic targeting of necroptosis by Smac mimetic bypasses apoptosis resistance in acute myeloid leukemia cells. *Oncogene* 36: 1487 – 1502

Salih D, Elena B, Qian S, Stephanie H, Eugen T, Cornelia E, Martin E, Rupert H, Geertruy te K, Lisa W *et al* (2020) Therapeutic targeting of mutant p53 in pediatric acute lymphoblastic leukemia. *Haematologica* 105: 170 – 181

Schile AJ, García-Fernández M, Steller H (2008) Regulation of apoptosis by XIAP ubiquitin-ligase activity. *Genes Dev* 22: 2256 – 2266

Schimmer AD, Estey EH, Borthakur G, Carter BZ, Schiller GJ, Tallman MS, Altman JK, Karp JE, Kassis J, Hedley DW *et al* (2009) Phase I/II trial of AEG35156 X-linked inhibitor of apoptosis protein antisense oligonucleotide combined with idarubicin and cytarabine in patients with relapsed or primary refractory acute myeloid leukemia. *J Clin Oncol* 27: 4741 – 4746

Schimmer AD, Herr W, Hanel M, Borthakur G, Frankel A, Horst HA, Martin S, Kassis J, Desjardins P, Seiter K *et al* (2011) Addition of AEG35156 XIAP

 © 2022 The Authors

antisense oligonucleotide in reinduction chemotherapy does not improve remission rates in patients with primary refractory acute myeloid leukemia in a randomized phase II study. *Clin Lymphoma Myeloma Leuk* 11: 433–438

Schirmer M, Trentin L, Queudeville M, Seyfried F, Demir S, Tausch E, Stilgenbauer S, Eckhoff SM, Meyer LH, Debatin KM (2016) Intrinsic and chemo-sensitizing activity of SMAC-mimetics on high-risk childhood acute lymphoblastic leukemia. *Cell Death Dis* 7: e2052

Seeger JM, Brinkmann K, Yazdanpanah B, Haubert D, Pongratz C, Coutelle O, Krönke M, Kashkar H (2010) Elevated XIAP expression alone does not confer chemoresistance. *Br J Cancer* 102: 1717–1723

Servida F, Lecis D, Scavullo C, Drago C, Seneci P, Carlo-Stella C, Manzoni L, Polli E, Lambertenghi Deliliers G, Delia D *et al* (2011) Novel second mitochondria-derived activator of caspases (Smac) mimetic compounds sensitize human leukemic cell lines to conventional chemotherapeutic drug-induced and death receptor-mediated apoptosis. *Invest New Drugs* 29: 1264–1275

Sharma SK, Straub C, Zawel L (2006) Development of Peptidomimetics targeting IAPs. *Int J Pept Res Ther* 12: 21–32

Sheng ZJ, Shi YM, Xu X, Bellynck S, Zhang K, Du ZY, Xu X, Maurel F, Dong C-Z (2019) Development of XIAP antagonists based on de novo 8,5-fused bicyclic lactams. *ChemistryOpen* 8: 34–40

Shu K, Iwamoto N, Honda K, Kondoh Y, Hirano H, Osada H, Ohno H, Fujii N, Oishi S (2019) Development of Mirror-image screening systems for XIAP BIR3 domain inhibitors. *Bioconjug Chem* 30: 1395–1404

Silke J, Vince J (2017) IAPs and cell death. *Curr Top Microbiol Immunol* 403: 95–117

Soumillon M, Cacchiarelli D, Semrau S, van Oudenaarden A, Mikkelsen TS (2014) Characterization of directed differentiation by high-throughput single-cell RNA-Seq. *bioRxiv* https://doi.org/10.1101/003236 [PREPRINT]

Stegmeier F, Hu G, Rickles RJ, Hannon GJ, Elledge SJ (2005) A lentiviral microRNA-based system for single-copy polymerase II-regulated RNA interference in mammalian cells. *Proc Natl Acad Sci USA* 102: 13212–13217

Tamm I, Kornblau SM, Segall H, Krajewski S, Welsh K, Kitada S, Scudiero DA, Tudor G, Qui YH, Monks A *et al* (2000) Expression and prognostic significance of IAP-family genes in human cancers and myeloid leukemias. *Clin Cancer Res* 6: 1796–1803

Tenev T, Bianchi K, Darding M, Broemer M, Langlais C, Wallberg F, Zachariou A, Lopez J, MacFarlane M, Cain K *et al* (2011) The Ripoptosome, a signaling platform that assembles in response to genotoxic stress and loss of IAPs. *Mol Cell* 43: 432–448

Terziyska N, Castro Alves C, Groiss V, Schneider K, Farkasova K, Ogris M, Wagner E, Ehrhardt H, Brentjens RJ, zur Stadt U *et al* (2012) In vivo imaging enables high resolution preclinical trials on patients' leukemia cells growing in mice. *PLoS ONE* 7: e52798

Townsend EC, Murakami MA, Christodoulou A, Christie AL, Koster J, DeSouza TA, Morgan EA, Kallgren SP, Liu H, Wu SC *et al* (2016) The public repository of xenografts enables discovery and randomized phase II-like trials in mice. *Cancer Cell* 30: 183

Varfolomeev E, Blankenship JW, Wayson SM, Fedorova AV, Kayagaki N, Garg P, Zobel K, Dynek JN, Elliott LO, Wallweber HJ *et al* (2007) IAP antagonists induce autoubiquitination of c-IAPs, NF-kappaB activation, and TNFalpha-dependent apoptosis. *Cell* 131: 669–681

Vince JE, Wong WW, Khan N, Feltham R, Chau D, Ahmed AU, Benetatos CA, Chunduru SK, Condon SM, McKinlay M *et al* (2007) IAP antagonists target cIAP1 to induce TNFalpha-dependent apoptosis. *Cell* 131: 682–693

Willis SN, Fletcher JI, Kaufmann T, van Delft MF, Chen L, Czabotar PE, Ierino H, Lee EF, Fairlie WD, Bouillet P *et al* (2007) Apoptosis initiated when BH3 ligands engage multiple Bcl-2 homologs, not Bax or Bak. *Science* 315: 856–859

Yang CC, Chen YT, Chang YF, Liu H, Kuo YP, Shih CT, Liao WC, Chen HW, Tsai WS, Tan BC (2017) ADAR1-mediated 3' UTR editing and expression control of antiapoptosis genes fine-tunes cellular apoptosis response. *Cell Death Dis* 8: e2833

Yasuda T, Tsuzuki S, Kawazu M, Hayakawa F, Kojima S, Ueno T, Imoto N, Kohsaka S, Kunita A, Doi K *et al* (2016) Recurrent DUX4 fusions in B cell acute lymphoblastic leukemia of adolescents and young adults. *Nat Genet* 48: 569–574

Zhang J, Ding L, Holmfeldt L, Wu G, Heatley SL, Payne-Turner D, Easton J, Chen X, Wang J, Rusch M *et al* (2012) The genetic basis of early T-cell precursor acute lymphoblastic leukaemia. *Nature* 481: 157–163

Zinngrebe J, Schlichtig F, Kraus JM, Meyer M, Boldrin E, Kestler HA, Meyer L-H, Fischer-Posovszky P, Debatin K-M (2020) Biomarker profile for prediction of response to SMAC mimetic monotherapy in pediatric precursor B-cell acute lymphoblastic leukemia. *Int J Cancer* 146: 3219–3231

License: This is an open access article under the terms of the Creative Commons Attribution License, which permits use, distribution and reproduction in any medium, provided the original work is properly cited.

Expanded View Figures

Figure EV1. Apoptosis induction by the SM-drug combination is independent from TNFα and RIPK1.

A–D LOUCY (A, B) or NALM-6 cells (C, D) cells were treated for 48 h with SM and/or VCR and/or TNFα in the presence of the TNFα-neutralizing antibody adalimumab (ADM). Apoptosis induction was determined by flow cytometry upon staining with Annexin-V/PI (A, C, D) and by analyzing active caspase-8 (B). Data are expressed as mean ± SD of three independent experiments; ****P* < 0.001 by unpaired Student's *t*-test.

E Samples from two pediatric patients (ALL-3 and ALL-4; see Table EV1 for details) were treated for 24 h with DXR or VCR and/or LBW242 in combination with adalimumab. Apoptosis induction was determined by flow cytometry upon staining with Annexin-V/PI.

F, G Ripoptosome inhibition does not inhibit apoptosis by the SM-drug combination. NALM-6 cells were treated for 48 h with SM and VCR (E) or SM and TNFα (F) in the presence of the RIPK1-inhibitor Necrostatin-1 (Nec). Apoptosis induction was determined by flow cytometry upon staining with Annexin-V/PI. Data are expressed as mean ± SD of three independent experiments with **P* < 0.05, ***P* < 0.01 by unpaired Student's *t*-test.

© 2022 The Authors

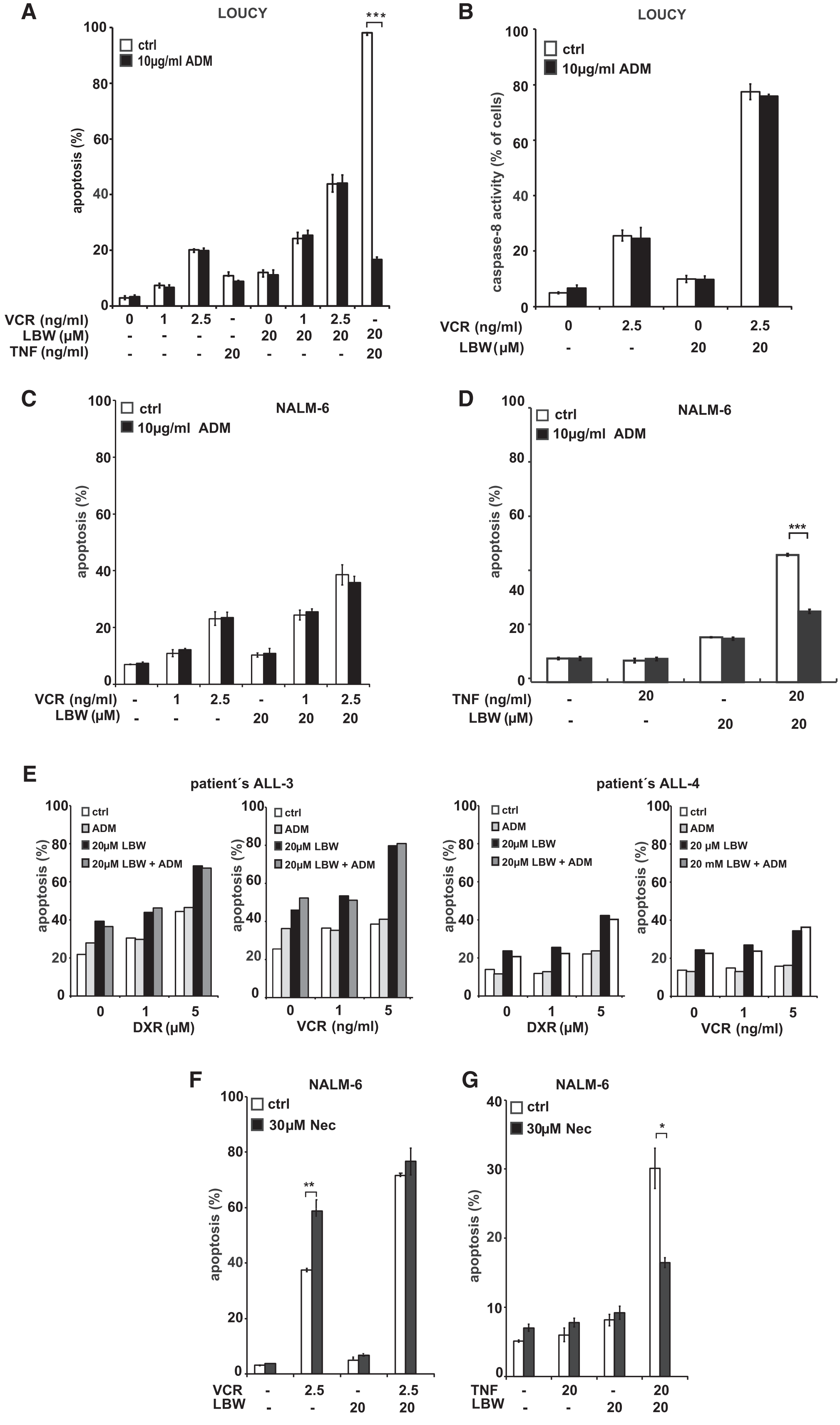
A
LOUCY
ctrl
10µg/ml ADM
apoptosis (%)
VCR (ng/ml)
LBW (µM)
TNF (ng/ml)
***
B
LOUCY
caspase-8 activity (% of cells)
VCR (ng/ml)
LBW (µM)
C
NALM-6
D
NALM-6
TNF (ng/ml)
E
patient´s ALL-3
patient´s ALL-4
ctrl
ADM
20µM LBW
20µM LBW + ADM
20 mM LBW + ADM
DXR (µM)
VCR (ng/ml)
F
NALM-6
30µM Nec
VCR
LBW
**
G
NALM-6
TNF
*

Figure EV1.

Figure EV2. IAP expression in acute leukemias.

A–E mRNA expression of XIAP (A) in different ALL subgroups and mRNA expression of cIAP-1 (B, C) and cIAP-2 (D, E) in samples shown in Fig 3A (B, D) and in distinct ALL subgroups (C, E). Subgroups of ALL significantly differed in their expression levels of all three genes with (A) XIAP: ANOVA $P = 3.74*10^{-6}$; (C) cIAP-1: ANOVA $P = 4.45*10^{-13}$ and (E) cIAP-2: ANOVA $P = 6.89*10^{-14}$. Comparing pairs of two subgroups, statistically significant differences were detected by *post hoc* analysis for a P-value < 0.05 (A) for XIAP in B-other compared to ETV6-t and Ph-positive as well as Burkitt compared to ETV6-t, Hyperdiploidy, Ph-like, Ph-positive and T-ALL, (C) for cIAP-1 in T-ALL compared to B-other, KMT2A-t, Ph-like and Ph-positive (E) for cIAP-2 in Burkitt compared to B-other, KMT2A-t, Ph-positive and TCF3-t as well as for T-ALL compared to B-other, KMT2A-t and Ph-positive and Ph-positive compared to KMT2A-t. Each dot represents one patient sample (biological replicates). Data are expressed as median, 25th and 75th percentile, whiskers indicate min/max; ns (not significant) by Games-Howell *post hoc* test.

 © 2022 The Authors

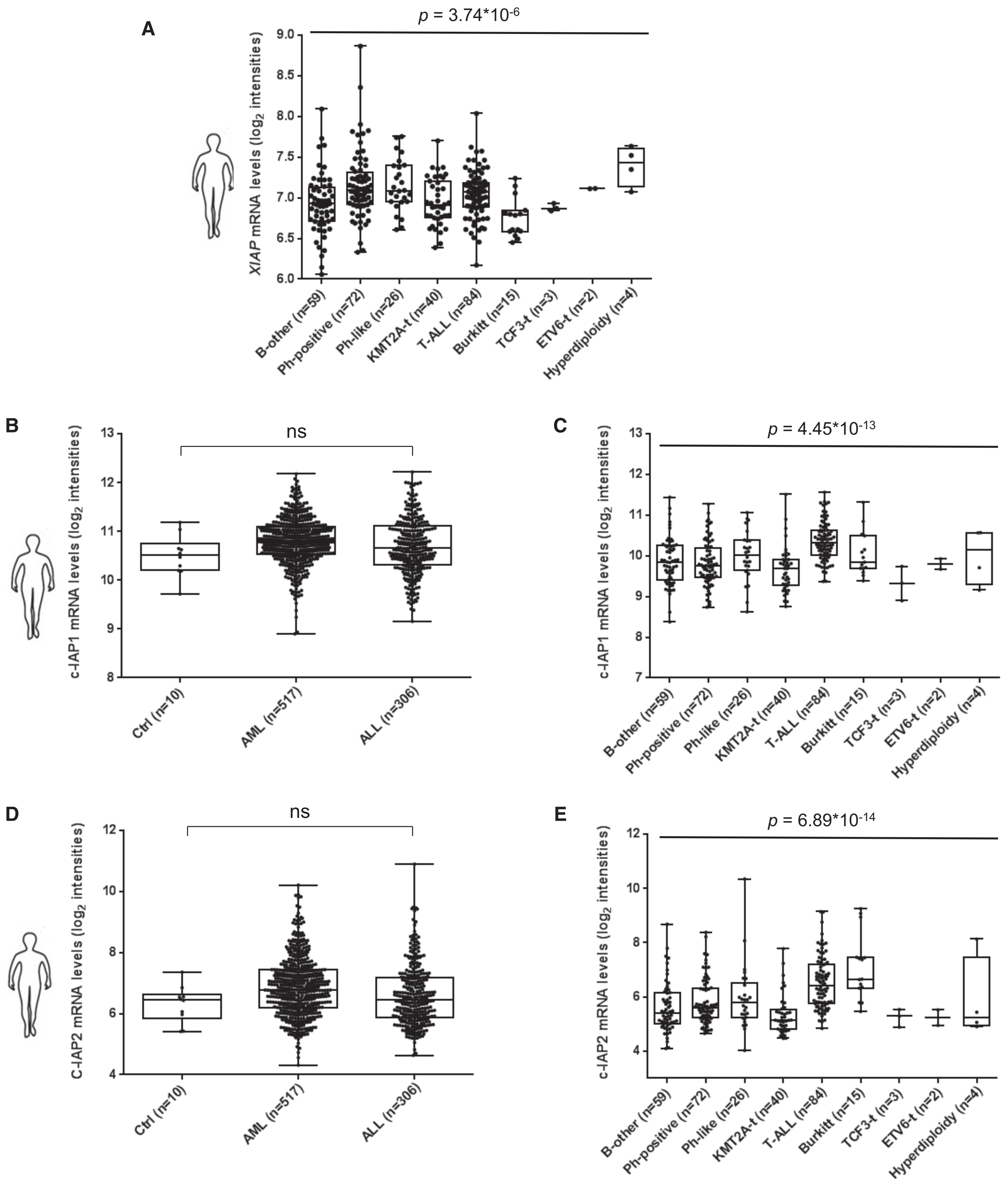
A
p = 3.74*10-6
XIAP mRNA levels (log2 intensities)
9.0
8.5
8.0
7.5
7.0
6.5
6.0
B-other (n=59)
Ph-positive (n=72)
Ph-like (n=26)
KMT2A-t (n=40)
T-ALL (n=84)
Burkitt (n=15)
TCF3-t (n=3)
ETV6-t (n=2)
Hyperdiploidy (n=4)
B
ns
c-IAP1 mRNA levels (log2 intensities)
13
12
11
10
9
8
Ctrl (n=10)
AML (n=517)
ALL (n=306)
C
p = 4.45*10-13
c-IAP1 mRNA levels (log2 intensities)
13
12
11
10
9
8
7
B-other (n=59)
Ph-positive (n=72)
Ph-like (n=26)
KMT2A-t (n=40)
T-ALL (n=84)
Burkitt (n=15)
TCF3-t (n=3)
ETV6-t (n=2)
Hyperdiploidy (n=4)
D
ns
C-IAP2 mRNA levels (log2 intensities)
12
10
8
6
4
Ctrl (n=10)
AML (n=517)
ALL (n=306)
E
p = 6.89*10-14
c-IAP2 mRNA levels (log2 intensities)
12
10
8
6
4
B-other (n=59)
Ph-positive (n=72)
Ph-like (n=26)
KMT2A-t (n=40)
T-ALL (n=84)
Burkitt (n=15)
TCF3-t (n=3)
ETV6-t (n=2)
Hyperdiploidy (n=4)

Figure EV2.

Figure EV3. XIAP plays an essential role for patients' r/r ALL cells growing in mice.

A Control and XIAP-targeting shRNAs are expressed in similar amounts in PDX cells. Experiments were performed as described in Fig 5A, and expression levels of dsRED in the sh-CTRL/mTagBFP and sh-XIAP/eGFP subpopulation were analyzed by FACS.

B Expression of transgenes and control shRNA sequences does not affect proliferation of PDX ALL cells *in vivo*. Competitive *in vivo* assays with different combinations of colors and control shRNA sequences for up to four passages in ALL-265 *in vivo*. Each mouse is considered as one independent experiment (P0, $n = 16$; P1, $n = 16$; P2, $n = 16$; P3, $n = 14$). Data are expressed as mean ± SEM, ns, not significant by unpaired Student's *t*-test.

C, D Experiments were performed and are depicted exactly as described in Fig 5C and D, except that PDX ALL-199 was studied. In (C) each mouse analyzed ($n = 8$) is considered as one independent experiment. Data are expressed as mean ± SEM of all mice analyzed; ****$P < 0.0001$ by unpaired Student's *t*-test. (D) ALL-199 PDX cells of a representative mouse were analyzed separately for dsRED/shRNA high- and low-expressing cells.

Source data are available online for this figure.

 © 2022 The Authors

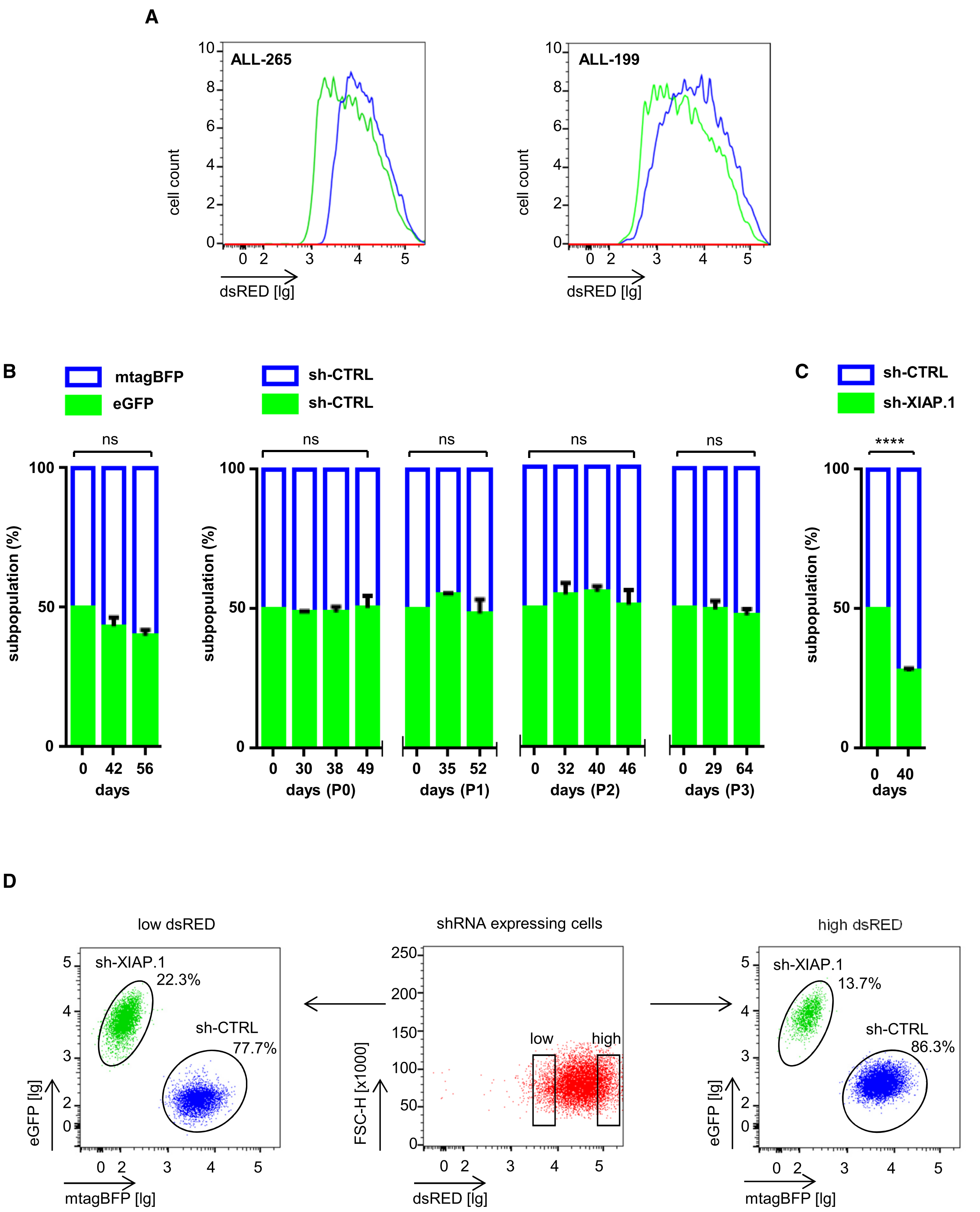
A
ALL-265
ALL-199
cell count
dsRED [lg]
B
mtagBFP
eGFP
sh-CTRL
sh-CTRL
ns
subpopulation (%)
days
days (P0)
days (P1)
days (P2)
days (P3)
C
sh-CTRL
sh-XIAP.1
****
D
low dsRED
shRNA expressing cells
high dsRED
sh-XIAP.1
22.3%
sh-CTRL
77.7%
low
high
13.7%
86.3%
eGFP [lg]
mtagBFP [lg]
FSC-H [x1000]
dsRED [lg]

Figure EV3.
