# Supplementary material for: X‐linked inhibitor of apoptosis protein represents a promising therapeutic target for relapsed/refractory ALL
Source: EMBO Mol Med. 2022 Nov 23;15(1):e14557. doi: 10.15252/emmm.202114557 (PMC9832863; doi:10.15252/emmm.202114557)
Supplement: Supplementary file 12 [file EMMM-15-e14557-s010.pdf]

EMBO Molecular Medicine

XIAP represents a promising therapeutic target for relapsed/refractory ALL

Michela Carlet, Karin Schmelz, Jenny Vergalli, Tobias Herold, Daniela Senft, Vindi Jurinovic, Thomas Hoffman, Jutta Proba, Nina Weichert, Christian Junghanß, Mareike Roth, Georg Eschenburg, Malwine Barz, Günter Henze, Cornelia Eckert, Angelika Eggert, Johannes Zuber, Patrick Hundsdoerfer, and Irmela Jeremias
DOI: 10.15252/emmm.202114557

Corresponding author: Irmela Jeremias (irmela.jeremias@helmholtz-muenchen.de)

Review Timeline:

Submission Date:	30th May 20
Editorial Decision:	25th Jun 20
Revision Received:	14th May 21
Editorial Decision:	17th Jun 21
Revision Received:	29th Nov 21
Editorial Decision:	20th Jan 22
Revision Received:	6th Aug 22
Editorial Decision:	9th Sep 22
Revision Received:	23rd Oct 22
Accepted:	28th Oct 22

Editor: Lise Roth

Transaction Report:

(Note: With the exception of the correction of typographical or spelling errors that could be a source of ambiguity, letters and reports are not edited. Depending on transfer agreements, referee reports obtained elsewhere may or may not be included in this compilation. Referee reports are anonymous unless the Referee chooses to sign their reports.)

25th Jun 2020

Dear Dr. Jeremias,

Thank you for the submission of your manuscript to EMBO Molecular Medicine. We have now received feedback from two of the three reviewers who agreed to evaluate your manuscript. Given that both referees provide very similar recommendations, we prefer to make a decision now in order to avoid further delay in the process.

As you will see, the referees acknowledge the novelty and interest of your findings on the role of XIAP in ALL. However, they also have serious concerns that preclude further consideration of the article at this time (including the lack of mechanistic insight, the over-interpretation of the results in some places, and the limited clinical relevance of the findings at this stage). They realize that addressing these issues would require a lot of additional work, time and effort. As EMBO Press encourages a single round of revision in a reasonable timeframe, we therefore cannot offer to consider your manuscript further at this stage. However, given the potential interest and novelty of the findings, we would be willing to consider a new manuscript on the same topic if at some time in the near future you obtained data that would considerably strengthen the message of the study and address the referees' concerns in full. To be completely clear, however, I would like to stress that if you were to send a new manuscript this would be treated as a new submission rather than a revision and would be reviewed afresh, in particular with respect to the literature and the novelty of your findings at the time of resubmission. If you decide to follow this route, please make sure you nevertheless upload a letter of response to the referees' comments.

I am sorry that I could not bring better news this time and hope that the referee comments are helpful in your continued work in this area.

Yours sincerely,

Lise Roth

Lise Roth, Ph.D
Editor
EMBO Molecular Medicine

***** Reviewer's comments *****

Referee #1 (Remarks for Author):

In the present manuscript, the authors studied IAPs and Smac mimetics (SM). XIAP was identified to mediate SM effects. Molecular manipulation of XIAP in vivo, in cell lines and PDX of r/r ALL, using microRNA-30 flanked sh-RNA expression in vivo mimicked SM effects; intermediate XIAP knockdown sensitized r/r ALL cells towards chemotherapy and induced apoptosis. Interestingly, upon strong XIAP knockdown, PDX r/r ALL cells were

outcompeted in vivo, even in the absence of chemotherapy, indicating a yet unknown essential function of XIAP in r/r ALL. Thus the authors suggest that XIAP represents a promising therapeutic target for r/r ALL and XIAP inhibitors might eliminate r/r ALL cells, even as single drugs.

This is an interesting study which identifies the potential 'strategic role' of XIAP in ALL and suggest XIAP as a new target for drug development.

Major points

1-The authors suggest an essential function for XIAP in patients ALL cells growing in vivo (Figure 6E). While partial inhibition of XIAP by moderate KD mimicked the effect of SM and sensitized ALL cells towards chemotherapy (Figures 1-5), strong inhibition of XIAP reduced survival and proliferation of r/r ALL in vivo (Figures 5G and 6). This point is central. How XIAP regulates these events remains unclear. Which pathways of regulation of cell death or of inflammation and immune recognition might be activated upon higher XIAP KD? Essentially, why these cells are less to no tumorigenic, which signaling is responsible? In a complete different setting, it has been reported that X-linked Inhibitor of Apoptosis (XIAP) deficiency predisposes people to pathogen-associated hyperinflammation. Upon XIAP loss, Toll-like receptor (TLR) ligation triggers RIPK3-caspase-8-mediated IL-1b activation and death in myeloid cells. Basically, upon XIAP deficiency, a TLR-TNF-TNFR2 axis drives cIAP1-TRAF2 degradation to allow TLR or TNFR1 activation of RIPK3-caspase-8 and IL-1b (Cell Reports 20, 668-682, 2017). Is it conceivable that these mechanisms may also occur in the XIAP fully deficient ALL settings? Experiments aiming to mechanistically explain the role of high XIAP deficiency should be included.

2-Along those lines, the authors do not really deepen the role of XIAP as E3 ligase. As an example, recently, the ubiquitin E3 ligase XIAP was reported counteracting RIPK2-complex formation (https://doi.org/10.26508/lsa.201900346). Is it possible that XIAP mutants (RING domain?) might be useful to decipher the mechanism by which XIAP downregulation has main effects against ALL?

3-The effects seen are related to de downregualtion/silencing of XIAP. Possibly the pharmacological targeting of XIAP might not only be pursued with small molecules, but also following a PROTAC approach which might turn out also useful in ALL overexpressing the target. The authors may want to critically express their opinion in the discussion on this topic.

4- The in vivo data are mainly obtained in xenograft or PDX models. Would the presence of a fully functional immune system influence the results outcome?

Minor points

-In Fig. 1 and suppl. Fig. 1, the authors show the mixed sensitivity or resistance to a broad rage of IAP inhibitors. The figures might be implemented by showing the expression levels of the hitten IAP targets in the different cell lines used to verify whether (or not) there is any correlation with the reported sensitivity or resistance.

-In Fig. 2 the authors indicate that SM in combination with chemotherapy induced cell death in r/r ALL independently of RIP1, NF-kB or TNFα. This conclusion with particular reference for RIP1 was taken by using NEC1, an inhibitor of RIP1. Despite correct, to validate the real potential contribution of RIP1 it is suggested to use RIP1 KD strategies together with the pharmacologcal inhibition shown. Moreover, is it RIPK3 potentially involved?

-In addition, the authors report that the SM-drug combination did not induce degradation of IkBa (Figure 2B) and apoptosis was not affected by knockdown (KD) of NEMO (Figure 2C), in contrast to published data on SM-signaling alone (Bai, Chen et al., 2009). Which

hypothesis can the authors suggest for this difference? This might be commented in the discussion.
-Blot quantification (Image J, others) when target silencing is shown would be encouraged.

Referee #2 (Comments on Novelty/Model System for Author):

I strongly feel that this manuscript, at this time, does not provide sufficient information to merit publication in EMBO Molecular Medicine. Very drastic modifications and additional experiments would be necessary before it can be considered for publication.

Referee #2 (Remarks for Author):

The manuscript by Carlet et al. shows that treatment with SMAC mimetics induces apoptosis in r/rALL cell lines in a synergistic fashion when used in combination with some chemotherapeutic agents. This action appears to be dependent on inhibition of XIAP. The manuscript contains interesting preliminary data. Indeed, while the data are provocative and point to potentially interesting clinical issues, my enthusiasm is weakened since, on a closer look, the analysis performed by the authors is, in most cases, an over-interpretation. Critical results have been obtained exclusively in cell lines and the clinical issues are, at this time, too theoretical for a journal as EMBO Molecular Medicine. In spite of that, it is important to recognize the enormous amount of work included in this manuscript and the use of interesting and well-designed GEPDX models.

Main concerns:

-In the first part of the manuscript (Figures 1 and 2), the rationale on XIAP implication in ALL is not well explained and it is difficult to follow (Figures/supplementary data). Tests were mainly performed in cell lines and more experiments on primary cells are critically missing.

-The authors seem to over-interpret the results included in the aforementioned figures. A clear example (among others) is the assessment of the intracellular apoptosis signaling activated by the SM-drug combination. Some data are analyzed in the Loucy cell line (caspase activation, independency from TNFa) other in the NALM-6 cell line (NFKb pathway analysis). The results are described in the Results section as a mix of the two cell lines and the conclusion is expressed as a general issue. Additionally, most of the conclusions are negative results without the corresponding positive controls.

-In these figures, only a panel including primary cells is included. This experiment is performed in a single patient (obviously without statistics), making the conclusion also an over-interpretation.

- The effect of the combination of LBW242 and other cytotoxic drugs is defined as synergistic (line 72). However, according to the explanation on the combination indexes included in the legend, it seems that only the combination LBW242 + Vincristine is in fact synergistic. The combination LBW242 + L-Asp appeared to be the addition of the two drugs, while LBW242 + Doxo is antagonistic. How do the authors explain this effect?

- Data included in Supplementary Tables 1 and 2 are exactly the same. This raises doubts about the results.

- In the line 59, the authors claim they continued to use the cell lines NALM-6 and Loucy because they were among the most resistant to LBW treatment. However, in Supplementary Fig 1A there are other cell lines that show similar levels of apoptosis, and also two cell lines that show more resistance to apoptosis (Molt-3 and Molt-4). Why were these cell lines not used in further experiments?

- In correlation with the values presented in Supplementary Tables 1 and 2 for the combination of LBW242 + Doxo, results depicted in Supplementary Figures G and K do not show increased sensitivity of these cell lines to the combined treatment, which does not correspond to the claim made in lines 69-70. Do the authors have an explanation for the difference in the response to LBW242 + Doxo and LBW242 + the other drugs tested?

- In line 86, the authors claim that both c-IAP1 and 2 were degraded by SM treatment. However, the blots ins Supplementary Figs 1S, T, and U show a different behavior between c-IAP 1 and 2. While the sentence seems appropriate for c-IAP1, c-IAP2 goes from being completely absent to being present and does not change depending on the cell type or SM used. How do the authors explain this issue?

- LCL161 is used in Figures 1A and Supplementary Figure 1E, but it is only defined as a LBW242 analogue further in the text (line 68). It should be explained earlier.

- In Supplementary Fig 1O, is there an error in the Vincristine concentration shown above the blot? In Supplementary Fig 1N the concentrations used were 1 and 2.5 ng/ml, while in the blot it shows 1 and 0.25 ng/ml, which seems awkward also because it seems 0.25 ng/ml leads to higher levels of cleaved Caspase-8 than 1 ng/ml. A similar issue is observed in Supplementary Figure 1R and Figure 1F.

- In Figure 2A, cell death seems reduced in presence of Nec-1 + LBW, which does not agree with what is stated in the text. Moreover, cell death seems to increase after Vincristine + Nec-1 treatment, which is also not expected. Are these comparisons statistically relevant? Any explanation?

- In this panel, is the legend correct for the two bars on the right? Or the legend described the LBW (20µM) + TNF 20 ng/mL combination?

- In Fig 2B, what are the time points? This information is not indicated either in the legend or in the Figure.

- In Fig 2C, how do the authors explain the increase in apoptotic cells by both the control and the NEMO siRNA?

- In line 107, the authors write that « NALM-6, SM alone or in combination with chemotherapy induced p100 processing that was "not sufficient to induce apoptosis". Even if it appeared that p100 processing happens specifically after SM treatment (Fig 2D), the results shown in Fig 2E do show that the same combination of VCR + LBW leads to more than 80% of apoptotic cells.

- This reviewer disagrees with the conclusion in lines 114-117. The only issue that can be drawn based on these experiments is that apoptosis caused by SM treatment appears to be independent on RIP1, NF-kB or TNFα.

- A statistical analysis should be included in the entire figure.

- In Supplementary Fig 2A, the combination of L-Asp + Prednisone leads to virtually 100% apoptotic cells, which is greatly decreased to less than 10% by adding Adalimumab. Do the authors have an explanation for this observation, which is clearly different from the combination VCR + Prednisone?

- In Figure 3A, as well as in Supplementary Figures 3B and D, the authors did not use the correct statistical test. There is a huge difference between the sizes of the samples in the control and AML/ALL groups. The Tukey's test assumes equal variance between the groups, and is dependent on both equal variance and similar group sizes for correct comparisons. The information described in the figure legends, as well as in the Methods section, does not describe any such procedure of correction. The reviewer strongly suggests a revision of these statistical tests.

- In Figure 3B, are the comparisons of protein expression values for PBMC and PDX samples statistically significant? Additionally, the results of Supplementary Fig 3G do not look like the quantifications shown in Figure 3B. In fact, most of PDX samples (except ALL-50 and ALL-265) do not seem to have higher levels of XIAP (contrary to what is stated in the Results section).

- In Supplementary Figure 3F, cIAP-1 shows a similar behavior to XIAP (as well as in other figures). The authors do not acknowledge or discuss the fact that some of the effects observed might also be linked to cIAP-1. It it known that SMAC mimetics are more specific towards cIAP-1 and -2, while ARTS mimetics are more specific to XIAP. It would be important to perform at least the most important experiments of this manuscript with ARTS mimetics as well.

- Line 406: "vain" instead "vein". "SCID" should be in capital letters.

- The authors should include a statistical assessment in Supplementary Figures 3A to E.

- As indicated above, the results obtained in the GEPDX models are interesting, original and they have also been well explained and designed. They deserve to be studied more deeply. Indeed, some key questions should be answered before concluding that effects observed were due to different XIAP expression or that there exists a yet unknown survival function for XIAP. For example, it is important: (i) to perform a molecular analysis that could allow the EMBO Molecular Medicine readers to understand the role of XIAP in the absence of direct apoptotic stimuli; (ii) to uncover a molecular tool supporting the idea that there exists a survival role of XIAP; and, (iii) to demonstrate, with molecular/biochemical data, the assertion "strong XIAP inhibition is required to unmask XIAPs essential function, while intermediate inhibition is not sufficient".

26 Jun 2022

Dear Dr. Jeremias,

Please find enclosed the comments from referee #3 for your information.

With my best wishes,

Lise

To the Authors

This is a well designed and generally clearly presented study with new information of interest.

I have the following comments that should be addressed:

1. The study presents data on T-and B-ALL cell lines and patients' samples as if this makes no difference which clearly goes against most thinking about the mechanisms perturbed in these different types of human ALL. At the very least, it would be important to acknowledge this at the outset and state clearly which diseases were being modelled in each set of experiments that form the main body of the paper, especially given the fact that only one or 2 cell lines or patient samples of each type were studied in any given set of experiments.
2. On a similar note, more detail should be provided in the main manuscript on all primary samples (age, sex, diagnosis, ALL type, blast count, main genetic abnormalities, normal bone marrow source, etc.) ideally in a table.
3. No information is provided on how many times experiments were independently repeated. Are error shown just for replicate cultures or mices within a ? representative or single experiment, or between independent experiments.
4. Please provide data on lentiviral gene transfer efficiencies.
5. Data with a single shRNA is not typically considered sufficient to rule out off-target effects contributing to altered biological responses.

Point by Point response to reviewer comments

Carlet et al., EMM-2020-12844

X-linked inhibitor of apoptosis protein represents a promising therapeutic target for relapsed/refractory ALL

We are happy to see that our manuscript (EMM-2020-12844) was well received and thank all reviewers for their careful analysis. Motivated by the constructive, sound and scientifically stimulating comments, we extensively revised the manuscript and performed multiple new experiments, addressing all 40 points raised. New data include

- new molecular data on knockdown of RIPK1 (new Figure 2B)
- new patient data on inhibitor function in primary patient samples (new Figures 1B, and supplemental Figure S2C)
- new functional data on the ARTS mimetic A4 (new supplemental Figure 3H),
- new descriptive data on transcriptome upon XIAP knockdown (new supplemental Figure 4H), protein levels of IAPs (new supplemental Figures 1B and 1N) and signs of apoptosis (new Figure 4H), among others

Detailed responses to each individual point raised by the reviewer can be found below.

Reviewer Comments

Referee #1 (Remarks for Author):

In the present manuscript, the authors studied IAPs and Smac mimetics (SM). XIAP was identified to mediate SM effects. Molecular manipulation of XIAP in vivo, in cell lines and PDX of r/r ALL, using microRNA-30 flanked sh-RNA expression in vivo mimicked SM effects; intermediate XIAP knockdown sensitized r/r ALL cells towards chemotherapy and induced apoptosis. Interestingly, upon strong XIAP knockdown, PDX r/r ALL cells were outcompeted in vivo, even in the absence of chemotherapy, indicating a yet unknown essential function of XIAP in r/r ALL. Thus the authors suggest that XIAP represents a promising therapeutic target for r/r ALL and XIAP inhibitors might eliminate r/r ALL cells, even as single drugs.

This is an interesting study which identifies the potential 'strategic role' of XIAP in ALL and suggest XIAP as a new target for drug development.

We thank the reviewer for the positive judgement, careful analysis and helpful comments which we have addressed in detail below.

Major points

1. The authors suggest an essential function for XIAP in patients ALL cells growing in vivo (Figure 6E). While partial inhibition of XIAP by moderate KD mimicked the effect of SM and sensitized ALL cells towards chemotherapy (Figures 1-5), strong inhibition of XIAP reduced survival and proliferation of r/r ALL in vivo (Figures 5G and 6). This point is central.
   How XIAP regulates these events remains unclear. Which pathways of regulation of cell death or of inflammation and immune recognition might be activated upon higher XIAP KD? Essentially, why these cells are less to no tumorigenic, which signaling is responsible?
   In a complete different setting, it has been reported that X-linked Inhibitor of Apoptosis (XIAP) deficiency predisposes people to pathogen-associated hyperinflammation. Upon XIAP loss, Toll-like receptor (TLR) ligation triggers RIPK3-caspase-8-mediated IL-1b activation and death in myeloid cells. Basically, upon XIAP deficiency, a TLR-TNF-TNFR2 axis drives cIAP1-TRAF2 degradation to allow TLR or TNFR1 activation of RIPK3-caspase-8 and IL-1b (Cell Reports 20, 668-682, 2017). Is it conceivable that these mechanisms may also occur in the XIAP fully deficient ALL settings? Experiments aiming to mechanistically explain the role of high XIAP deficiency should be included.

Authors' Response:

We performed 2 types of new experiments.

a. We performed new transcriptome analysis which revealed that cells with weak XIAP knockdown clustered together with control cells, while cells with strong XIAP knockdown clustered apart and showed signs of activated apoptosis pathways (new supplemental Figure S4H).
b. We now show that cells dying upon strong knockdown of XIAP cleave PARP, indicating apoptosis (new Figure 4H).

An obvious mechanism of apoptosis by high XIAP knockdown might lie in the direct activation of caspases due to lack of the inhibitor XIAP; according to the "death by default" concept (Willis et al. Science 2007, PMID: 17289999), constitutive pro-

death signaling might induce apoptosis in the absence of XIAP, as now discussed in the manuscript on page 19-20.

Immune recognition is rather unlikely as mechanism as PDX models require severely immune compromised mice.

As attractive alternative mechanism, we considered upstream TNFα or IL1β signaling as mentioned by the reviewer. The manuscript cited by the reviewer studies XIAP knockout in the presence of concomitant deregulation of c-IAP1 and c-IAP2, while we study XIAP knockdown without regulation of c-IAP1 and c-IAP2 (Figure 4C), indicating major differences. Our new transcriptome data did not show signs of involvement of the TNFα or IL1β signaling pathway. While leaving posttranslational regulation behind, our studies do not reveal indications of an involvement of TNFα or IL1β signaling.

2. Along those lines, the authors do not really deepen the role of XIAP as E3 ligase. As an example, recently, the ubiquitin E3 ligase XIAP was reported counteracting RIPK2-complex formation (doi.org/10.26508/lsa.201900346). Is it possible that XIAP mutants (RING domain?) might be useful to decipher the mechanism by which XIAP downregulation has main effects against ALL?

The reviewer is right. We agree that XIAP mutants will be of major help for deciphering the mechanism how XIAP downregulation leads to death of ALL cells; we now discuss a putative function of XIAP's E3 ligase in the manuscript (page 20).

3. The effects seen are related to de downregulation/silencing of XIAP. Possibly the pharmacological targeting of XIAP might not only be pursued with small molecules, but also following a PROTAC approach which might turn out also useful in ALL overexpressing the target. The authors may want to critically express their opinion in the discussion on this topic.

We thank the reviewer for this interesting input; we now discuss PROTAC on page 21 as

> *"Newly designed potent and selective IAPs inhibitors, currently under development (40, 66-69), hunt for specific residues in the BIR3 domain of XIAP in order to enhance the affinity of these compounds to XIAP, aiming for a potent and selective inhibition. In this context, proteolysis-targeting chimeras (PROTAC) may be of particular interest given their ability to induce target protein degradation coupled to beneficial therapeutic effects (70, 71)."*

4. The in vivo data are mainly obtained in xenograft or PDX models. Would the presence of a fully functional immune system influence the results outcome?

This question is highly interesting. We would hypothesize that a fully functional immune system might eventually increase cell death in ALL cells dying due to XIAP knockdown as apoptotic cells express "eat me" signals. Alternatively, it might have no effect, while we consider a protective function rather unlikely, but still possible.

Which is now discussed on page 19 as follows:

"While the high clinical relevance of the PDX model is widely accepted (44-48), the absence of a functional immune system remains a potential limitation (49). Therefore, a possible influence of the immune system on the effects of XIAP knockdown in our ALL PDX remains to be determined; as apoptotic cells express "eat me" signals capable to trigger an immune response (50), effects of XIAP knockdown may even be increased."

Minor points

- In Fig. 1 and suppl. Fig. 1, the authors show the mixed sensitivity or resistance to a broad range of IAP inhibitors. The figures might be implemented by showing the expression levels of the hitten IAP targets in the different cell lines used to verify whether (or not) there is any correlation with the reported sensitivity or resistance.

We performed new experiments and now show Western blots in new suppl. Figure S1B. Our new data reveal that expression levels of IAPs do not correlate to sensitivity towards IAP inhibitors.

- In Fig. 2 the authors indicate that SM in combination with chemotherapy induced cell death in r/r ALL independently of RIP1, NF-kB or TNFα. This conclusion with particular reference for RIP1 was taken by using NEC1, an inhibitor of RIP1. Despite correct, to validate the real potential contribution of RIP1 it is suggested to use RIP1 KD strategies together with the pharmacologcal inhibition shown. Moreover, is it RIPK3 potentially involved?

We performed new molecular experiments and generated Loucy cells with knockdown of RIP1. Knockdown of RIPK1 did not influence cell death induction by the combination of LBW and Vincristine, strengthening the notion that this cell death occurs independently from RIPK1 (new Figure 2B).

- In addition, the authors report that the SM-drug combination did not induce degradation of IkBa (Figure 2B) and apoptosis was not affected by knockdown (KD) of NEMO (Figure 2C), in contrast to published data on SM-signaling alone (Bai, Chen et al., 2009). Which hypothesis can the authors suggest for this difference? This might be commented in the discussion.

Bai et al. (PMID:19509265) published that activation of the classical NF-kappaB pathway downstream of TNFα signaling reduces cell death upon treatment with SM in SM-sensitive cells, while we do not observe any influence of the classical NF-kappaB signaling pathway in our SM-resistant cells; neither by SM treatment alone nor in combination with chemotherapy. In accordance, we also demonstrate that apoptosis by the SM-drug combination is independent of TNFα and RIPK1, suggesting that survival and apoptosis are not influenced by the TNFα– NF-kappaB axis. We hypothesize that in SM resistant cells, survival and apoptosis is instead regulated by XIAP, since XIAP knockdown phenocopies effects of SM treatment. This hypothesis is discussed on page 19 as followed:

"Although c-IAP1/2 were shown to be targets of SM in other tumor settings, resulting in NF-κB activation and affecting SM-mediated apoptosis (6, 36-38), we show here that SM-mediated sensitization of r/r-ALL for cytostatic drugs was independent of NF-κB, RIPK1 and TNFα. In contrast, knockdown of XIAP phenocopies the effect of SM treatment in sensitizing r/r ALL cells towards

chemotherapy, suggesting that the caspase-inhibitor XIAP might be the direct target of SM in r/r ALL."

- Blot quantification (Image J, others) when target silencing is shown would be encouraged.

We have added protein quantification underneath the respective Western blots in revised Figures 2B,D,F, Figure 4D and 5B.

Referee #2:

Referee #2 (Comments on Novelty/Model System for Author):

I strongly feel that this manuscript, at this time, does not provide sufficient information to merit publication in EMBO Molecular Medicine. Very drastic modifications and additional experiments would be necessary before it can be considered for publication.

We used innovative GEPDX technology to provide molecular evidences that XIAP plays an essential role in patient-derived tumor cells in vivo. Our manuscript presents molecular based, translationally relevant new data on the role of XIAP during ALL treatment and provides valuable new information which will interest the readers of EMBO Molecular Medicine.

We performed drastic modifications to improve our manuscript. We performed numerous new experiments; new data include

- new molecular data on knockdown of RIPK1 (new Figure 2B)
- new patient data on inhibitor function in primary patient samples (new Figures 1B, and supplemental Figure S2C)
- new functional data on the ARTS mimetic A4 (new supplemental Figure 3H),
- new descriptive data on transcriptome upon XIAP knockdown (new supplemental Figure 4H), protein levels of IAPs (new supplemental Figures 1B and 1N) and signs of apoptosis (new Figure 4H), among others

With the help of the reviewer's comments and by carefully going through the entire manuscript again, we removed inconsistencies and improved data analysis and presentation as detailed below.

Referee #2 (Remarks for Author):

The manuscript by Carlet et al. shows that treatment with SMAC mimetics induces apoptosis in r/rALL cell lines in a synergistic fashion when used in combination with some chemotherapeutic agents. This action appears to be dependent on inhibition of XIAP. The manuscript contains interesting preliminary data. Indeed, while the data are provocative and point to potentially interesting clinical issues, my enthusiasm is weakened since, on a closer look, the analysis performed by the authors is, in most cases, an over-interpretation. Critical results have been obtained exclusively in cell lines and the clinical issues are, at this time, too theoretical for a journal as EMBO Molecular Medicine. In spite of that, it is important to recognize the enormous amount

of work included in this manuscript and the use of interesting and well-designed GEPDX models.

Authors' Response: We thank the reviewer for acknowledging our advanced molecular in vivo methods and the data richness of our manuscript.
After carefully going through data and Figures again and replacing certain Figures and statements, we now erased over-interpretation from the manuscript. We strengthened the translational part of our manuscript by adding data on additional primary sample.
We consider our data of clear and direct translational nature and highly suitable for EBMO Molecular Medicine as target journal – which preclinical model is closer to the patient than our PDX model? The PDX model combines the work on patient tumor cells with the in vivo setting and is thus considered highly translational.

Main concerns:

1. In the first part of the manuscript (Figures 1 and 2), the rationale on XIAP implication in ALL is not well explained and it is difficult to follow (Figures/supplementary data). Tests were mainly performed in cell lines and more experiments on primary cells are critically missing.

   Authors' Response:
   When starting the project, we were not expecting that XIAP would be the target for SM-drugs and started by excluding other, signaling pathways which were more likely mechanisms according to the literature. Thus, XIAP was not in the focus on Figures 1 and 2. We carefully revised Figures 1 and 2, and the related supplemental Figures for clarity and better readability. For primary cells, please see below at major point 3.

2. The authors seem to over-interpret the results included in the aforementioned figures. A clear example (among others) is the assessment of the intracellular apoptosis signaling activated by the SM-drug combination. Some data are analyzed in the Loucy cell line (caspase activation, independency from TNFa) other in the NALM-6 cell line (NFKb pathway analysis). The results are described in the Results section as a mix of the two cell lines and the conclusion is expressed as a general issue. Additionally, most of the conclusions are negative results without the corresponding positive controls.

   We undertook major efforts to erase inconsistencies and over-interpretations.
   We performed new experiments and complemented missing data sets and/or controls, resulting in new Figures 2A,B and S2C,D or improved Figures 2C,D,F. As a result, all critical experiments with important controls were now performed in both cell lines, Loucy and NALM-6 cells, yielding similar results; the only exception is NF*k*B signaling which was studied in Loucy cells only (Figure 2D-F). We now consistently present results from Loucy cells in the printed Figures 1 and 2 and data from NALM-6 cells in the respective supplement.

3. In these figures, only a panel including primary cells is included. This experiment is performed in a single patient (obviously without statistics), making the conclusion also an over-interpretation.

We added another patient sample to Figure 1B and show that the pro-apoptotic effects of the SM- drug combination is independent from TNF signaling in these cells (Figure S2C). Because patient cells are scarce, experiments could not be repeated several times explaining the lack of statistics.

4. The effect of the combination of LBW242 and other cytotoxic drugs is defined as synergistic (line 72). However, according to the explanation on the combination indexes included in the legend, it seems that only the combination LBW242 + Vincristine is in fact synergistic. The combination LBW242 + L-Asp appeared to be the addition of the two drugs, while LBW242 + Doxo is antagonistic. How do the authors explain this effect?
5. Data included in Supplementary Tables 1 and 2 are exactly the same. This raises doubts about the results.

Combined comment to points 4 and 5:
We apologize for certain copy paste errors in the supplemental Tables that we now corrected. Table S1 displays the data for Loucy cells. In Loucy cells, we observe a synergism of LBW not only with VCR but also Dexamethasone, while the combination of LBW242+L-Asp appears indeed additive. We clearly state in the revised manuscript that LBW242 is not capable to overcome
"*p53-mediated resistance of Loucy cells towards Doxorubicin*" (page 11).
Table S2 now correctly displays data for NALM-6 cells, where the majority of doses tested of the distinct LBW242-drug combinations are per definition synergistic. Accordingly and considering results from NALM-6 and Loucy cells, we state in the text that cell death was "*mostly synergistic*" (page 11).

6. In the line 59, the authors claim they continued to use the cell lines NALM-6 and Loucy because they were among the most resistant to LBW treatment. However, in Supplementary Fig 1A there are other cell lines that show similar levels of apoptosis, and also two cell lines that show more resistance to apoptosis (Molt-3 and Molt-4). Why were these cell lines not used in further experiments?

We now clarified in the text that we chose NALM-6 and Loucy based on the fact that these cells demonstrate a very low sensitivity towards both, SM and chemotherapy, which we define as r/r ALL, while Molt-3 and Molt-4 displayed no SM sensitivity at all (page 10).

7. In correlation with the values presented in Supplementary Tables 1 and 2 for the combination of LBW242 + Doxo, results depicted in Supplementary Figures G and K do not show increased sensitivity of these cell lines to the combined treatment, which does not correspond to the claim made in lines 69-70. Do the authors have an explanation for the difference in the response to LBW242 + Doxo and LBW242 + the other drugs tested?

We clearly state in the revised manuscript that LBW242 is not capable to overcome "*p53-mediated resistance of Loucy cells towards Doxorubicin*" (page 11). P53 might be mechanistically responsible for complete resistance against Doxorubicin, but not

the other drugs tested which do not activate DNA damage response. NALM-6 cells are sensitive to Doxo treatment, and addition of LBW242 increased cell death.

8. In line 86, the authors claim that both c-IAP1 and 2 were degraded by SM treatment. However, the blots ins Supplementary Figs 1S, T, and U show a different behavior between c-IAP 1 and 2. While the sentence seems appropriate for c-IAP1, c-IAP2 goes from being completely absent to being present and does not change depending on the cell type or SM used. How do the authors explain this issue?

Indeed, c-IAP1 and c-IAP2 show a different behavior, however, for both, cell death does not correlate with the presence or the absence of the respective protein. We clarified this in the text, pages 11-12.

9. LCL161 is used in Figures 1A and Supplementary Figure 1E, but it is only defined as a LBW242 analogue further in the text (line 68). It should be explained earlier.

We regret - LCL161 is now introduced earlier – page 10.

10. In Supplementary Fig 1O, is there an error in the Vincristine concentration shown above the blot? In Supplementary Fig 1N the concentrations used were 1 and 2.5 ng/ml, while in the blot it shows 1 and 0.25 ng/ml, which seems awkward also because it seems 0.25 ng/ml leads to higher levels of cleaved Caspase-8 than 1 ng/ml. A similar issue is observed in Supplementary Figure 1R and Figure 1F.

There was indeed an error in the labelling. The mistakes in Figures S1O, S1R and 1F are now corrected in revised Figures S1G, 1F and S1K respectively.

11. In Figure 2A, cell death seems reduced in presence of Nec-1 + LBW, which does not agree with what is stated in the text. Moreover, cell death seems to increase after Vincristine + Nec-1 treatment, which is also not expected. Are these comparisons statistically relevant? Any explanation? - In this panel, is the legend correct for the two bars on the right? Or the legend described the LBW (20µM) + TNF 20 ng/mL combination?

The reviewer is right, and we corrected the labeling of Figure 2A and supplemental Figure 2E-F; cell death is reduced only in the positive control, when cells are treated with the combination of LBW242 and TNFα. We apologize for this confusing mistake. Statistics have been added to the Figures. The effect of Nec-1 on Vincristine induced apoptosis is unexpected, but is in line with a previous report for leukemic cells (PMID: 22837689) and may account for the Nec-1 mediated-blockage of NF kappa B survival signaling induced by Vincristine.

12. In Fig 2B, what are the time points? This information is not indicated either in the legend or in the Figure.

5, 15 and 60 minutes, which is now indicated in revised Figure 2C.

13. In Fig 2C, how do the authors explain the increase in apoptotic cells by both the control and the NEMO siRNA?

The increase of apoptosis occurs due to the transfection procedure (electroporation) that stresses the cells according to our experience in contrast to untransfected cells. For direct comparison, we no longer show the “untransfected” control, but the KD with specific versus the scrambled siRNA in Figures 2B, D and F.

14. In line 107, the authors write that « NALM-6, SM alone or in combination with chemotherapy induced p100 processing that was "not sufficient to induce apoptosis". Even if it appeared that p100 processing happens specifically after SM treatment (Fig 2D), the results shown in Fig 2E do show that the same combination of VCR + LBW leads to more than 80% of apoptotic cells.

The reviewer is right, we clarified this point in the main text, page 13 as followed:

“Although SM alone or in combination with chemotherapy induced p100 processing (Figure 2E), RelB KD had no effect on apoptosis induced by SM alone or in combination with chemotherapy (Figure 2F), indicating that non-canonical NF-κB signaling is dispensable for cell death induction.”

15. This reviewer disagrees with the conclusion in lines 114-117. The only issue that can be drawn based on these experiments is that apoptosis caused by SM treatment appears to be independent on RIP1, NF-kB or TNFα.

We have removed the respective statement.

16. A statistical analysis should be included in the entire figure.

We have included a statistical analysis to all Figure subpanels, whenever appropriate.

17. In Supplementary Fig 2A, the combination of L-Asp + Prednisone leads to virtually 100% apoptotic cells, which is greatly decreased to less than 10% by adding Adalimumab. Do the authors have an explanation for this observation, which is clearly different from the combination VCR + Prednisone?

We have to apologize for this copy-paste error. The cells served as the positive control and were treated with LBW242+TNFα +/- Adalimumab; the mistake was corrected in revised supplemental Figure S2A.

18. In Figure 3A, as well as in Supplementary Figures 3B and D, the authors did not use the correct statistical test. There is a huge difference between the sizes of the samples in the control and AML/ALL groups. The Tukey's test assumes equal variance between the groups, and is dependent on both equal variance and similar group sizes for correct comparisons. The information described in the figure legends, as well as in the Methods section, does not describe any such procedure of correction. The reviewer strongly suggests a revision of these statistical tests.

We thank the reviewer for pointing this out. We consulted a mathematician, Vindi Jurinovic, who is a coauthor of the revised manuscript. Accordingly, we performed new statistical analysis and now use the Games-Howell post-hoc Test, which is independent of equal variance. This new analysis does not change the major outcome; XIAP but not cIAP1/2 is significantly upregulated in ALL.

19. In Figure 3B, are the comparisons of protein expression values for PBMC and PDX samples statistically significant? Additionally, the results of Supplementary Fig 3G do not look like the quantifications shown in Figure 3B. In fact, most of PDX samples (except ALL-50 and ALL-265) do not seem to have higher levels of XIAP (contrary to what is stated in the Results section).

We performed a new analysis and demonstrate in supplemental Figure S3G that the difference in expression values of XIAP protein in PBMC compared to ALL PDX is statistically significant. We want to point out that protein expression was determined by capillary protein immunoassay, not by conventional western blot. Details of the method and discrepancies to conventional western blotting is described in the method section (page 7), where we also highlight that

> *"due to very high sensitivity, equal loading is hard to achieve, but also not required, as protein amounts are calculated with high sensitivity and reliability"*

For this reason, normalization to the loading control is of utmost importance in this assay, and the graph shown in Figure 3B indicates relative expression of XIAP normalized to b-actin; this is now highlighted in the Figure legend.

20. a) In Supplementary Figure 3F, cIAP-1 shows a similar behavior to XIAP (as well as in other figures). The authors do not acknowledge or discuss the fact that some of the effects observed might also be linked to cIAP-1.
    b) It it known that SMAC mimetics are more specific towards cIAP-1 and -2, while ARTS mimetics are more specific to XIAP. It would be important to perform at least the most important experiments of this manuscript with ARTS mimetics as well.

    a) Important for the message of the paper and mechanistically relevant, cIAP-1 is differently regulated compared to XIAP after stimulation with drugs (Figure S1M) and upon XIAP knockdown (Figure 4C). This is highlighted at several points in the text.
    b) We thank the reviewer for the clever idea to include ARTS mimetics into the manuscript. We performed new experiments using the commercially available small molecule ARTS mimetic A4 and found that A4 sensitized r/rALL to chemotherapy, in analogy to SM (new Figure S3H). These data strengthen the main and clinically important message of our manuscript that inhibition of XIAP sensitizes r/rALL cells towards chemotherapy.

21. Line 406: "vain" instead "vein". "SCID" should be in capital letters.

Thanks, the typos have been corrected.

22. The authors should include a statistical assessment in Supplementary Figures 3A to E.

A statistical analysis is now included for Figures S3A to E; in accordance with the statement made in the text, the expression of cIAP1 and cIAP2 is statistically not significant between ALL patients and healthy controls.

23. As indicated above, the results obtained in the GEPDX models are interesting, original and they have also been well explained and designed. They deserve to be studied more deeply. Indeed, some key questions should be answered before concluding that effects observed were due to different XIAP expression or that there exists a yet unknown survival function for XIAP. For example, it is important: (i) to perform a molecular analysis that could allow the EMBO Molecular Medicine readers to understand the role of XIAP in the absence of direct apoptotic stimuli; (ii) to uncover a molecular tool supporting the idea that there exists a survival role of XIAP; and, (iii) to demonstrate, with molecular/biochemical data, the assertion "strong XIAP inhibition is required to unmask XIAPs essential function, while intermediate inhibition is not sufficient".

We thank the reviewer for acknowledging our GEPDX models as advanced technique used for the first time in the present manuscript.
We performed new experiments and now show that cells with strong XIAP knockdown undergo apoptosis associated with PARP cleavage (new Figure 4H) and are characterized by a unique transcriptomic profile (new suppl. Figure 4H).
(i) Figures 4D-G and 5A-D show in vivo data on XIAP knockdown in the absence of direct apoptotic stimuli, unraveling the novel finding that knockdown of XIAP alone exerts a lethal function in PDX ALL models in vivo. (ii) We show that GEPDX models represent appropriate molecular tools to reveal a survival role of XIAP (Figure 4 and 5). (iii) By separating cells with high and low expression of dsRED/XIAP knockdown via flow cytometry, we showed that knockdown strength and function are directly related (Figure 5B).

Referee #3:

This is a well-designed and generally clearly presented study with new information of interest.

Authors' Response: We thank the reviewer for acknowledging our study and its novel information.

I have the following comments that should be addressed:

1. The study presents data on T-and B-ALL cell lines and patients' samples as if this makes no difference which clearly goes against most thinking about the mechanisms perturbed in these different types of human ALL. At the very least, it would be important to acknowledge this at the outset and state clearly which diseases were being modelled in each set of experiments that form the main body of the paper, especially given the fact that only one or 2 cell lines or patient samples of each type were studied in any given set of experiments.

Authors' Response: We appreciate the reviewers concern. Interestingly and while the process of leukemogenesis is clearly different between both ALL subtypes, current treatment routine largely overlaps. In our small study, we found identical behavior in r/r B-ALL and T-ALL. We now clearly indicate where B-ALL and where

T-ALL cells were analyzed. To substantiate our findings, we further have added additional experiments using patient samples in revised Figures 1B and S2C.

2. On a similar note, more detail should be provided in the main manuscript on all primary samples (age, sex, diagnosis, ALL type, blast count, main genetic abnormalities, normal bone marrow source, etc.) ideally in a table.

New supplemental Table S3 now shows clinical data of the patients from whom PDX samples were derived. Information on the primary pediatric patient samples is included in the respective Figure legends.

3. No information is provided on how many times experiments were independently repeated. Are errors shown just for replicate cultures or mice within a representative single experiment, or between independent experiments?

In vitro, errors were calculated between independent experiments; *In vivo*, each mouse is considered independent. We now indicate this information in the legend of each Figure panel.

4. Please provide data on lentiviral gene transfer efficiencies.

As we aimed for low, at best a single lentiviral integration per cell, transduction efficiencies were optimized to be below 30%, as now stated on page 9.

5. Data with a single shRNA is not typically considered sufficient to rule out offtarget effects contributing to altered biological responses.

The reviewer is right and we selected 2 shRNA sequences in supplemental Figures 4AB; the second sequence, shXIAP.2, reproduced the data of shXIAP.1 in NALM 6 cells (supplemental Figures 4EF).

17th Jun 2021

Dear Dr. Jeremias,

Thank you for the submission of your new manuscript to EMBO Molecular Medicine, and please accept my apologies for the delay in getting back to you as one referee needed more time to complete his/her report.
We could unfortunately not secure all the referees who reviewed the first version of your manuscript, and only referee #2 (now referee #1) had reviewed the initial version. The second referee (referee #2) nevertheless reviewed your manuscript, the initial reports and your point-by-point letter.

As you will see from the reports below, while referee #1 acknowledges the efforts that were made to address the initial concerns, this referee also mentions substantial issues, and regrets that not all points from the initial review have been addressed. Referee #2, who did not review the initial version of your manuscript, is more positive, and asks for a few clarifications.

Addressing the reviewers concerns in full will be necessary for further considering the manuscript in our journal. In particular, I would encourage you to address all points initially raised by referee #2 (now referee #1), and to carefully check the manuscript for inaccuracies. Acceptance or rejection of the manuscript will depend on the completeness of your responses included in the next, final version of the manuscript. For this reason, and to save you from any frustrations in the end, I would strongly advise against returning an incomplete revision and would also understand your decision if you choose to rather seek rapid publication elsewhere at this stage.

Revised manuscripts should be submitted within three months of a request for revision, except under exceptional circumstances in which a short extension is obtained from the editor.

When submitting your revised manuscript, please carefully review the instructions that follow below. We perform an initial quality control of all revised manuscripts before re-review; failure to include requested items will delay the evaluation of your revision.

We require:

1) A .docx formatted version of the manuscript text (including legends for main figures, EV figures and tables). Please make sure that the changes are highlighted to be clearly visible.

2) Individual production quality figure files as .eps, .tif, .jpg (one file per figure). For guidance, download the 'Figure Guide PDF' (https://www.embopress.org/page/journal/17574684/authorguide#figureformat).

3) A .docx formatted letter INCLUDING the reviewers' reports and your detailed point-by-point responses to their comments. As part of the EMBO Press transparent editorial process, the point-by-point response is part of the Review Process File (RPF), which will be published alongside your paper.

4) A complete author checklist, which you can download from our author guidelines (https://www.embopress.org/page/journal/17574684/authorguide#submissionofrevisions). Please insert information in the checklist that is also reflected in the manuscript. The completed author checklist will also be part of the RPF.

5) It is mandatory to include a 'Data Availability' section after the Materials and Methods. Before submitting your revision, primary datasets produced in this study need to be deposited in an appropriate public database, and the accession numbers and database listed under 'Data Availability'. Please remember to provide a reviewer password if the datasets are not yet public (see https://www.embopress.org/page/journal/17574684/authorguide#dataavailability).

In case you have no data that requires deposition in a public database, please state so in this section. Note that the Data Availability Section is restricted to new primary data that are part of this study.

6) For data quantification: please specify the name of the statistical test used to generate error bars and P values, the number (n) of independent experiments (specify technical or biological replicates) underlying each data point and the test used to calculate p-values in each figure legend. The figure legends should contain a basic description of n, P and the test applied. Graphs must include a description of the bars and the error bars (s.d., s.e.m.).

7) We would also encourage you to include the source data for figure panels that show essential data. Numerical data should be provided as individual .xls or .csv files (including a tab describing the data). For blots or microscopy, uncropped images should be submitted (using a zip archive if multiple images need to be supplied for one panel). Additional information on source data and instruction on how to label the files are available at
.

8) Our journal encourages inclusion of *data citations in the reference list* to directly cite datasets that were re-used and obtained from public databases. Data citations in the article text are distinct from normal bibliographical citations and should directly link to the database records from which the data can be accessed. In the main text, data citations are formatted as follows: "Data ref: Smith et al, 2001" or "Data ref: NCBI Sequence Read Archive PRJNA342805, 2017". In the Reference list, data citations must be labeled with "[DATASET]". A data reference must provide the database name, accession number/identifiers and a resolvable link to the landing page from which the data can be accessed at the end of the reference. Further instructions are available at .

9) We replaced Supplementary Information with Expanded View (EV) Figures and Tables that are collapsible/expandable online. A maximum of 5 EV Figures can be typeset. EV Figures should be cited as 'Figure EV1, Figure EV2" etc... in the text and their respective legends should be included in the main text after the legends of regular figures.
- For the figures that you do NOT wish to display as Expanded View figures, they should be bundled together with their legends in a single PDF file called *Appendix*, which should start with a short Table of Content. Appendix figures should be referred to in the main text as: "Appendix Figure S1, Appendix Figure S2" etc.
- Additional Tables/Datasets should be labeled and referred to as Table EV1, Dataset EV1, etc. Legends have to be provided in a separate tab in case of .xls files. Alternatively, the legend can be supplied as a separate text file (README) and zipped together with the Table/Dataset file.
See detailed instructions here:
.

10) The paper explained: EMBO Molecular Medicine articles are accompanied by a summary of the articles to emphasize the major findings in the paper and their medical implications for the non-specialist reader. Please provide a draft summary of your article highlighting
- the medical issue you are addressing,
- the results obtained and
- their clinical impact.

This may be edited to ensure that readers understand the significance and context of the research. Please refer to any of our published articles for an example.

11) For more information: There is space at the end of each article to list relevant web links for further consultation by our readers. Could you identify some relevant ones and provide such information as well? Some examples are patient associations, relevant databases, OMIM/proteins/genes links, author's websites, etc...

12) A Conflict of Interest statement should be provided in the main text.

13) Every published paper now includes a 'Synopsis' to further enhance discoverability. Synopses are displayed on the journal webpage and are freely accessible to all readers. They include a short stand first (maximum of 300 characters, including space) as well as 2-5 one-sentences bullet points that summarizes the paper. Please write the bullet points to summarize the key NEW findings. They should be designed to be complementary to the abstract - i.e. not repeat the same text. We encourage inclusion of key acronyms and quantitative information (maximum of 30 words / bullet point). Please use the passive voice. Please attach these in a separate file or send them by email, we will incorporate them accordingly.

Please also suggest a striking image or visual abstract to illustrate your article as a PNG file 550 px wide x 300-600 px high.

14) As part of the EMBO Publications transparent editorial process initiative (see our Editorial at http://embomolmed.embopress.org/content/2/9/329), EMBO Molecular Medicine will publish online a Review Process File (RPF) to accompany accepted manuscripts.
In the event of acceptance, this file will be published in conjunction with your paper and will include the anonymous referee reports, your point-by-point response and all pertinent correspondence relating to the manuscript. Let us know whether you agree with the publication of the RPF and as here, if you want to remove or not any figures from it prior to publication.
Please note that the Authors checklist will be published at the end of the RPF.

EMBO Molecular Medicine has a "scooping protection" policy, whereby similar findings that are published by others during review or revision are not a criterion for rejection. Should you decide to submit a revised version, I do ask that you get in touch after three months if you have not completed it, to update us on the status.

I look forward to receiving your revised manuscript.

With kind regards,

Lise Roth

Lise Roth, PhD
Editor
EMBO Molecular Medicine

***** Reviewer's comments *****

Referee #1 (Comments on Novelty/Model System for Author):

Acknowledging the enormous effort of the authors, errors are still detected in the Figures and Results sections that would be too long to enumerate. Some of them: differences in apoptosis detected in the new T-ALL patient included in the MS when treated with vincristine/LBM (e.g. 60% apoptosis in the combined treatment vincristine 1 ng/ml and 20 µM LBW in Figure 1B and 25% with the same treatment in Supplemental Figure 2), in this sample of T-ALL patient, very surprisingly, treatment with 1 ng/ml vincristine induced more apoptosis than treatment with 5 ng/ml vincristine (Figure 1B), lack of information on the two ALL patients used in the in vitro tests, lack of statistics in many figures (e.g. Figure 1A, Supplemental Figure 1C...), in others a statistically significant data appears in the figure legend (e.g. p 0.05), without any statistics (*) in the figure (e.g. Supplemental Figure 1C), lack of quantification of immunoblots with an over-interpretation of the results (e.g., Figure 1B), lack of definition of reagents (LFT122 ?), a rationale that in my view is not clear in the choice of the LOUCY and NALM-6 cell lines, a unconvinced explanation of p53 and the non-response of the LOUCY cell line to Doxorubicin (Supplemental Figure 1F). Dexamethasone and Vincristin are not p53-dependent?.... more than 300 articles in Medline seem to say the opposite). And these are just few points.

With all due respect, it seems to me that it is not the reviewers who must correct a manuscript, but the authors who must generate sufficiently solid and error-free data to transmit it to the scientific community.

Referee #1 (Remarks for Author):

The new version of the manuscript is obviously much better than the first one in which the reviewers raised almost 40 questions, many of them to correct inaccuracies/errors in the tables, figures, statistics, and interpretation of the results. The authors have amended a large part of them, although many of their answers, in my point of view, are still over-interpretations of their results or simply assertions without a solid basis. I believe that the authors' message is possibly relevant and original and even of medical interest. However, the inaccuracies again in this second submission (too frequent to list) make me think that this article does not have sufficient standards to be published in a top-tiered journal such as EMBO Molecular Medicine.

Referee #2 (Remarks for Author):

I had a chance to go through the reviewers' comments and the responses.
I think that the manuscript should be given a chance as the findings are novel and of interest for the ALL leukemia field. 2 out of the 3 reviewers are positive or have mainly a few discussion points or suggestions. Indeed, reviewer #2 had some valid comments. Nevertheless, the authors have largely addressed those concerns. One comment I have is that the authors should clarify in the manuscript that LOUCY is not a classic T-ALL cell line but it belongs to the early T-cell progenitor ALL (ETP-ALL) category, an aggressive type of ALL originating from a (very early) T cell progenitor different than the classic T-ALL. They should also discuss whether the patient samples used in their study belong to the same subtype and if possible identify XIAP levels in patients of this particular disease subtype.

Author Response to Reviewers

******* Reviewer's comments *******

Referee #1 (Comments on Novelty/Model System for Author):

Acknowledging the enormous effort of the authors, errors are still detected in the Figures and Results sections that would be too long to enumerate.

We thank the reviewer for acknowledging the considerable effort that we put into revising the manuscript and for her/his constant efforts to point out incomplete data. Based on the reviewer´s suggestions, we now improved data presentation throughout the manscript.

Some of them:

1. differences in apoptosis detected in the new T-ALL patient included in the MS when treated with vincristine/LBM (e.g. 60% apoptosis in the combined treatment vincristine 1 ng/ml and 20 µM LBW in Figure 1B and 25% with the same treatment in Supplemental Figure 2),

   The samples in Figure 1B and Supplementary Figure 2 (now labeled Figure EV1) were indeed from 2 different T-ALL patients showing different sensitivity towards VCR and LBW242. We now included patient's characteristics of all ex vivo handled samples in **new Expanded View Table EV1**.

2. in this sample of T-ALL patient, very surprisingly, treatment with 1 ng/ml vincristine induced more apoptosis than treatment with 5 ng/ml vincristine (Figure 1B),

   Indeed, in this primary sample, 1 ng/ml and 5 ng/ml Vincristine induced 59% and 56% apoptosis, respectively, which we interpret as saturation of the anti-tumor effect of the drug. For a dose response, lower doses should be used which was unfortunately unfeasible due to the scarcity of the primary tumor cells.

3. lack of information on the two ALL patients used in the in vitro tests,

   We detailed all patient information in **new Expanded View Table EV1**.

4. lack of statistics in many figures (e.g. Figure 1A, Supplemental Figure 1C...), in others a statistically significant data appears in the figure legend (e.g. p 0.05), without any statistics (*) in the figure (e.g. Supplemental Figure 1C),

   We thank the reviewer for bringing up this point; we complemented and improved the depiction of statistical analyses in all appropriate Figures and Legends.

5. lack of quantification of immunoblots with an over-interpretation of the results (e.g., Figure 1B),

   Quantifications for immunoblots have been included for all relevant blots in main and supplemental Figures. Uncropped western blot images and raw data of

capillary immunoassays of all main figures, together with their quantification have been included in the **new Source Data file**.

6. lack of definition of reagents (LFT122 ?)

   LFT122 has been removed from the manuscript.

7. a rationale that in my view is not clear in the choice of the LOUCY and NALM-6 cell lines,

   Criteria for the choice of cell lines included
   i) relative resistance to chemotherapeutic drugs, which was the prerequisite to model r/r ALL
   ii) relative resistance to SM,
   iii) representation of both, B-ALL (NALM-6) and T-ALL (LOUCY) disease

   These points are now described on page 4.

   "We selected LOUCY cells for further analysis given the known resistance of the early immature T-ALL subtype represented by the cell line to chemotherapeutic agents (Anderson, Harrold et al., 2014); NALM-6 cells which showed similar response to SM than LOUCY were selected as a model for B-ALL disease…"

8. a unconvinced explanation of p53 and the non-response of the LOUCY cell line to Doxorubicin (Supplemental Figure 1F). Dexamethasone and Vincristin are not p53-dependent?.... more than 300 articles in Medline seem to say the opposite).

   LOUCY cells are known to poorly respond to chemotherapeutic agents, however, we and others (Anderson, Harrold et al., 2014) observed that response to vincristine was comparable to other leukemia cells. In contrast to doxorubicin which induces DNA damage and requires p53 to induce apoptosis (Salih, Elena et al., 2020), vincristine as a microtubule destabilizing agent classically relies on SAPK signaling (JNK, p38) to excerpt its antitumor effects.
   We had previously shown that in acute leukemia, p53 activation even diminishes response to vincristine (Ehrhardt, Schrembs et al., 2011). Because we found LOUCY cells highly resistant against Doxorubicin but not vincristine, and Loucy cells reportedly carry a p53 mutation (Prokocimer, Peller et al., 1998), but NALM-6 cells are p53 wt (Salih et al., 2020) we hypothesize that p53 is responsible for the observed phenotype. Due to the reviewer´s comment, we now tuned down our wording and added references; page 5:

   "*Resistance of LOUCY cells might eventually be attributed to loss of function of p53, a known mediator of Doxorubicin-induced apoptosis (Prokocimer, Peller et al., 1998, Salih, Elena et al., 2020)*"

And these are just few points. With all due respect, it seems to me that it is not the reviewers who must correct a manuscript, but the authors who must generate sufficiently solid and error-free data to transmit it to the scientific community.

We clearly acknowledge the reviewer's precision, from which we can learn enormously. We regret that data were presented incompletely and did our best to improve data depiction. We want to underline that data were presented incompletely,

but not incorrectly. We carefully checked the entire manuscript again and further improved data presentation in several sub-panels.

Referee #1 (Remarks for Author):

The new version of the manuscript is obviously much better than the first one in which the reviewers raised almost 40 questions, many of them to correct inaccuracies/errors in the tables, figures, statistics, and interpretation of the results. The authors have amended a large part of them, although many of their answers, in my point of view, are still over-interpretations of their results or simply assertions without a solid basis. I believe that the authors' message is possibly relevant and original and even of medical interest. However, the inaccuracies again in this second submission (too frequent to list) make me think that this article does not have sufficient standards to be published in a top-tiered journal such as EMBO Molecular Medicine.

We thank the reviewer to appreciate the relevance, originality, novelty and medical interest of our study.

Referee #2 (Remarks for Author):

I had a chance to go through the reviewers' comments and the responses. I think that the manuscript should be given a chance as the findings are novel and of interest for the ALL leukemia field. 2 out of the 3 reviewers are positive or have mainly a few discussion points or suggestions. Indeed, reviewer #2 had some valid comments. Nevertheless, the authors have largely addressed those concerns.

We thank the reviewer for her/his valuable suggestions and for the opportunity to further correct and clarify distinct points. We have addressed all points as outlined below which has further improved the manuscript.

One comment I have is that the authors should clarify in the manuscript that LOUCY is not a classic T-ALL cell line but it belongs to the early T-cell progenitor ALL (ETP-ALL) category, an aggressive type of ALL originating from a (very early) T cell progenitor different than the classic T-ALL.

We thank the reviewer for this helpful suggestion and included this information along with the corresponding citations on pages 4 and 17.

> *"We selected LOUCY cells for further analysis, as they belong to the early immature ETP T-ALL subtype, known for its resistance against chemotherapeutic agents (Anderson et al., 2014)"*

> *"Cell lines, including LOUCY (human T-ALL, resembling the early T-cell precursor ETP-ALL subtype (Ben-Bassat, Shlomai et al., 1990, Zhang, Ding et al., 2012)) and NALM-6 (human pre B-ALL, resembling DUX4-rearranged B-ALL (Hurwitz, Hozier et al., 1979, Yasuda, Tsuzuki et al., 2016)) were purchased from DSMZ (German collection of microorganisms and cells, Braunschweig, Germany)"*

They should also discuss whether the patient samples used in their study belong to the same subtype

In our study, indeed 2 samples belong to the ETP T-ALL subtype, namely ALL-2 and ALL-3 (primary samples, childhood ALL, initial diagnosis). This detail is now presented in the patients' information in **Expanded View Table EV1.**

and if possible identify XIAP levels in patients of this particular disease subtype.

Following the interesting suggestion by the reviewer, we analyzed 2 published datasets and one own data for XIAP expression in the ETP-ALL subtype. With T-ALL samples, we used the 35 ETP related genes described in https://www.ncbi.nlm.nih.gov/pmc/articles/PMC2840241/ (Coustan-Smith, Mullighan et al., 2009) to create an ETP cluster in each data set. XIAP expression was compared between the ETP cluster and the remaining T-ALL samples.

Data set 1: An Illumina array data set from 53 adult T-ALL patients (Van Vlierberghe, Ambesi-Impiombato et al., 2013) was obtained from GEO (accession number GSE42328), where two probesets corresponded to the XIAP gene: ILMN_1815361 and ILMN_3203615. The expression of both probesets was slightly elevated in the ETP-cluster, however, the difference did not reach statistical significance (p = 0.31 and p = 0.10, respectively).

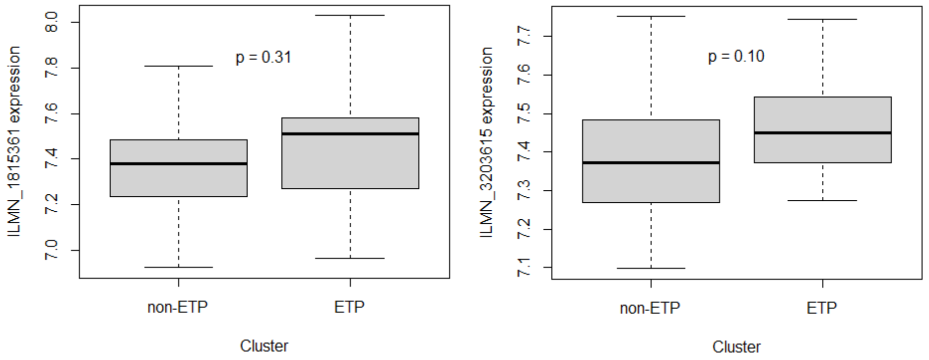

Data set 2: An Affymetrix data set consisting of 55 diagnostic T-ALL samples from the manuscript of Coustant-Smith (Coustan-Smith et al., 2009) was obtained from GEO (accession number GSE8879). This data set contains two XIAP probesets: 206536_s_at and 206537_at. The first probeset was slightly lower expressed in ETP-ALL, but the difference was not significant (p = 0.45), while the expression of the second probeset was higher in ETP-ALL (p = 0.087).

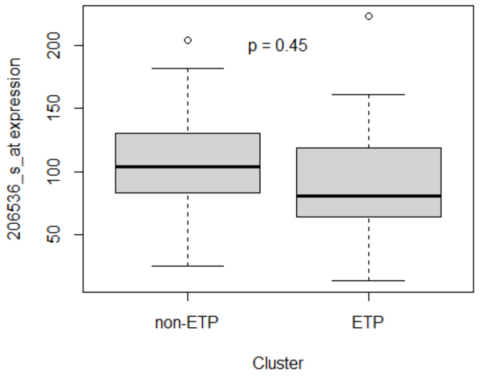

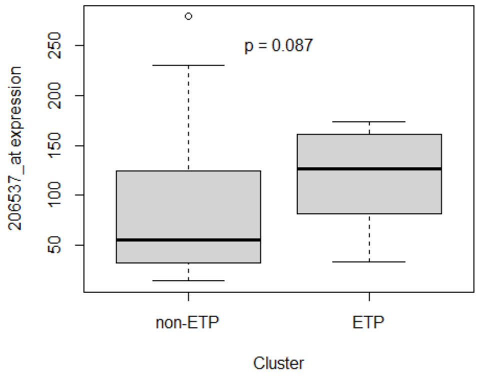

Data set 3: In our own Affymetrix microarray data set consisting of 84 patients with T-ALL (Herold, Jurinovic et al., 2018), XIAP showed significantly lower expression in the ETP cluster (p = 0.0039).

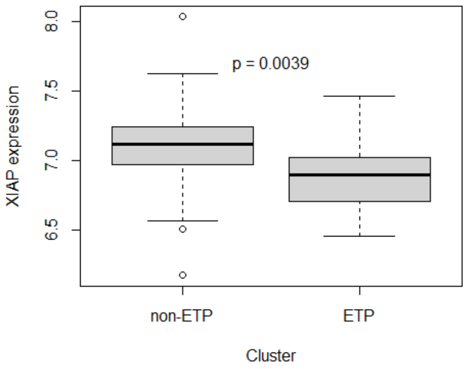

As the results of the three datasets are controversial, we did not include them into the manuscript; we are happy to do so at the reviewer´s discretion.

References

Anderson NM, Harrold I, Mansour MR, Sanda T, McKeown M, Nagykary N, Bradner JE, Lan Zhang G, Look AT, Feng H (2014) BCL2-specific inhibitor ABT-199 synergizes strongly with cytarabine against the early immature LOUCY cell line but not more-differentiated T-ALL cell lines. *Leukemia* 28: 1145-1148

Ben-Bassat H, Shlomai Z, Kohn G, Prokocimer M (1990) Establishment of a human T-acute lymphoblastic leukemia cell line with a (16;20) chromosome translocation. *Cancer genetics and cytogenetics* 49: 241-8

Coustan-Smith E, Mullighan CG, Onciu M, Behm FG, Raimondi SC, Pei D, Cheng C, Su X, Rubnitz JE, Basso G, Biondi A, Pui CH, Downing JR, Campana D (2009) Early T-cell precursor leukaemia: a subtype of very high-risk acute lymphoblastic leukaemia. *Lancet Oncol* 10: 147-56

Ehrhardt H, Schrembs D, Moritz C, Wachter F, Haldar S, Graubner U, Nathrath M, Jeremias I (2011) Optimized anti-tumor effects of anthracyclines plus Vinca alkaloids using a novel, mechanism-based application schedule. *Blood* 118: 6123-31

Herold T, Jurinovic V, Batcha AMN, Bamopoulos SA, Rothenberg-Thurley M, Ksienzyk B, Hartmann L, Greif PA, Phillippou-Massier J, Krebs S, Blum H, Amler S, Schneider S, Konstandin N, Sauerland MC, Gorlich D, Berdel WE, Wormann BJ, Tischer J, Subklewe M et al. (2018) A 29-gene and cytogenetic score for the prediction of resistance to induction treatment in acute myeloid leukemia. *Haematologica* 103: 456-465

Hurwitz R, Hozier J, LeBien T, Minowada J, Gajl-Peczalska K, Kubonishi I, Kersey J (1979) Characterization of a leukemic cell line of the pre-B phenotype. *Int J Cancer* 23: 174-80

Prokocimer M, Peller S, Ben-Bassat H, Goldfinger N, Rotter V (1998) P53 gene mutation in a T-acute lymphoblastic leukemia cell line (loucy) with t(16:20) and 5q- chromosomal aberrations. *Leuk Lymphoma* 29: 607-11

Salih D, Elena B, Qian S, Stephanie H, Eugen T, Cornelia E, Martin E, Rupert H, Geertruy te K, Lisa W, Stephan S, Galina S, Klaus-Michael D, Lüder Hinrich M (2020) Therapeutic targeting of mutant p53 in pediatric acute lymphoblastic leukemia. *Haematologica* 105: 170-181

Van Vlierberghe P, Ambesi-Impiombato A, De Keersmaecker K, Hadler M, Paietta E, Tallman MS, Rowe JM, Forne C, Rue M, Ferrando AA (2013) Prognostic relevance of integrated genetic profiling in adult T-cell acute lymphoblastic leukemia. *Blood* 122: 74-82

Yasuda T, Tsuzuki S, Kawazu M, Hayakawa F, Kojima S, Ueno T, Imoto N, Kohsaka S, Kunita A, Doi K, Sakura T, Yujiri T, Kondo E, Fujimaki K, Ueda Y, Aoyama Y, Ohtake S, Takita J, Sai E, Taniwaki M et al. (2016) Recurrent DUX4 fusions in B cell acute lymphoblastic leukemia of adolescents and young adults. *Nature genetics* 48: 569-74

Zhang J, Ding L, Holmfeldt L, Wu G, Heatley SL, Payne-Turner D, Easton J, Chen X, Wang J, Rusch M, Lu C, Chen SC, Wei L, Collins-Underwood JR, Ma J, Roberts KG, Pounds SB, Ulyanov A, Becksfort J, Gupta P et al. (2012) The genetic basis of early T-cell precursor acute lymphoblastic leukaemia. *Nature* 481: 157-63

20th Jan 2022

Dear Prof. Jeremias,

Thank you for the submission of your revised manuscript to EMBO Molecular Medicine, and please accept my apologies for the delay in getting back to you, as the referees needed more time to complete their reviews during this busy time of the year. We have now received feedback from the three referees who re-reviewed your manuscript. Referee #2 reviewed the previous version of your manuscript, whereas referees #3 and #4 were new referees.
As you will see from the reports below, while referee #2 is overall satisfied with the revised manuscript, referees #3 and #4 acknowledge the interest of the study, but nevertheless raise a number of important concerns that should be addressed before further consideration.
Given that the manuscript already underwent several rounds of reviews, and after discussion with my colleagues and cross-commenting with the referees, we would like you to address in the text or experimentally where needed the remaining concerns from the referees, with the exception of point 3 from referee #3 (in vivo experiments). The addition of in vivo survival analysis comparing control and shXIAP would enhance the impact of the findings, nevertheless we understand that these are major experiments, and therefore we will rather ask that you carefully balance the interpretation of the competitive transplant data and discuss the need for a survival analysis for translation in the manuscript text. Please be aware that this will be the last chance for you to address the referees' concerns.

Additionally, please also address the following editorial issues:
- Author contributions: please differentiate between Tobias Herold and Thomas Hoffman. Georg Eschenburg is missing.
- Conflict of interest: We updated our journal's competing interests policy in January 2022 and request authors to consider both actual and perceived competing interests. Please review the policy https://www.embopress.org/competing-interests and update your competing interests if necessary.
- Funding: Please add the DFG proposal MA 1876/13-1 in the submission system.
- Please update the reference format: 10 authors should be listed before et al.
- Please add "The Paper Explained" section: EMBO Molecular Medicine articles are accompanied by a summary of the articles to emphasize the major findings in the paper and their medical implications for the non-specialist reader. Please provide a draft summary of your article highlighting
- the medical issue you are addressing,
- the results obtained and
- their clinical impact.
This may be edited to ensure that readers understand the significance and context of the research. Please refer to any of our published articles for an example.
- Every published paper now includes a 'Synopsis' to further enhance discoverability. Synopses are displayed on the journal webpage and are freely accessible to all readers. They include a short stand first (maximum of 300 characters, including space) as well as 2-5 one-sentences bullet points that summarizes the paper. Please write the bullet points to summarize the key NEW findings. They should be designed to be complementary to the abstract - i.e. not repeat the same text. We encourage inclusion of key acronyms and quantitative information (maximum of 30 words / bullet point). Please use the passive voice. Please attach these in a separate file or send them by email, we will incorporate them accordingly.
Please also suggest a striking image or visual abstract to illustrate your article as a PNG file 550 px wide x 300-600 px high.
- Thank you for providing Source Data. Please upload them as one file per figure.
- As part of the EMBO Publications transparent editorial process initiative (see our Editorial at http://embomolmed.embopress.org/content/2/9/329), EMBO Molecular Medicine will publish online a Review Process File (RPF) to accompany accepted manuscripts.
This file will be published in conjunction with your paper and will include the anonymous referee reports, your point-by-point response and all pertinent correspondence relating to the manuscript. Let us know whether you agree with the publication of the RPF and as here, if you want to remove or not any figures from it prior to publication.
Please note that the Authors checklist will be published at the end of the RPF.

I look forward to receiving your revised manuscript.

With kind regards,

Lise Roth

Lise Roth, PhD
Editor
EMBO Molecular Medicine

***** Reviewer's comments *****

Referee #2 (Comments on Novelty/Model System for Author):

the manuscript includes a variety of technologies to support the authors' claims and proper statistical analysis.

Referee #2 (Remarks for Author):

The authors have addressed my comments and-largely-the comments of the other authors. I agree with not including the current data analysis for XIAP mRNA expression in this manuscript. The authors can instead briefly discuss this finding and refer to a future study to further identify the exact XIAP levels. Ideally, RNA-seq data from studies such as Zhang et al., Nature, 2012 (https://pubmed.ncbi.nlm.nih.gov/22237106/), should be consulted.

Referee #3 (Comments on Novelty/Model System for Author):

The in vivo model is unconventional and survival should be the major endpoint of the in vivo studies.

Referee #3 (Remarks for Author):

Carlet et al. describe a role for XIAP in drug-resistant B and T-ALL cells. Overall, the findings are of moderate significance in identifying a specific, and potentially translatable mechanism to overcome disease relapse.

Overall comments:
The manuscript clearly demonstrates a role for XIAP in ALL based on both the pharmacologic and genetic (i.e., knock-down studies). However, the impact is limited by the majority of studies in a limited number of ALL cell lines, an unclear relationship between XIAP expression and cell survival and drug resistance both in cell lines and clinical specimens, and some inconsistencies from study to study.

Specific comments:
1. It is not clear how the authors conclude that there is no relationship between responses to SMs in Figure 1A and IAP expression as there is no quantification of the western blot in Figure S1B that I can find in documents provided. It would be interesting to know whether the expression level of XIAP correlates with the ability of SMs to sensitizes ALL cells to chemotherapy.
2.There is variation in the baseline effect of the vincristine from experiment to experiment, for example in Figure 2, is confusing, and given this variability it is not clear how the replicates are so similar (providing small error bars) within a specific experiment.
3. It is difficult to interpret the in vivo studies that utilize a mixture of cells with different expression levels of XIAP. It would be more appropriate to carry out conventional studies using a single lines and examining tumor burden following chemotherapeutic treatment rather than comparative levels of cells. Using sufficient sample sizes should be able to overcome mouse-to-mouse variability that is provided as a rationale for these studies. Furthermore, survival analyses would provide the greatest translational relevance.
4. If XIAP expression levels are associated with drug resistance, then it would be informative to examine the correlation between expression levels and successful remission induction in clinical specimens.
5. It is not clear whether the specimens in Figure S2A examining newly diagnosed and relapsed are from the same patients, and it would be interesting to know if XIAP expression changes with disease progression.
6. The use of "normal" PBMCs as a comparator for the XIAP expression studies is not relevant and studies comparing expression with either B or T cell precursors would be more meaningful.

Referee #4 (Comments on Novelty/Model System for Author):

All categories rated have the potential to improve to "High" with moderate revision as indicated.

Referee #4 (Remarks for Author):

Carlet et al. XIAP targeting in ALL

The manuscript describes experiments to exploit increased expression of IAP proteins in ALL by treating with SMAC mimetic (SM) compounds in combination with classical chemotherapeutics. Using cell lines LOUCY, NALM-6, and patient-donated ALL cells, results show combination efficacy of the SM compounds BV6, LCL and LBW when co-administered with vincristine,

doxorubicin, and prednisone. This effect appears to be independent of the function of NFkB and RIPK1 and is characterized by an apoptosis-associated PARP cleavage pattern. Data show that SM compounds reduce cIAP-1 and cIAP-2 without triggering apoptosis, suggesting additional pro-apoptotic activities, and thus XIAP is investigated through pharmacologic inhibitors, knockdown, and over-expression approaches. Nearly complete XIAP knockdown was associated with spontaneous apoptosis in NALM-6 cells in vitro. XIAP knockdown resulted in a growth disadvantage using NALM-6 and patient-donated relapsed ALL cells in competitive transplantation experiments. The authors' conclusion is that XIAP has a yet-to-be-identified, essential function in ALL cells, that may be targetable.

Major Conclusions and General Comments
1. SMAC inhibitors in combination with classical chemotherapeutics are effective for treatment of ALL in vitro.
a. Previous reports supporting a similar conclusion should be better presented in the Introduction. Results consistent and different from these previous reports should be discussed directly.
2. XIAP mediates controls apoptosis responses in ALL, independent of a role for cIAP-1/2.
a. Results dissociate decreased cIAP protein levels from the induction of apoptosis (Appendix Figure S1). Interestingly, XIAP protein levels remain overall unchanged. How these results differ from the reduction of XIAP in response to treatment of ALL cells with Smac mimetic Birinipant (Richmond et al. Cancer Research 2016) should be discussed. While the results support that reduced cIAPs are not sufficient to induce apoptosis, it remains possible that cIAP reduction is a necessary molecular event for apoptosis initiation. These possibilities should be resolved experimentally, or the conclusions need to acknowledge the possible alternative possible interpretations.
3. XIAP regulates ALL apoptosis in vitro and leukemogenesis in vivo.
a. The conclusion is potentially strongly supported by competitive transplantation experiments that were well controlled and that potentially demonstrate the role of XIAP in ALL apoptosis control (Figures 4-5). However, the specificity of the XIAP knockdown needs to be validated at least in vitro through reconstitution of XIAP using shRNA-resistant re-expression constructs.
4. Manuscript revision.
a. Some comments from previous review require further revision, as noted.
5. Overall evaluation.
a. The manuscript has the potential to advance the possibility of targeting XIAP for ALL therapy, with revisions to strengthen major conclusions and address minor issues as indicated.

Specific comments
1. The presentation of Figure S1A is confusing. It is not clear why MOLT4 and MOLT3 are not pursued. (Manuscript revision).
2. In Figure EV1C-D, a condition testing the efficacy of anti-TNFa neutralizing Ab adalimumab in TNFa-treated cells is necessary to support the stated conclusion that SM LBW is functioning in a TNFa-independent fashion in the cells tested. Please check: the text references appear to indicate that EV1C will present ALL samples when in fact it shows NALM-6 cells, and vice versa for EV1D.
3. Figure 2D, a positive control would be required to support the conclusion of NEMO-independent apoptosis in response to SM, considering the incomplete knockdown of NEMO (62%). Nevertheless, what the experiment would contribute to the analysis, as the expected effect of NEMO knockdown would be increased apoptosis (if any effect is observed).
4. Figure EV2. Please indicate statistical significance (or not) in the data shown in panels C and E. (Manuscript revision).
5. Appendix Figure S2, there is not a control that demonstrates that ARTS A4 is functioning in an XIAP-dependent fashion.
6. Appendix Figure S3A: The legend and labeling of the figure does not sufficiently present the system being used. The first two rows of data are using which shRNA(s)? The GFP reporter shown in the data is not described in the legend. The legend indicates that the "expression of the dTomato is reduced," but this description does not match the data.

Point by Point response

Carlet et al., "*X-linked inhibitor of apoptosis protein represents a promising therapeutic target for relapsed/refractory ALL*"

We thank the Referees for their positive evaluation and helpful comments to improve our manuscript. We have addressed all points raised by the reviewers and revised the manuscript accordingly. In particular, we

- performed new experiments and added 4 new Figure panels; we performed molecular reconstitution of XIAP (new Figure 4I), new Western Blots (new Appendix Figures S2C and S3I) as well as apoptosis analysis (Expanded View Figure EV1D), provided together with respective new source files.
- re-analyzed own data and performed data mining of publicly available data (2 Figures for review only)
- clarified remaining points in the manuscript text.

A detailed description of the changes in the manuscript is provided below.

Referee #2 (Comments on Novelty/Model System for Author):
the manuscript includes a variety of technologies to support the authors' claims and proper statistical analysis.

<u>Referee #2 (Remarks for Author):</u>

The authors have addressed my comments and – largely – the comments of the other authors. I agree with not including the current data analysis for XIAP mRNA expression in this manuscript. The authors can instead briefly discuss this finding and refer to a future study to further identify the exact XIAP levels. Ideally, RNA-seq data from studies such as Zhang et al., Nature, 2012 (https://pubmed.ncbi.nlm.nih.gov/22237106/), should be consulted.

We thank the reviewer for this suggestion. We have now included a discussion on the need to determine the subtypes that would most benefit from treatment where we included a statement on ETP ALL.

Changes made in the manuscript:
Discussion, line 351ff:
"Determining the patient sub-groups that might benefit most from XIAP-directed therapies either as single agents or in combination treatments remain elusive. [.….] As gene expression signatures were suggested to predict response to SM in aggressive BCP-ALL subtypes such as Ph-like ALL (Richmond et al., 2016; Zinngrebe et al., 2020), it will be interesting to determine whether the chemoresistant ETP-ALL can be sensitized to treatment and whether gene expression signatures can predict response in this aggressive subtype (Anderson et al., 2014; Zhang et al, 2012)."

Referee #3 (Comments on Novelty/Model System for Author):
The in vivo model is unconventional and survival should be the major endpoint of the in vivo studies.

Competitive in vivo approaches have been wildly used as they increase sensitivity and reliability, while sparing resources, e.g., in mouse studies on normal hematopoiesis (Eaves, 2015; Maetzig et al, 2017). We had recently adapted such assays to PDX leukemia models, as they allow comparing cell populations, which only differ in their genetic manipulation, under the exact identical conditions in the same mouse (Carlet et al, 2021; Liu et al, 2020).

Changes made in the manuscript:
Discussion, line 319ff

- Eaves CJ (2015) Hematopoietic stem cells: concepts, definitions, and the new reality. *Blood* 125: 2605-2613
- Maetzig T, *et al* (2017) Lentiviral Fluorescent Genetic Barcoding for Multiplex Fate Tracking of Leukemic Cells. *Mol Ther Methods Clin Dev* 6: 54-65
- Carlet M, *et al* (2021) In vivo inducible reverse genetics in patients' tumors to identify individual therapeutic targets. *Nat Commun* 12: 5655
- Liu WH, *et al* (2020) Inducible transgene expression in PDX models in vivo identifies KLF4 as a therapeutic target for B-ALL. *Biomark Res* 8: 46

Referee #3 (Remarks for Author):

Carlet et al. describe a role for XIAP in drug-resistant B and T-ALL cells. Overall, the findings are of moderate significance in identifying a specific, and potentially translatable mechanism to overcome disease relapse.
Overall comments:
The manuscript clearly demonstrates a role for XIAP in ALL based on both the pharmacologic and genetic (i.e., knock-down studies). However, the impact is limited by the majority of studies in a limited number of ALL cell lines, an unclear relationship between XIAP expression and cell survival and drug resistance both in cell lines and clinical specimens, and some inconsistencies from study to study.

We thank the reviewer for acknowledging that we unequivocally demonstrate a role for XIAP in leukemias. Greatly improving the impact of our findings, we performed molecular *in vivo* studies using PDX models as an important novelty in our paper. Despite technical challenges, we decided studying PDX models because the predictive power of cell line work for therapy response appears limited *(Ben-David et al. 2018)*.
The relationship between XIAP expression and cell survival and drug resistance is indeed complex and controversial in the literature, as we now broadly discuss in the manuscript.

Changes made in the manuscript:
Introduction, line 59ff
Discussion, lines 319ff and 351ff

- Ben-David *et al* (2018): Genetic and transcriptional evolution alters cancer cell line drug response. *Nature* 560: 325-330

Specific comments:
1. It is not clear how the authors conclude that there is no relationship between responses to SMs in Figure 1A and IAP expression as there is no quantification of the western blot in Figure S1B that I can find in documents provided. It would be interesting to know whether the expression level of XIAP correlates with the ability of SMs to sensitizes ALL cells to chemotherapy.

We now performed quantification of the respective western blots and included it into the source data file for Figure S1B. In addition, we correlated western blot results of IAP expression (Appendix S1B) to response to SM (Appendix S1A) for review only, see below. While we observed a positive trend for higher XIAP and c-IAP-2 levels and response to SM, there was no significant or consistent correlation between IAP expression and apoptosis. We state this in the introduction and discussion parts.

Data for reviewer:

Linear regression of specific apoptosis after SM treatment (Data from Appendix Figure S1A, Source data) and IAP protein expression levels (Appendix Figure S1B, Source data).

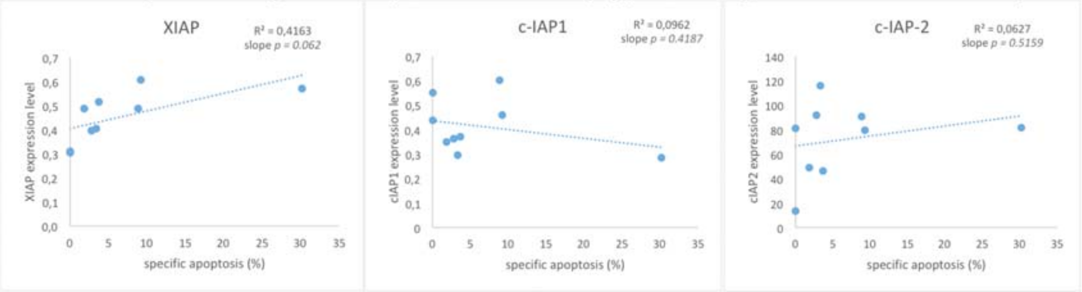

Changes made in the manuscript:

- Source data for Appendix Figure S1B added.
- Introduction, line 59ff
- Discussion line 351ff

2. There is variation in the baseline effect of the vincristine from experiment to experiment, for example in Figure 2, is confusing, and given this variability it is not clear how the replicates are so similar (providing small error bars) within a specific experiment.

Importantly, LBW-mediated sensitization to VCR-induced apoptosis stayed stable between all experiments.

While replicates were performed within a short period of time, most experiments depicted in Figure 2 were performed after cells underwent molecular manipulation. As this required several weeks of cell culture, another batch of vincristine had to be used.

3. It is difficult to interpret the in vivo studies that utilize a mixture of cells with different expression levels of XIAP. It would be more appropriate to carry out conventional studies using a single lines and examining tumor burden following chemotherapeutic treatment rather than comparative levels of cells. Using sufficient sample sizes should be able to overcome mouse-to-mouse variability that is provided as a rationale for these studies. Furthermore, survival analyses would provide the greatest translational relevance.

According to an editorial recommendation, we restricted our revision to discuss benefits and pitfalls of the competitive in vivo analysis and highlight that additional studies like non-competitive survival assays will be helpful to confirm translational relevance.

As stated above in answering the “Comments on Novelty/Model System” of referee 3, competitive in vivo approaches have been wildly used as they increase sensitivity and reliability, while sparing resources, e.g., in mouse studies on normal hematopoiesis (Eaves, 2015, Maetzig et al., 2017). We had recently adapted such assays to PDX leukemia models, as they allow comparing cell populations, which only differ in their genetic manipulation, under the exact identical conditions in the same mouse (Carlet et al., 2021, Liu et al., 2020).

Changes made in the manuscript:
Discussion, line 319ff

- Eaves CJ (2015) Hematopoietic stem cells: concepts, definitions, and the new reality. *Blood* 125: 2605-2613
- Maetzig T, *et al* (2017) Lentiviral Fluorescent Genetic Barcoding for Multiplex Fate Tracking of Leukemic Cells. *Mol Ther Methods Clin Dev* 6: 54-65
- Carlet M, *et al* (2021) In vivo inducible reverse genetics in patients' tumors to identify individual therapeutic targets. *Nat Commun* 12: 5655
- Liu WH, *et al* (2020) Inducible transgene expression in PDX models in vivo identifies KLF4 as a therapeutic target for B-ALL. *Biomark Res* 8: 46

4. If XIAP expression levels are associated with drug resistance, then it would be informative to examine the correlation between expression levels and successful remission induction in clinical specimens.

We re-analyzed our own RNA sequencing data of bone marrow samples from children suffering from relapsed ALL and evaluated XIAP mRNA expression levels. Children with an early onset of relapse had a tendency for higher XIAP expression, albeit not significant. This further supports the notion that XIAP might be of particular importance in aggressive disease subtypes, however, and as now emphasized in the Introduction and Discussion, XIAP expression alone is not predictive of therapy response in clinical specimens.

Data for review only:

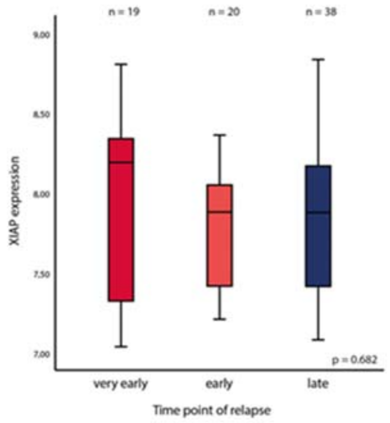

Because the analysis did not reach statistical significance, we did not include the dataset into the manuscript, but are happy to do so at the reviewers discretion.

Changes made in the manuscript:
- Introduction, line 59ff
- Discussion, line 351ff

5. It is not clear whether the specimens in Figure S2A examining newly diagnosed and relapsed are from the same patients, and it would be interesting to know if XIAP expression changes with disease progression.

The samples studied in Figure S2A were from different children, according to sample availability.

Changes made in the manuscript:
Revised legend of Appendix Fig. S2A

6. The use of "normal" PBMCs as a comparator for the XIAP expression studies is not relevant and studies comparing expression with either B or T cell precursors would be more meaningful.

We agree that pre-B cells and pre-T cells may be the best controls for most B- and T-cell leukemias and therefore chose bone marrow samples from healthy donors as controls whenever possible (Figure 3A, EV2B,D and Appendix Figure S2A). However, we do not have a sufficient amount of healthy bone marrow samples available for all our protein analyses so that we had to use PBMC as controls here, which is the current standard in hematopoiesis and leukemia research (Feinberg et al, 2007; Nelson et al, 2021; Niewerth et al, 2016; Wei et al, 2020).

Changes made in the manuscript:

Methods, line 447ff

- Feinberg et al (2007): The Kruppel-like factor KLF4 is a critical regulator of monocyte differentiation. EMBO J 26: 4138-4148
- Nelson et al (2021): Intrinsic OXPHOS limitations underlie cellular bioenergetics in leukemia. Elife 10
- Niewerth et al (2016): Proteasome subunit expression analysis and chemosensitivity in relapsed paediatric acute leukaemia patients receiving bortezomib-containing chemotherapy. Journal of hematology & oncology 9: 82
- Wei et al (2020): Protein tyrosine phosphatase 4A3 (PTP4A3/PRL-3) drives migration and progression of T-cell acute lymphoblastic leukemia in vitro and in vivo. Oncogenesis 9: 6

Referee #4 (Comments on Novelty/Model System for Author):

All categories rated have the potential to improve to "High" with moderate revision as indicated.

We deeply thank the reviewer for acknowledging our work.

Referee #4 (Remarks for Author):

Carlet et al. XIAP targeting in ALL

The manuscript describes experiments to exploit increased expression of IAP proteins in ALL by treating with SMAC mimetic (SM) compounds in combination with classical chemotherapeutics. Using cell lines LOUCY, NALM-6, and patient-donated ALL cells, results show combination efficacy of the SM compounds BV6, LCL and LBW when co-administered with vincristine, doxorubicin, and prednisone. This effect appears to be independent of the function of NFkB and RIPK1 and is characterized by an apoptosis-associated PARP cleavage pattern. Data show that SM compounds reduce cIAP-1 and cIAP-2 without triggering apoptosis, suggesting additional pro-apoptotic activities, and thus XIAP is investigated through pharmacologic inhibitors, knockdown, and over-expression approaches. Nearly complete XIAP knockdown was associated with spontaneous apoptosis in NALM-6 cells in vitro. XIAP knockdown resulted in a growth disadvantage using NALM-6 and patient-donated relapsed ALL cells in competitive transplantation experiments. The authors' conclusion is that XIAP has a yet-to-be-identified, essential function in ALL cells, that may be targetable.

Major Conclusions and General Comments:

1. SMAC inhibitors in combination with classical chemotherapeutics are effective for treatment of ALL in vitro.
   a. Previous reports supporting a similar conclusion should be better presented in the Introduction. Results consistent and different from these previous reports should be discussed directly.

We have revised the text accordingly and moved different paragraphs into introduction and discussion, respectively.

Changes made in the manuscript:
- Introduction, line 59ff
- Discussion, line 351ff

2. XIAP mediates controls apoptosis responses in ALL, independent of a role for cIAP-1/2.
    a. Results dissociate decreased cIAP protein levels from the induction of apoptosis (Appendix Figure S1). Interestingly, XIAP protein levels remain overall unchanged. How these results differ from the reduction of XIAP in response to treatment of ALL cells with Smac mimetic Birinapant (Richmond et al. Cancer Research 2016) should be discussed. While the results support that reduced cIAPs are not sufficient to induce apoptosis, it remains possible that cIAP reduction is a necessary molecular event for apoptosis initiation. These possibilities should be resolved experimentally, or the conclusions need to acknowledge the possible alternative possible interpretations.

The study from Richmond et al. reported Birinapant-sensitive samples where treatment with Birinapant alone induced cIAP-1/-2 and XIAP degradation. In contrast and in the same study, Birinapant-resistant ALL PDX (5 out 8 BPC-ALL PDX, 2 of 3 ETP-ALL PDX) mostly lacked XIAP degradation, similar to NALM-6 and LOUCY cell lines in our study. Given the late onset of XIAP degradation, Richmond and colleagues considered XIAP degradation a side effect rather than being required for apoptosis. Similarly, our data show that for LBW242-mediated sensitization of SM-resistant cells to chemotherapy, XIAP degradation is not required, suggesting that the binding of LBW242 to the BIR3 domain of XIAP might be sufficient to block XIAP function. Regarding cIAP1/2, we now highlight that degradation might be supportive, although not always sufficient for cell death.

Changes made in the manuscript:
- Results, line 138
- Discussion, line 341ff and 351ff

3. XIAP regulates ALL apoptosis in vitro and leukemogenesis in vivo.
    a. The conclusion is potentially strongly supported by competitive transplantation experiments that were well controlled and that potentially demonstrate the role of XIAP in ALL apoptosis control (Figures 4-5). However, the specificity of the XIAP knockdown needs to be validated at least in vitro through reconstitution of XIAP using shRNA-resistant re-expression constructs.

We now re-constituted sh-XIAP NALM-6 cells with a plasmid expressing XIAP and performed a competitive growth assay *in vitro*. New Figure 4I plus Addpendix Figure 3I show that re-constitution with XIAP reverses cell death induced by XIAP knockdown, confirming that loss of XIAP is responsible for the loss of fitness in leukemia cells upon knockdown.

Changes made in the manuscript:
- New Figures 4I and text in line 263.
- New Appendix Figure 3I.
- Respective Source data.

4. Manuscript revision.
    a. Some comments from previous review require further revision, as noted.

We have now addressed all specific comments.

5. Overall evaluation.
   a. The manuscript has the potential to advance the possibility of targeting XIAP for ALL therapy, with revisions to strengthen major conclusions and address minor issues as indicated.

We thank the reviewer for acknowledging our work and the very helpful comments that have significantly improved our manuscript.

Specific comments

1. The presentation of Figure S1A is confusing. It is not clear why MOLT4 and MOLT3 are not pursued. (Manuscript revision).

Molt-3 and Molt-4 were not further examined because they were completely resistant towards LBW242 and therefore not suitable to model sensitization by LBW in ALL.

Changes made in the manuscript:
Results, line 93: "*The two ALL cell lines (Molt-3 and Molt-4) were completely resistant towards LBW242 (Appendix Figure S1A) and were excluded from further studies*."

2. In Figure EV1C-D, a condition testing the efficacy of anti-TNFa neutralizing Ab adalimumab in TNFa-treated cells is necessary to support the stated conclusion that SM LBW is functioning in a TNFa-independent fashion in the cells tested.

For LOUCY cells, this control is included in Fig. EV1A. We now performed the experiment also in NALM6 cells.

Changes made in the manuscript:
- New Figure EV1D and results, line 142ff

Please check: the text references appear to indicate that EV1C will present ALL samples when in fact it shows NALM-6 cells, and vice versa for EV1D.

We thank the reviewer for pointing this out, the text has been changed accordingly.

3. Figure 2D, a positive control would be required to support the conclusion of NEMO-independent apoptosis in response to SM, considering the incomplete knockdown of NEMO (62%). Nevertheless, what the experiment would contribute to the analysis, as the expected effect of NEMO knockdown would be increased apoptosis (if any effect is observed).

We understand the reviewers concern that incomplete knockdown efficiency of NEMO might be insufficient to affect NFkB activity. Literature provides evidence that a knockdown efficiency of approx. 60% is sufficient for NEMO to affect NFkB activation *(Elsarraj et al, 2017; Konig et al, 2012)* and state this in the manuscript.

Changes made in the manuscript:
Results, line 165ff: "*Here, the SM-drug combination did not induce degradation of IκBα (Figure 2C) and apoptosis was not affected by KD of NEMO to levels that were shown to block NF-κB activation (Figure 2D) (Elsarraj et al, 2017; Konig et al, 2012).*"

- Elsarraj et al (2017) NEMO, a Transcriptional Target of Estrogen and Progesterone, Is Linked to Tumor Suppressor PML in Breast Cancer. Cancer research 77: 3802-3813
- Konig et al (2012) Fibroblast growth factor homologous factor 1 interacts with NEMO to regulate NF-kappaB signaling in neurons. Journal of cell science 125: 6058-6070

4. Figure EV2. Please indicate statistical significance (or not) in the data shown in panels C and E. (Manuscript revision).

Changes made in the manuscript:
Statistical significance is now indicated in revised Figure EV2A,C and E

5. Appendix Figure S2, there is not a control that demonstrates that ARTS A4 is functioning in an XIAP-dependent fashion.

We performed new experiments and now show that ARTS A4 treatment decreased XIAP levels in NALM-6 and LOUCY cells. As this represents an indication, but no proof, we toned down our statements in the main text.
We had introduced the ARTS mimetic A4 based on a previous reviewers´ suggestion which was based on literature showing that ARTS A4 targets and decreases protein levels of XIAP, but not c-IAP1 (Mamriev et al. 2020).
Changes made in the manuscript:
- New Appendix Figure 2C
- Line 199ff

- Mamriev et al (2020) A small-molecule ARTS mimetic promotes apoptosis through degradation of both XIAP and Bcl-2. Cell death & disease 11: 483

6. Appendix Figure S3A: The legend and labeling of the figure does not sufficiently present the system being used. The first two rows of data are using which shRNA(s)? The GFP reporter shown in the data is not described in the legend. The legend indicates that the "expression of the dTomato is reduced," but this description does not match the data.

We thank the reviewer for pointing this out. We revised the legend of Figure S3A to enhance the understanding of the Figure.

Changes made in the manuscript:
Revised Legend Figure S3A
“The shRNA reporter system (Fellmann et al, 2013) enables to determine the efficiency of shRNA sequences. Multiple genomic shRNA target sequences were cloned into the 3’UTR of a dTomato marker gene and constitutively expressed in NALM-6 cells (red). dTomato-positive NALM-6 cells were subsequently transduced with a second lentiviral vector expressing a specific mir30-shRNA sequence and eGFP as marker for transduction. Reduced expression of dTomato intensity in the GFP/dTomate double-positive population takes place only if the shRNA sequence/eGFP recognizes the specific target sequence located in the 3’UTR of dTomato. Representative flow cytometry plots of NALM-6 (untransduced, transduced with experimental targets, empty mir30, strong or weak shRNA) are shown. Quantification of KD efficiency is summarized and plotted as % of KD efficiency using shRenilla as CTRL. shXIAP.1 and shXIAP.2 sequences have been selected for further studies.”

9th Sep 2022

Dear Prof. Jeremias,

Thank you for the submission of your revised manuscript to EMBO Molecular Medicine and please accept my apologies for the delay in getting back to you as the reports came in when I was on annual leave. We have received the enclosed reports from the two referees who re-reviewed your manuscript. As you will see, they are now supportive of publication, and I am therefore pleased to inform you that we will be able to accept your manuscript once the following editorial points will be addressed:

1/ Referees:
- Please address the remaining minor concern from referee #4.

2/ Main manuscript text:
- Material and methods:
o Please include the full sentence that that the experiments conformed to the principles set out in the WMA Declaration of Helsinki and the Department of Health and Human Services Belmont Report.
o Please indicate whether the cells were tested for mycoplasma contamination.
o Please provide the antibody dilutions
- Thank you for providing the Data Availability section. Please provide an URL in this section.
- Author contribution: CRediT has replaced the traditional author contributions section because it offers a systematic machine-readable author contributions format that allows for more effective research assessment. Please remove the Authors Contributions from the manuscript and use the free text boxes beneath each contributing author's name in our system to add specific details on the author's contribution. More information is available in our guide to authors.
- References: please update the format to have 10 authors before et al.

3/ Figures and Appendix:
- Please provide exact p values in all figures or in their legends. Some people found that to keep the figures clear, providing a supplemental table with all exact p-values was preferable. You are welcome to do this if you want to.
- Fig 3B: please remove in the legend +/-SD as error bars were removed.

4/ Thank you for providing a checklist. We have however changed the format of this checklist earlier this year, and I would kindly ask you to complete the new checklist, which you can download from our author guidelines (https://www.embopress.org/page/journal/17574684/authorguide#submissionofrevisions). Please insert information in the checklist that is also reflected in the manuscript. The completed author checklist will also be part of the RPF.

5/ Thank you for providing a synopsis. Pease resize your graphical abstract to 550 px wide x 300-600 px high. Please note that these would be the final versions (both text and graphic) and changes during proofing are usually not allowed.

We note that you agree with the publication of the Review Process File (RPF) as is.

I look forward to receiving your revised manuscript.

With kind regards,

Lise Roth

Lise Roth, PhD

Senior Editor

EMBO Molecular Medicine

***** Reviewer's comments *****

Referee #4 (Comments on Novelty/Model System for Author):

Controls added to enhance technical quality include:
- Expression of an XIAP rescue construct which counteracted apoptosis in shXIAP NALM6 cells.
- Analysis of TNFa neutralizing Ab effects in NALM6 cells treated with LBW +/- vin.
- Analysis of XIAP levels following treatment with ARTS A4.

Novelty is improved through modifications that better distinguish work described here from prior literature, with extensive citations.

Medical impact is sufficient.

Model systems sufficiently support conclusions.

Referee #4 (Remarks for Author):

The figure legend improvements for Appendix Figure 3A are helpful. However, it may be clearer to indicate that the gray and red populations are repeated in all panels as references for comparison to the dTomato/eGFP double positive population (shown in green).

The authors addressed the minor editorial issues.

28th Oct 2022

Dear Prof. Jeremias,

Thank you for sending the revised Appendix. I am pleased to inform you that your manuscript is now accepted for publication and will be sent to our publisher to be included in the next available issue of EMBO Molecular Medicine.

Congratulations on your interesting work!

With kind regards,

Lise Roth

Lise Roth, Ph.D
Senior Editor
EMBO Molecular Medicine

Follow us on Twitter @EmboMolMed
Sign up for eTOCs at embopress.org/alertsfeeds

EMBO Press Author Checklist

Corresponding Author Name: Prof. Dr. Irmela Jeremias	
Journal Submitted to: EMBO Molecular Medicine	
Manuscript Number: EMM-2021-14557	

USEFUL LINKS FOR COMPLETING THIS FORM
The EMBO Journal - Author Guidelines
EMBO Reports - Author Guidelines
Molecular Systems Biology - Author Guidelines
EMBO Molecular Medicine - Author Guidelines

Reporting Checklist for Life Science Articles (updated January 2022)
This checklist is adapted from Materials Design Analysis Reporting (MDAR) Checklist for Authors. MDAR establishes a minimum set of requirements in transparent reporting in the life sciences (see Statement of Task: 10.31222/osf.io/9sm4x). Please follow the journal's guidelines in preparing your manuscript.
Please note that a copy of this checklist will be published alongside your article.

Abridged guidelines for figures
1. Data
The data shown in figures should satisfy the following conditions:
- the data were obtained and processed according to the field's best practice and are presented to reflect the results of the experiments in an accurate and unbiased manner.
- ideally, figure panels should include only measurements that are directly comparable to each other and obtained with the same assay.
- plots include clearly labeled error bars for independent experiments and sample sizes. Unless justified, error bars should not be shown for technical replicates.
- if n<5, the individual data points from each experiment should be plotted. Any statistical test employed should be justified.
- Source Data should be included to report the data underlying figures according to the guidelines set out in the authorship guidelines on Data Presentation.

2. Captions
Each figure caption should contain the following information, for each panel where they are relevant:
- a specification of the experimental system investigated (eg cell line, species name).
- the assay(s) and method(s) used to carry out the reported observations and measurements.
- an explicit mention of the biological and chemical entity(ies) that are being measured.
- an explicit mention of the biological and chemical entity(ies) that are altered/varied/perturbed in a controlled manner.
- the exact sample size (n) for each experimental group/condition, given as a number, not a range;
- a description of the sample collection allowing the reader to understand whether the samples represent technical or biological replicates (including how many animals, litters, cultures, etc.).
- a statement of how many times the experiment shown was independently replicated in the laboratory.
- definitions of statistical methods and measures:
  - common tests, such as t-test (please specify whether paired vs. unpaired), simple χ2 tests, Wilcoxon and Mann-Whitney tests, can be unambiguously identified by name only, but more complex techniques should be described in the methods section;
  - are tests one-sided or two-sided?
  - are there adjustments for multiple comparisons?
  - exact statistical test results, e.g., P values = x but not P values < x;
  - definition of 'center values' as median or average;
  - definition of error bars as s.d. or s.e.m.

Please complete ALL of the questions below.
Select "Not Applicable" only when the requested information is not relevant for your study.

Materials

Newly Created Materials	Information included in the manuscript?	In which section is the information available? (Reagents and Tools Table, Materials and Methods, Figures, Data Availability Section)
New materials and reagents need to be available; do any restrictions apply?	Not Applicable	

Antibodies	Information included in the manuscript?	In which section is the information available? (Reagents and Tools Table, Materials and Methods, Figures, Data Availability Section)
For **antibodies** provide the following information:<br>- Commercial antibodies: RRID (if possible) or supplier name, catalogue number and or/clone number<br>- Non-commercial: RRID or citation	Yes	Materials and Methods

DNA and RNA sequences	Information included in the manuscript?	In which section is the information available? (Reagents and Tools Table, Materials and Methods, Figures, Data Availability Section)
**Short novel DNA or RNA including primers, probes**: provide the sequences.	Yes	Materials and Methods

Cell materials	Information included in the manuscript?	In which section is the information available? (Reagents and Tools Table, Materials and Methods, Figures, Data Availability Section)
**Cell lines:** Provide species information, strain. Provide accession number in repository **OR** supplier name, catalog number, clone number, and/**OR** RRID.	Yes	Materials and Methods
**Primary cultures:** Provide species, strain, sex of origin, genetic modification status.	Yes	Table EV1
Report if the cell lines were recently **authenticated** (e.g., by STR profiling) and tested for mycoplasma contamination.	Yes	Materials and Methods

Experimental animals	Information included in the manuscript?	In which section is the information available? (Reagents and Tools Table, Materials and Methods, Figures, Data Availability Section)
**Laboratory animals or Model organisms:** Provide species, strain, sex, age, genetic modification status. Provide accession number in repository **OR** supplier name, catalog number, clone number, **OR** RRID.	Yes	Materials and Methods
**Animal observed in or captured from the field:** Provide species, sex, and age where possible.	Not Applicable	
Please detail **housing and husbandry conditions**.	Yes	Materials and Methods

Plants and microbes	Information included in the manuscript?	In which section is the information available? (Reagents and Tools Table, Materials and Methods, Figures, Data Availability Section)
**Plants:** provide species and strain, ecotype and cultivar where relevant, unique accession number if available, and source (including location for collected wild specimens).	Not Applicable	
**Microbes:** provide species and strain, unique accession number if available, and source.	Not Applicable	

Human research participants	Information included in the manuscript?	In which section is the information available? (Reagents and Tools Table, Materials and Methods, Figures, Data Availability Section)
If collected and within the bounds of privacy constraints report on age, sex and gender or ethnicity for all study participants.	Yes	Materials and Methods

Core facilities	Information included in the manuscript?	In which section is the information available? (Reagents and Tools Table, Materials and Methods, Figures, Data Availability Section)
If your work benefited from core facilities, was their service mentioned in the acknowledgments section?	Yes	Acknowledgements

Design

Study protocol	Information included in the manuscript?	In which section is the information available? (Reagents and Tools Table, Materials and Methods, Figures, Data Availability Section)
If study protocol has been **pre-registered, provide DOI in the manuscript**. For clinical trials, provide the trial registration number **OR** cite DOI.	Not Applicable	
Report the **clinical trial registration number** (at ClinicalTrials.gov or equivalent), where applicable.	Not Applicable	

Laboratory protocol	Information included in the manuscript?	In which section is the information available? (Reagents and Tools Table, Materials and Methods, Figures, Data Availability Section)
Provide DOI OR other citation details if **external detailed step-by-step protocols** are available.	Yes	Materials and Methods

Experimental study design and statistics	Information included in the manuscript?	In which section is the information available? (Reagents and Tools Table, Materials and Methods, Figures, Data Availability Section)
Include a statement about **sample size** estimate even if no statistical methods were used.	Yes	Materials and Methods
Were any steps taken to minimize the effects of subjective bias when allocating animals/samples to treatment (e.g. **randomization procedure**)? If yes, have they been described?	Not Applicable	
Include a statement about **blinding** even if no blinding was done.	Yes	Materials and Methods
Describe **inclusion/exclusion criteria** if samples or animals were excluded from the analysis. Were the criteria pre-established?<br><br>If sample or data points were omitted from analysis, report if this was due to attrition or intentional exclusion and provide justification.	Not Applicable	
For every figure, are **statistical tests** justified as appropriate? Do the data meet the assumptions of the tests (e.g., normal distribution)? Describe any methods used to assess it. Is there an estimate of variation within each group of data? Is the variance similar between the groups that are being statistically compared?	Yes	Materials and Methods, Figures Legends

Sample definition and in-laboratory replication	Information included in the manuscript?	In which section is the information available? (Reagents and Tools Table, Materials and Methods, Figures, Data Availability Section)
In the figure legends: state number of times the experiment was **replicated** in laboratory.	Yes	Figure Legends
In the figure legends: define whether data describe **technical or biological replicates**.	Yes	Figure Legends

Ethics

Ethics	Information included in the manuscript?	In which section is the information available? (Reagents and Tools Table, Materials and Methods, Figures, Data Availability Section)
Studies involving **human participants**: State details of **authority granting ethics approval** (IRB or equivalent committee(s), provide reference number for approval.	Yes	Materials and Methods
Studies involving **human participants**: Include a statement confirming that **informed consent** was obtained from all subjects and that the experiments conformed to the principles set out in the WMA Declaration of Helsinki and the Department of Health and Human Services Belmont Report.	Yes	Materials and Methods
Studies involving **human participants:** For publication of **patient photos**, include a statement confirming that consent to publish was obtained.	Not Applicable	
Studies involving experimental **animals**: State details of **authority granting ethics approval** (IRB or equivalent committee(s), provide reference number for approval. Include a statement of compliance with ethical regulations.	Yes	Materials and Methods
Studies involving **specimen and field samples:** State if relevant **permits** obtained, provide details of authority approving study; if none were required, explain why.	Not Applicable	

Dual Use Research of Concern (DURC)	Information included in the manuscript?	In which section is the information available? (Reagents and Tools Table, Materials and Methods, Figures, Data Availability Section)
Could your study fall under dual use research restrictions? Please check biosecurity documents and list of **select agents and toxins** (CDC): https://www.selectagents.gov/sat/list.htm	Not Applicable	
If you used a select agent, is the security level of the lab appropriate and reported in the manuscript?	Not Applicable	
If a study is subject to dual use research of concern regulations, is the name of the **authority granting approval and reference number** for the regulatory approval provided in the manuscript?	Not Applicable	

Reporting

The MDAR framework recommends adoption of discipline-specific guidelines, established and endorsed through community initiatives. Journals have their own policy about requiring specific guidelines and recommendations to complement MDAR.

Adherence to community standards	Information included in the manuscript?	In which section is the information available? (Reagents and Tools Table, Materials and Methods, Figures, Data Availability Section)
State if relevant guidelines or checklists (e.g., **ICMJE, MIBBI, ARRIVE, PRISMA**) have been followed or provided.	Not Applicable	
For **tumor marker prognostic studies**, we recommend that you follow the **REMARK** reporting guidelines (see link list at top right). See author guidelines, under 'Reporting Guidelines'. Please confirm you have followed these guidelines.	Not Applicable	
For **phase II and III randomized controlled trials**, please refer to the **CONSORT** flow diagram (see link list at top right) and submit the CONSORT checklist (see link list at top right) with your submission. See author guidelines, under 'Reporting Guidelines'. Please confirm you have submitted this list.	Not Applicable	

Data Availability

Data availability	Information included in the manuscript?	In which section is the information available? (Reagents and Tools Table, Materials and Methods, Figures, Data Availability Section)
Have **primary datasets** been deposited according to the journal's guidelines (see 'Data Deposition' section) and the respective accession numbers provided in the Data Availability Section?	Yes	Data Availability Section
Were **human clinical and genomic datasets** deposited in a public access-controlled repository in accordance to ethical obligations to the patients and to the applicable consent agreement?	Yes	Materials and Methods, Data Availability Section
Are **computational models** that are central and integral to a study available without restrictions in a machine-readable form? Were the relevant accession numbers or links provided?	Not Applicable	
If publicly available data were reused, provide the respective **data citations in the reference list**.	Not Applicable	
