# Supplementary material for: X‐linked inhibitor of apoptosis protein represents a promising therapeutic target for relapsed/refractory ALL
Source: EMBO Mol Med. 2022 Nov 23;15(1):e14557. doi: 10.15252/emmm.202114557 (PMC9832863; doi:10.15252/emmm.202114557)

Fig. S1B

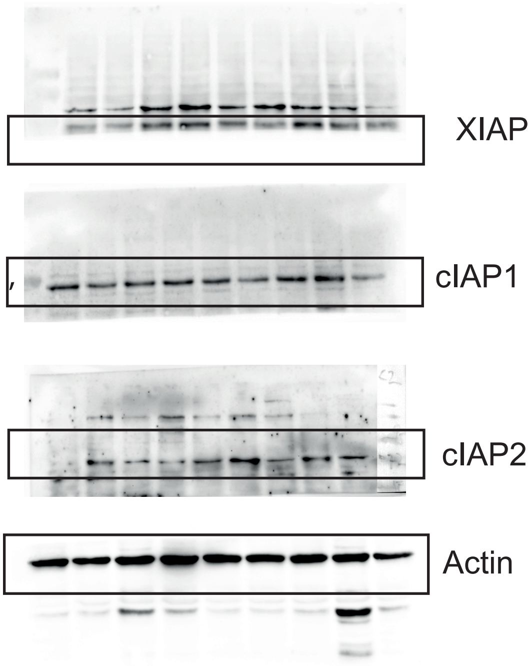

Supplement: Supplementary file 5 — Source Data for Expanded View and Appendix [file EMMM-15-e14557-s004.zip › EMM-2021-14557-V3-Appendix_Source_Data-sd.zip!/EMM-2021-14557_Source data_Appendix/Fig.AppendixS1B.pdf]
